# Threatened Reef Corals of the World

**DOI:** 10.1371/journal.pone.0034459

**Published:** 2012-03-30

**Authors:** Danwei Huang

**Affiliations:** 1 Scripps Institution of Oceanography, University of California San Diego, La Jolla, California, United States of America; 2 Department of Biological Sciences, National University of Singapore, Singapore, Singapore; University of Texas, United States of America

## Abstract

A substantial proportion of the world's living species, including one-third of the reef-building corals, are threatened with extinction and in pressing need of conservation action. In order to reduce biodiversity loss, it is important to consider species' contribution to evolutionary diversity along with their risk of extinction for the purpose of setting conservation priorities. Here I reconstruct the most comprehensive tree of life for the order Scleractinia (1,293 species) that includes all 837 living reef species, and employ a composite measure of phylogenetic distinctiveness and extinction risk to identify the most endangered lineages that would not be given top priority on the basis of risk alone. The preservation of these lineages, not just the threatened species, is vital for safeguarding evolutionary diversity. Tests for phylogeny-associated patterns show that corals facing elevated extinction risk are not clustered on the tree, but species that are susceptible, resistant or resilient to impacts such as bleaching and disease tend to be close relatives. Intensification of these threats or extirpation of the endangered lineages could therefore result in disproportionate pruning of the coral tree of life.

## Introduction

Worldwide, ocean-scale effects of sea surface warming and acidification are subjecting reef corals to severe stresses, resulting in intensified bleaching and disease, as well as declining calcification rates [1]–[6]. Local anthropogenic impacts such as overfishing and pollution have also forced coral reefs through regime shifts toward macroalgal domination [4], [7]–[10]. Alarmingly, 32.8% of all zooxanthellate reef-building coral species are considered to be threatened with global extinction [11] (see also [12]).

Limited resources constrain scientists and managers to focus their efforts on a subset of the world's coral reefs to minimise extinction risk [13]. Consequently, the decision-making process associated with assignment of funds and manpower has become a major research focus in conservation biology [14]–[17]. One of the most widely-used frameworks for assessing threats to species and setting conservation priorities is the International Union for Conservation of Nature (IUCN) Categories and Criteria [18], [19]. Indeed, the identification and design of protected areas are often guided by the distribution of species with the highest risk of extinction, and in particular, the most threatened species of the IUCN Red List [20]–[22].

Extinction probabilities aside, species are not equal. Rather, evolutionary processes render each species unique with a characteristic history that can be quantified for the purpose of conservation prioritisation [14], [23]–[26]. Assessments that integrate phylogenetic distinctiveness and extinction threat have been performed mainly for mammalian groups, drawing attention to extraordinary species from lesser known localities and lineages (i.e. lines of ancestry and descent [27]) [28]–[33]. The dire situation of reef corals necessitates an equivalent treatment.

The utility of phylogenetic trees extends beyond the recognition of distinct lineages that are at risk. Due to the hierarchical nature of phylogenies, random losses of species rarely perturb the branches of evolutionary history [34], but concentration of threatened species or risk factors in particular parts of the phylogeny can imperil entire clades [35]–[38]. Threats to reef corals have traditionally been generalised based on species' taxonomic memberships [39], [40]. The family Faviidae, for instance, is reputed to be resilient to environmental disturbances [41], but the extreme polyphyly of the group has called into question such inferences [42] (see also [43]). Considering evolutionary history in the analysis of extinction risk will certainly aid in the development of informed conservation strategies against threats facing corals of the world today.

The aim of this study is to apply the phylogenetic approach on all reef corals of the order Scleractinia to identify, first, the most endangered coral lineages, and second, evolutionary patterns associated with extinction probability and various threats. To rank corals according to both distinctiveness and imperilment, I use the EDGE (evolutionarily distinct and globally endangered) metric [29], which combines a unique measure of phylogenetic diversity [44] with the conservation status of each species. Data for the latter are based on the IUCN Red List that includes 827 reef-building scleractinians assessed by the world's leading coral experts in 2006 and 2007 [11]. Of the 688 species not deemed Data Deficient (DD), 32.7% are considered threatened. These comprise―in decreasing likelihood of extinction―four Critically Endangered (CR), 23 Endangered (EN) and 198 Vulnerable (VU) corals. The remaining species are categorised as Near Threatened (NT; 174 species) or of Least Concern (LC; 289 species).

## Methods

### Phylogenetic data and analyses

To reconstruct the scleractinian phylogeny, 827 species from the IUCN Red List dataset [11], five previously omitted corals, five new species described since the assessment [45]–[47], and 65% of non-reef corals [48] were included in the analysis (Table S1). The supertree approach [49], [50] was used to combine data from molecular, morphologic and taxonomic sources. Unlike Kerr [51], the last published Scleractinia supertree, I reanalysed the molecular data rather than use available phylogenies because several DNA markers were utilised repeatedly in different studies (e.g. [52] and [53]). Using these phylogenies as source trees would result in data duplication [54], [55].

Mitochondrial DNA markers each with coverage of >40 species were obtained from GenBank to assemble a 463-species dataset (365 reef, 98 non-reef). The seven markers used were 12S small subunit ribosomal RNA (12S), 16S ribosomal RNA (16S), ATP synthase F0 subunit 6 (AT6), cytochrome c oxidase subunit I (COI), control region (CTR), cytochrome b (CYB) and NADH dehydrogenase subunit 5 (ND5) (Table S1). Corallimorphs *Discosoma* and *Ricordea florida* were included as outgroups. Matrices were aligned with MAFFT 6.8 [56], [57] and concatenated for analysis under the maximum likelihood criterion, using RAxML 7.2.8 [58], [59] implemented at the Cyberinfrastructure for Phylogenetic Research (http://www.phylo.org) [60]. Tree searches were carried out in 1000 alternate runs from distinct parsimony starting trees, utilising the partitioned GTRGAMMA model. Nodal supports were assessed via 1000 bootstrap replicates.

Thirteen morphological datasets were used to obtain source trees for the supertree reconstruction [61]–[69] (Table 1). All except one [61] were included in Kerr's [51] study. Congeners were assumed monophyletic unless otherwise shown in recent phylogenies (see remarks, Table S1). Maximum parsimony analyses were performed in PAUP* 4.0b10 [70] using the branch-and-bound algorithm for matrices with ≤25 terminals and heuristic searches (10^5^ random additions with a rearrangement limit of 10^7^ per replicate) for larger datasets. Nodal supports were determined with 1000 bootstrap replicates (100 random additions per replicate for heuristic searches). For 145 reef species with no available data, a source tree was used to represent likely sister relationships based on a review of literature, favouring the more recent hypotheses in cases of conflict [71]–[97] (see remarks, Table S1).

**Table 1:** Morphological data used as source matrices for supertree reconstruction.

Taxon	No. of genera	No. of species	Analysis parameters	Reference
Faviina	11	**26**	equal weights; unordered	[61]
Turbinoliidae	**22**	57	characters weighted; one character ordered	[62]
Dendrophylliidae	**20**	164	characters weighted; two characters ordered	[63]
Scleractinia	**29**	440	equal weights; unordered	[64]
Fungiidae	15	**40**	equal weights; unordered	[65]
*Pleuractis*	1	**6**	equal weights; unordered	[66]
Mussidae	**12**	44	characters weighted; Lundberg rooting	[67]
*Lobophyllia*+*Symphyllia*	2	**10**	characters weighted	[67]
Siderastreidae	**6**	29	characters weighted; Lundberg rooting	[67]
*Coscinaraea*+*Psammocora*	2	**14**	characters weighted	[67]
Scleractinia+Corallimorpharia	38	**47**	includes two outgroups	[68]
Acroporidae	**6**	291	equal weights; unordered	[69]
*Acropora*+*Isopora*	2	**139**	10 sister species grafted post-analysis	[69]

Numbers in bold represent the taxonomic levels of analyses performed in the original studies.

Including the molecular phylogeny, 1293 scleractinian species (837 reef, 456 non-reef) were analysed. All source trees were coded into bootstrap percentage-weighted matrix representation with parsimony using SuperMRP 1.2.1 [98]. To ensure that analyses were driven primarily by data, weights of nodes derived from taxonomic information were each set at one. Maximum parsimony analysis of the 792-character dataset was carried out as above (rearrangement limit of 10^8^ per replicate) to obtain 18978 minimum length trees.

The molecular data were then fitted to the strict consensus supertree using RAxML (1000 replicate runs) to derive the best branch length estimates [99]. Polytomies in the supertree were randomly resolved to generate 1000 different resolutions. Species with no available DNA sequence data were assigned a terminal branch length of zero, though still represented by their ancestral branches based on topology. This procedure yielded estimates for the lower limit of distinctiveness, a conservative approach given the lack of data. Calculations that followed were carried out for each of the 1000 resolutions; reported results are means over all randomly resolved trees.

### Determining species priorities

For each reef species in the Scleractinia supertree, Tuatara 1.01 [100] was used to evaluate evolutionary distinctiveness (ED) by summing the terminal branch length and its species-weighted allocation of ancestral branches. ED was then multiplied by extinction probability (PE) to obtain the EDGE score, a measure of expected loss of evolutionary history [29], [101]. PE was calculated based on the IUCN100 transformation of the Red List categories [102]. LC species' PE was set at 0.001, assuming that at most about one of the 289 LC corals would go extinct in 100 years; NT corals were given an intermediate PE of 0.01. For the 149 DD species, a PE value between the lowest Red List categories (LC and NT) was assigned [33]. The ‘Isaac’ and ‘Pessimistic’ transformations of Mooers et al. [102] led to an LC species consistently achieving the top two highest scores, an overly conservative result that is not discussed (available in Table S1). Species were ranked according to their EDGE scores. Analyses repeated exclusively for the reef species show that incomplete sampling of Scleractinia (i.e. the non-reef corals) had minimal effect (mean rank variation: top 30 species = 1.5, all 837 species = 12.8).

### Testing for phylogenetic signal

Phylogenetic signal of PE was tested using a randomisation procedure [103] in R package Picante 1.3 [104] that determined whether the actual phylogeny better fits a set of continuous data relative to data that had been randomly permuted across the tips of the tree (1000 replicates per supertree; K = 0 for random traits). For binary traits, Fritz & Purvis' [105] D was computed in CAIC 1.0.4 [106]. This metric was based on the trait's sum of sister-clade disparities on the tree (D = 0 for clumped traits, D = 1 for random traits). The phylogenetic patterns of three extinction risk levels, EN and above, VU and above and at least NT, were determined. In addition, eight species-specific binary traits assessed by Carpenter et al. [11] were tested for phylogenetic signal (Table 2).

**Table 2:** Phylogenetic signal of IUCN Red List categories and traits of reef corals.

Category/trait	Proportion of species	D	P for H_0_: D = 0	P for H_0_: D = 1
Endangered and above	0.032	1.096±0.063	<0.001	**0.780**
Vulnerable and above	0.269	0.960±0.023	<0.001	**0.167**
Near Threatened and above	0.477	0.853±0.018	<0.001	<0.001
moderately or highly susceptible to bleaching	0.419	0.229±0.010	<0.001	<0.001
moderately or highly resistant to bleaching	0.116	0.300±0.023	0.001	<0.001
moderately or highly susceptible to disease	0.310	0.124±0.012	0.024	<0.001
moderately or highly resistant to disease	0.058	−0.172±0.015	**0.887**	<0.001
recovers quickly from bleaching or disease	0.134	0.125±0.013	**0.068**	<0.001
moderately or highly susceptible to crown-of-thorns seastar predation	0.273	0.052±0.011	**0.180**	<0.001
restricted or highly fragmented range	0.124	1.136±0.037	<0.001	**0.973**
reported collection of > 1000 pieces per year	0.157	0.630±0.021	<0.001	<0.001

Results based on D, a measure of total sister-clade disparities on the phylogeny (± SD; 0 for clumped traits, 1 for random traits). Numbers in bold denote non-significant results (i.e. not different from 0 or 1).

Two potential confounding factors associated with the above analyses were investigated. First, species assembled in the supertree differ in the degree of representation among source trees. It may be argued that poorly-sampled species are generally placed, unresolved, outside of clades with well-sampled species, leading to bias in calculations. The 1000 random resolutions of the strict consensus supertree should circumvent this problem, but to be sure, the tests were repeated for two reduced datasets with species present in at least two and three source trees respectively. Second, the level of phylogenetic signal inferred for each trait may be influenced by variation in species abundances, hence the analyses were also performed separately for species that are considered common (including one abundant taxon), uncommon and rare (data from [11]). Phylogenetic signal of the trait ‘reported collection of >1000 pieces per year’ for the ‘rare’ dataset could not be computed as it is represented by just two species.

Carpenter et al. [11] also found that several taxa that are susceptible to bleaching also appear to be heavily impacted by disease and predation by the crown-of-thorns seastar, *Acanthaster planci*. To ascertain if this relationship holds with the incorporation of phylogenetic information, I tested for correlation among traits associated with coral bleaching, disease and predation using phylogenetically independent contrasts [107]. This was implemented in APE 2.7 [108], with statistical significance evaluated based on fit to a linear model.

Finally, I determined whether the decrease in phylogenetic diversity (PD) [44] under various extinction scenarios was different from a null model of random extinction. PD was compared between rarefied trees based on threat status (EN and above, VU and above, NT and above) and 1000 randomly pruned trees with the same species richness, using the one-sample t-test [109]. This analysis was also carried out for 30 species with the highest EDGE scores.

## Results

**Figure 1:**
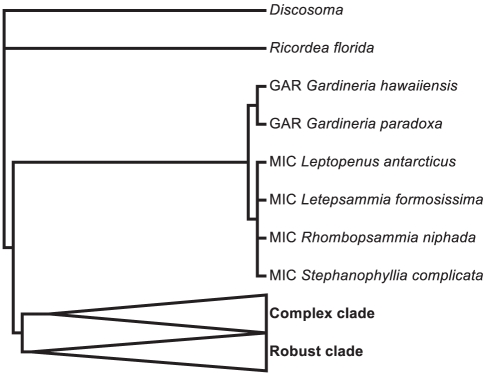
Supertree of Scleractinia with corallimorph outgroups *Discosoma* and *Ricordea florida*. Cladogram of 1293 corals inferred by maximum parsimony analysis of the 792-character dataset assembled using 15 source trees (13 morphological, one molecular and one taxonomic). Complex and robust clades shown in Figures 2 and 3 respectively. GAR: Gardineriidae, MIC: Micrabaciidae.

**Figure 2:**
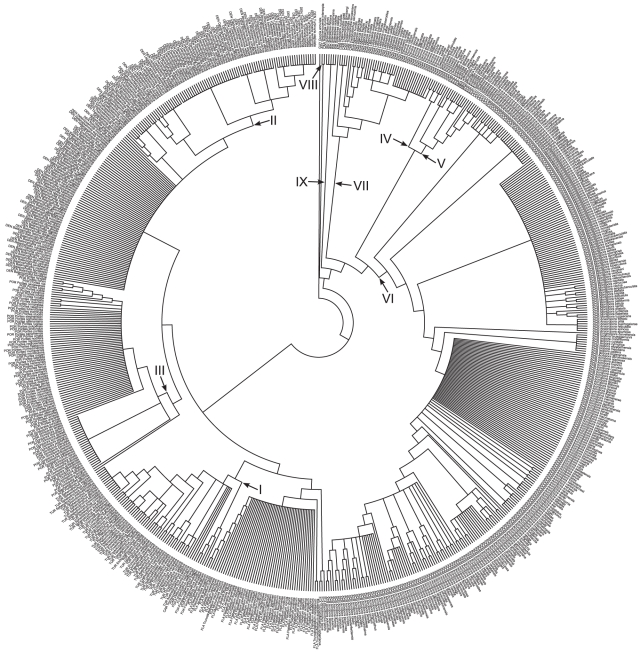
Cladogram of scleractinian corals in the complex clade. A total of 735 corals, including 462 reef species, are represented on this maximum parsimony cladogram that is part of the scleractinian supertree (Figure 1). Roman numerals denote clades based on the phylogeny in Fukami et al. [42]. ACR: Acroporidae, AGA: Agariciidae, AST: Astrocoeniidae, CAR: Caryophylliidae, DEN: Dendrophylliidae, EUP: Euphylliidae, FLA: Flabellidae, FUA: Fungiacyathidae, GUY: Guyniidae, MEA: Meandrinidae, OCU: Oculinidae, POR: Poritidae, SID: Siderastreidae, TUR: Turbinoliidae.

**Figure 3:**
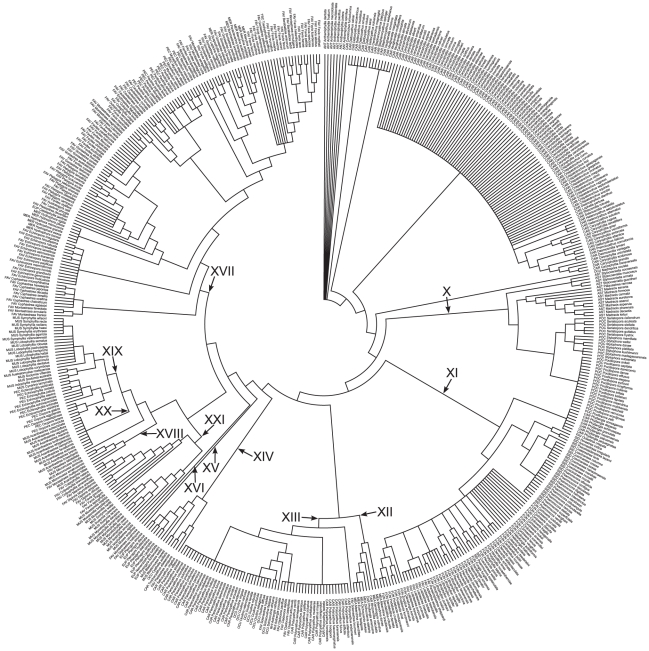
Cladogram of scleractinian corals in the robust clade. A total of 552 corals, including 375 reef species, are represented on this maximum parsimony cladogram that is part of the scleractinian supertree (Figure 1). Roman numerals denote clades based on the phylogeny in Fukami et al. [42]. ANT: Anthemiphyllidae, AST: Astrocoeniidae, CAR: Caryophylliidae, EUP: Euphylliidae, FAV: Faviidae, FUN: Fungiidae, MEA: Meandrinidae, MER: Merulinidae, MUS: Mussidae, OCU: Oculinidae, PEC: Pectiniidae, POC: Pocilloporidae, RHI: Rhizangiidae, SID: Siderastreidae, STE: Stenocyathidae, TRC: Trachyphylliidae.

Integrating the diverse data types using a supertree approach yields a 1293-species phylogeny of Scleractinia that includes all 837 reef-building corals (Figures 1, 2, 3). Despite the vast increase in taxon sampling over previous phylogenies [42], [82], the present analysis recovers a highly similar topology. In particular, all 21 clades recognised by Fukami et al. [42] (labelled I to XXI) are present in the supertree.

**Figure 4:**
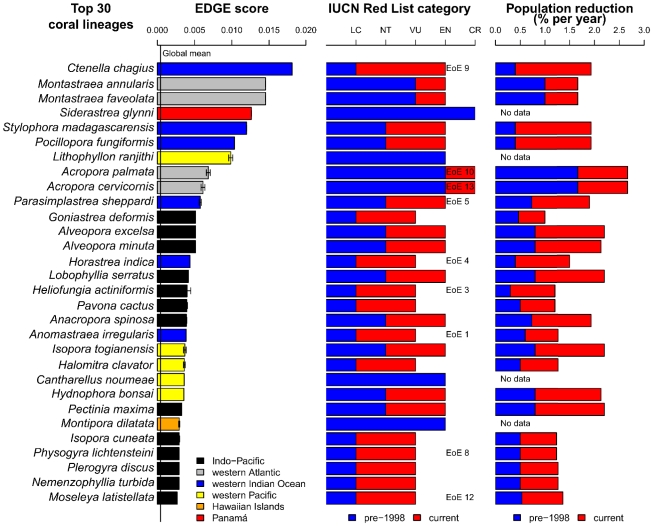
Top 30 reef corals ranked according to EDGE scores. List of corals representing high evolutionary distinctiveness and extinction risk. Left panel shows the EDGE score for each species. Global mean score for all 837 reef corals denoted by vertical line through bars, which are coloured to indicate respective geographic ranges. Error bars represent standard deviation. Middle panel shows pre-1998 and present IUCN Red List categories, as well as ranks according to the EDGE of Existence (EoE) programme. Right panel shows pre-1998 and present rates of global population reduction. IUCN Red List and population reduction data derived from Carpenter et al. [11].

The analysis of EDGE scores has produced a priority list of reef-building corals that are both phylogenetically unique and facing elevated extinction risk (Figure 4; for full ranking, see Table S1). Conservation of these endangered lineages is critical for the preservation of evolutionary diversity. The priority scores of the top 30 species exceed the mean of all reef corals by at least an order of magnitude, and a significantly greater than random loss of phylogenetic diversity would occur should these species go extinct (P<0.001).

**Figure 5:**
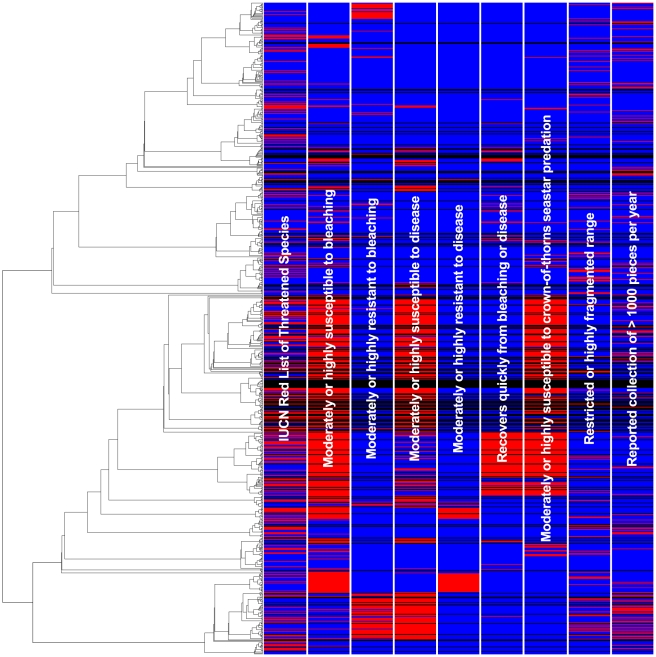
Cladogram of reef corals illustrating phylogenetic signal of traits. This tree represents the first of 1000 random resolutions of the strict consensus supertree. Vertical bars illustrate, in red, degrees of clumping among species classified as Vulnerable (VU) and above, susceptible and/or resistant to specific threats, and those recovering quickly from bleaching and disease. Taxa absent for the above traits are in blue. Data Deficient (DD) species, which are not phylogenetically clumped, are in black.

**Figure 6:**
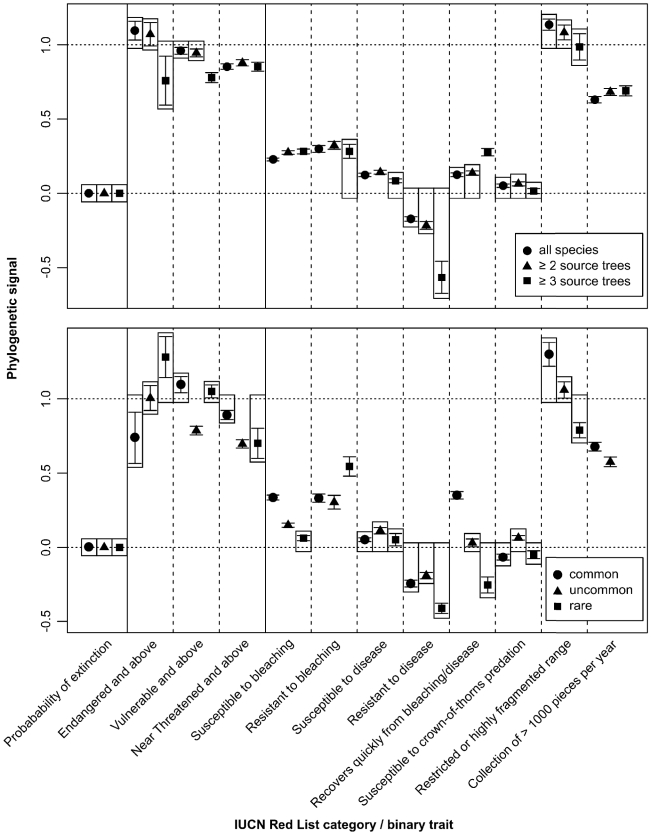
Species' source tree representation and abundances show limited effect on phylogenetic signal strength. Measure of phylogenetic signal based on K for probability of extinction (K = 0 for random continuous traits) and D for all other traits (D = 0 for clumped and D = 1 for random binary traits). Upper and lower panels show levels of phylogenetic signal for datasets with varying degrees of source tree representation and abundance respectively. Error bars represent standard deviation. Means not significantly different from zero or one are enclosed by boxes with those values.

Extinction probability of corals exhibits negligible phylogenetic signal since the hypothesis that there is no signal cannot be rejected given the data, i.e. non-zero K values are only non-zero by chance (P = 0.745, K = 1.584×10^−11^). Threatened species (EN and above, and VU and above) are randomly distributed on the phylogeny, while species given a minimum status of NT are only slightly more clumped than random (Figure 5, Table 2). The datasets comprising species with increased source tree sampling and fixed abundances show very similar patterns, indicating that these factors have limited influence on phylogenetic signal strength (Figure 6). Gains in statistical significance (more clumped than random) are recorded for VU and above corals that are present in ≥3 source trees, as well as for taxa considered at least VU and NT for the uncommon species, but values of D remain close to one (random). Simulated extinction scenarios of reef corals based solely on threat status result in smaller than random losses of PD (P<0.001, EN and above, VU and above, NT and above, all significantly less than random loss).

The tests for phylogenetic signal show that species susceptible to bleaching, disease, and predation by *Acanthaster planci*, as well as those resistant to and recovering quickly from bleaching and disease (i.e. resilient [110]) are at least moderately clumped on the coral tree (Figure 5, Table 2; see [105]). Species' source tree representation and abundances have negligible effects on these inferences (Figure 6). In fact, phylogenetic signal increases among taxa represented by at least three source trees for the traits ‘resistant to bleaching’, ‘susceptible to disease’ and ‘resistant to disease’. It should be noted that in the dataset comprising only rare corals, species resistant to bleaching display relatively low signal (D = 0.545±SD 0.065), but are still significantly more clustered than random on the phylogeny (P = 0.016).

Among lineages, correlations are evident between susceptibilities to bleaching events and disease (P = 0.001), as well as susceptibilities to bleaching and predation (P<0.001). Negative linear relationships are present between susceptibility and resistance for both bleaching (P<0.001) and disease (P<0.001), although there is a positive correlation between susceptibility to disease and quick recovery from bleaching/disease (P = 0.025).

## Discussion

Using the most comprehensive coral phylogeny to date, this study has quantified the expected loss of evolutionary history for reef species based on the EDGE (evolutionarily distinct and globally endangered) measure. The ranking provided here, the first of its kind for corals, has been successful in identifying distinct lineages that warrant the highest conservation attention.

The top-30 list captures three of four CR species and 16 of the 23 EN species, the majority of which have restricted ranges (Figure 4). In particular, the most endangered lineage represented by *Ctenella chagius* is known only from the Chagos Archipelago, Mauritius and La Réunion, while *Siderastrea glynni*, fourth on the list, is endemic to Panamá in the tropical eastern Pacific [92]. The remaining 11 species are of VU status and could be accorded lower conservation priority based upon extinction risk alone. Five of these, *Horastrea indica*, *Heliofungia actiniformis*, *Anomastraea irregularis*, *Physogyra lichtensteini* and *Moseleya latistellata* have only recently been highlighted by the EDGE of Existence programme (http://www.edgeofexistence.org/coral_reef) that aims to identify evolutionarily distinct and globally endangered species. Yet it has failed to recognise 21 of the 30 corals shown here to be of top priority; neither the ‘Isaac’ nor ‘Pessimistic’ transformation increases its representation of high EDGE-scoring species (22 and 24 species overlooked respectively). The programme's methodology remains unknown, but likely utilisation of an incomplete phylogeny may have precluded a comprehensive listing (see also materials and methods in [29]).

Distinctiveness metrics such as ED often account for a greater proportion of total PD than expected [111]. Recent evidence also suggests that evolutionarily distinct species and high PD represent a broader distribution of ecological diversity and higher ecosystem function than expected [112]–[117] (but see [109]). If the preservation of biological diversity is a goal of reef conservation, then such phylogenetically-informed rankings would shore up priority setting efforts that currently focus on species richness, rarity and connectivity [13], [118]–[121].

Despite the heightened risk in a larger fraction of corals relative to birds and mammals [11], groups that exhibit phylogenetic clustering of threat status [105], [122], extinction probability and threatened species of corals show negligible signal associated with phylogeny (Figure 5). That species facing elevated extinction risk are not concentrated in particular parts of the phylogeny is no cause for optimism, however, as recent simulations have shown that other factors are involved in determining the magnitude of PD loss during extinctions [123]. In particular, trees derived from real data generally have asymmetric topologies [124]–[128]; the coral supertree is no exception (P<0.001, Colless' [129] index significantly greater than predicted by the Yule model). Under this circumstance, even random exterminations of species can lead to disproportionate losses of PD [34], [123], [130]. High average extinction probability among reef corals [11] may also exacerbate this pattern [123]. Indeed, random extinction scenarios of coral species lead to larger declines in PD compared to extinctions based on IUCN Red List threat status. In other words, while none of the major clades of reef corals are in immediate danger of complete obliteration, the unbalanced phylogeny and high mean extinction risk suggest that any extinction event can substantially reduce overall PD.

Bleaching, disease, and predation by *A. planci* are three of the most serious stressors affecting coral health today [131], [132]. Tests for phylogenetic signal show that species susceptible to these threats, as well as those resistant and resilient to bleaching and disease are clustered on the tree, indicating that the aggravation of these risk factors can result in disproportionately large PD declines. More worrying is the finding that lineages vulnerable to bleaching events are also more likely to be susceptible to disease and predation. These threats often impact similar sets of species [11], [133]–[136], yet this relationship holds even after controlling for effects of shared common ancestry.

The value of investigating extinction risk in the phylogenetic context has been emphasised in considerable detail elsewhere [26], [29], [32], [38], [101], [137], [138]. Specifically for corals, confusion surrounding traditional taxonomy makes it difficult to accurately generalise traits exhibited by species to higher level taxa [42]. For instance, following the massive bleaching event in 1998, the family Faviidae, including *Leptastrea purpurea* and *L. transversa*, has been declared a ‘winner’ in the recovery process at Sesoko Island, Japan [39], [40]. Yet phylogenies inferred in the last 15 years have unequivocally demonstrated that *Leptastrea* is more closely related to members of Fungiidae rather than Faviidae [42], [52], [53], [82], [139] (see also [140], [141]), recovered within clade X with corals that are resistant to or recover quickly from bleaching (Figures 3, 5). Results here suggest that these traits are conserved on the evolutionary tree, irrespective of species' taxonomic affiliations.

Vulnerabilities of reef corals to bleaching and disease appear to be mediated by the same physiological mechanisms, and immune responses against these threats tend to be similar among close relatives, with Acroporidae and *Porites* (Poritidae) possessing the lowest and highest immunity levels respectively [142]. Consequently, the enhanced susceptibility of *Alveopora* to bleaching [11] is better understood in the context of recent phylogenies that show the genus being placed within Acroporidae (clade VI) rather than, traditionally, Poritidae (clade III) [42], [82]. It is clear that, conventional taxonomy notwithstanding, close relatives are likely to share similar levels of susceptibility, resistance and resilience to various risk factors, underscoring the utility of phylogenetic approaches in understanding specific responses of corals to environmental perturbations.

Subsequent analyses will utilise these results in distinguishing reef regions that make the greatest contribution to evolutionary history, in comparison to the most species-rich areas [143]. A biogeographically-weighted evolutionary distinctiveness (ED) metric has the potential for regional prioritisation [144], but a probabilistic approach that accounts for future extinctions of related species may be more suitable than the static allocation of conservation value afforded by the ED measure [32], [145], [146].

Analyses demonstrating phylogenetic clustering of susceptibilities, resistance and resilience to various risk factors rely on accurate and precise species-specific data. The conservation status of Data Deficient species clearly needs to be assessed while regular updates are necessary for all corals [147], [148]. Increasingly, recent research is revealing a wider range of species responses to environmental threats than before [149]–[152]. Given that these threats exhibit considerable phylogenetic signal, the coral tree of life will prove an excellent framework for investigating these variabilities.

## Supporting Information

Table S1 **Reef and non-reef coral species included in the phylogenetic analysis of Scleractinia.** For each species, the IUCN Red List category and ranks according to the EDGE of Existence (EoE) programme and the present study are shown where appropriate. Species not assessed are indicated as N/A. GenBank accession numbers are provided for DNA sequences (see text for names of markers).
(PDF)
