# Supplementary material for: Threatened Reef Corals of the World
Source: PLoS One. 2012 Mar 30;7(3):e34459. doi: 10.1371/journal.pone.0034459 (PMC3316686; doi:10.1371/journal.pone.0034459)
Supplement: Table S1 — Reef and non-reef coral species included in the phylogenetic analysis of Scleractinia. For each species, the IUCN Red List category and ranks according to the EDGE of Existence (EoE) programme and the present study are shown where appropriate. Species not assessed are indicated as N/A. GenBank accession numbers are provided for DNA sequences (see text for names of markers). (PDF) [file pone.0034459.s001.pdf]

Threatened Reef Corals of the World

Danwei Huang

Table S1. Reef and non-reef coral species included in the phylogenetic analysis of Scleractinia.

Family	Species	Reef	Red List	EoE rank	EDGE rank (IUCN100)	EDGE rank (Isaac)	EDGE rank (Pessimistic)	Molecular sources	Morphological sources	Remarks
Acroporidae	*Acropora abrolhosensis*	Yes	VU	N/A	157	310	309	ND5: EU533959	[69]	
Acroporidae	*Acropora abrotanoides*	Yes	LC	N/A	793	725	725	CTR: FJ899068	[69]	
Acroporidae	*Acropora aculeus*	Yes	VU	N/A	189	427	419		[69]	
Acroporidae	*Acropora acuminata*	Yes	VU	N/A	234	527	527	ND5: EU533969	[69]	
Acroporidae	*Acropora akajimensis*	Yes	DD	N/A	459	553	553		[69]	Junior synonym of *A. donei* [69]
Acroporidae	*Acropora anthocercis*	Yes	VU	N/A	231	523	523	ND5: EU533970	[69]	
Acroporidae	*Acropora appressa*	Yes	NT	N/A	528	723	723		[69]	
Acroporidae	*Acropora arabensis*	Yes	NT	N/A	449	606	606		[69]	Sister species to *A. valida* [69]
Acroporidae	*Acropora aspera*	Yes	VU	N/A	82	116	107	CTR: EU918267; CYB: FJ391987	[69]	
Acroporidae	*Acropora austera*	Yes	NT	N/A	266	158	147	CTR: EU918228; CYB: FJ391989	[69]	
Acroporidae	*Acropora awi*	Yes	VU	N/A	256	567	567		[69]	
Acroporidae	*Acropora batunai*	Yes	VU	N/A	85	134	123	CTR: EU918250; ND5: EU533971	[69]	
Acroporidae	*Acropora bifurcata*	Yes	DD	N/A	579	726	726		[69]	Junior synonym of *A. hyacinthus* [69]
Acroporidae	*Acropora branchi*	Yes	DD	N/A	622	762	762		[69]	
Acroporidae	*Acropora bushyensis*	Yes	LC	N/A	788	708	708	ND5: EU533975	[69]	
Acroporidae	*Acropora cardenae*	Yes	DD	N/A	467	582	582		[69]	
Acroporidae	*Acropora carduus*	Yes	NT	N/A	416	531	531	ND5: EU533976	[69]	
Acroporidae	*Acropora caroliniana*	Yes	VU	N/A	61	74	69	CTR: EU918274	[69]	
Acroporidae	*Acropora cerealis*	Yes	LC	N/A	796	729	729	CTR: EU918248; CYB: AF099652; ND5: EU533979	[69]	
Acroporidae	*Acropora cervicornis*	Yes	CR	13	9	28	174	12S: EF597094; COI: AY451340; CTR: EU918257; CYB: AF099654; ND5: EU533960	[69]	
Acroporidae	*Acropora chesterfieldensis*	Yes	LC	N/A	602	428	420	CTR: EU918262; ND5: EU533981	[69]	
Acroporidae	*Acropora clathrata*	Yes	LC	N/A	819	816	816		[69]	
Acroporidae	*Acropora convexa*	Yes	DD	N/A	622	762	762		[69]	Junior synonym of *A. millepora* [69]
Acroporidae	*Acropora cophodactyla*	Yes	DD	N/A	601	749	749		[69]	Junior synonym of *A. humilis* [69]
Acroporidae	*Acropora copiosa*	Yes	DD	N/A	622	762	762		[69]	Junior synonym of *A. muricata* [69]
Acroporidae	*Acropora cytherea*	Yes	LC	N/A	711	584	584	12S: AF333054; 16S: L75995; CTR: AY083876; CYB: FJ391995	[69]	
Acroporidae	*Acropora dendrum*	Yes	VU	N/A	230	522	522	ND5: EU533983	[69]	Sister species to *A. loisetteae* [69]
Acroporidae	*Acropora derawanensis*	Yes	VU	N/A	35	31	25	CTR: EU918263; ND5: EU533984	[69]	
Acroporidae	*Acropora desalwii*	Yes	VU	N/A	153	292	291		[69]	
Acroporidae	*Acropora digitifera*	Yes	NT	N/A	335	363	355	12S: AF333051; AT6: AB033199; CTR: EU918261; CYB: AB033184	[69]	
Acroporidae	*Acropora divaricata*	Yes	NT	N/A	377	440	432	CTR: AY026432; ND5: EU533985	[69]	
Acroporidae	*Acropora donei*	Yes	VU	N/A	77	96	90	AT6: AB033195; CYB: AB033180	[69]	
Acroporidae	*Acropora downingi*	Yes	LC	N/A	746	637	637		[69]	Sister species to *A. polystoma* [69]
Acroporidae	*Acropora echinata*	Yes	VU	N/A	39	39	34	CYB: FJ391985; ND5: EU533986	[69]	
Acroporidae	*Acropora efflorescens*	Yes	DD	N/A	595	739	739		[69]	Junior synonym of *A. cytherea* [69]
Acroporidae	*Acropora elegans*	Yes	VU	N/A	232	524	524	ND5: EU533990	[69]	
Acroporidae	*Acropora elegantula*	Yes	DD	N/A	622	762	762		[69]	
Acroporidae	*Acropora elseyi*	Yes	LC	N/A	766	669	669	ND5: EU533991	[69]	
Acroporidae	*Acropora exquisita*	Yes	DD	N/A	622	762	762		[69]	
Acroporidae	*Acropora fastigata*	Yes	DD	N/A	622	762	762		[69]	
Acroporidae	*Acropora fenneri*	Yes	DD	N/A	622	762	762		[69]	
Acroporidae	*Acropora filiformis*	Yes	DD	N/A	622	762	762		[69]	
Acroporidae	*Acropora florida*	Yes	NT	N/A	440	599	599	AT6: AB033197; CTR: AY026435; CYB: AB033182; ND5: EU533993	[69]	
Acroporidae	*Acropora forskali*	Yes	DD	N/A	622	762	762		[69]	
Acroporidae	*Acropora gemmifera*	Yes	LC	N/A	786	705	705	AT6: AB033198; CTR: EU918277; CYB: AB033183	[69]	

Family	Species	Reef	Red List	EoE rank	EDGE rank (IUCN100)	EDGE rank (Isaac)	EDGE rank (Pessimistic)	Molecular sources	Morphological sources	Remarks
Acroporidae	*Acropora glauca*	Yes	NT	N/A	528	723	723		[69]	
Acroporidae	*Acropora globiceps*	Yes	VU	N/A	190	429	421	CTR: EF206433	[69]	Sister species to *A. humilis* [69]
Acroporidae	*Acropora gomezi*	Yes	DD	N/A	622	762	762		[69]	
Acroporidae	*Acropora grandis*	Yes	LC	N/A	809	804	804	ND5: EU533994	[69]	
Acroporidae	*Acropora granulosa*	Yes	NT	N/A	416	531	531	ND5: EU533995	[69]	
Acroporidae	*Acropora haimei*	Yes	DD	N/A	622	762	762		[69]	
Acroporidae	*Acropora halmaherae*	Yes	DD	N/A	622	762	762		[69]	
Acroporidae	*Acropora hemprichii*	Yes	VU	N/A	80	101	94	16S: AF550359	[69]	Sister species to *A. austera* [69]
Acroporidae	*Acropora hoeksemai*	Yes	VU	N/A	236	534	534		[69]	
Acroporidae	*Acropora horrida*	Yes	VU	N/A	99	169	161	ND5: EU533998	[69]	
Acroporidae	*Acropora humilis*	Yes	NT	N/A	437	586	586	16S: L75996; CTR: EU918282; CYB: EF363316	[69]	
Acroporidae	*Acropora hyacinthus*	Yes	NT	N/A	396	479	473	12S: AF333053; CTR: AY083877; CYB: FJ391988; ND5: EU534002	[69]	
Acroporidae	*Acropora indonesia*	Yes	VU	N/A	228	514	514	ND5: EU534003	[69]	
Acroporidae	*Acropora inermis*	Yes	DD	N/A	622	762	762		[69]	Junior synonym of *A. horrida* [69]
Acroporidae	*Acropora insignis*	Yes	DD	N/A	622	762	762		[69]	
Acroporidae	*Acropora intermedia*	Yes	LC	N/A	685	533	533	CTR: AY026451; ND5: EU533965	[69]	Senior synonym of *A. nobilis* [69]
Acroporidae	*Acropora irregularis*	Yes	DD	N/A	553	682	682		[69]	Junior synonym of *A. abrotanoides* [69]
Acroporidae	*Acropora jacquelineae*	Yes	VU	N/A	119	210	204	CTR: EU918284; ND5: EU534012	[69]	
Acroporidae	*Acropora japonica*	Yes	DD	N/A	622	762	762		[69]	
Acroporidae	*Acropora khayranensis*	Yes	DD	N/A	622	762	762		[69]	
Acroporidae	*Acropora kimbeensis*	Yes	VU	N/A	113	191	183	CTR: EU918214	[69]	
Acroporidae	*Acropora kirstyae*	Yes	VU	N/A	164	332	330	CTR: EU918215; ND5: EU534015	[69]	
Acroporidae	*Acropora kosurini*	Yes	VU	N/A	236	534	534		[69]	
Acroporidae	*Acropora lamarcki*	Yes	DD	N/A	622	762	762		[69]	
Acroporidae	*Acropora latistella*	Yes	LC	N/A	610	436	428	CTR: AY026443; CYB: AF099656	[69]	
Acroporidae	*Acropora lianae*	Yes	DD	N/A	622	762	762		[69]	
Acroporidae	*Acropora listeri*	Yes	VU	N/A	192	439	431	ND5: EU533966	[69]	
Acroporidae	*Acropora loisetteae*	Yes	VU	N/A	114	202	195	CTR: EU918222	[69]	
Acroporidae	*Acropora lokani*	Yes	VU	N/A	109	183	175	CTR: EU918270	[69]	
Acroporidae	*Acropora longicyathus*	Yes	LC	N/A	808	803	803	CTR: EU918220; ND5: EU534017	[69]	
Acroporidae	*Acropora loripes*	Yes	NT	N/A	401	510	507	CTR: EU918227; ND5: EU534020	[69]	
Acroporidae	*Acropora lovelli*	Yes	VU	N/A	259	570	570		[69]	
Acroporidae	*Acropora lutkeni*	Yes	NT	N/A	378	449	441	ND5: EU534023	[69]	
Acroporidae	*Acropora macrostoma*	Yes	DD	N/A	622	762	762		[69]	Junior synonym of *A. tenuis* [69]
Acroporidae	*Acropora maryae*	Yes	DD	N/A	622	762	762		[69]	
Acroporidae	*Acropora massawensis*	Yes	DD	N/A	569	709	709		[69]	Junior synonym of *A. polystoma* [69]
Acroporidae	*Acropora microclados*	Yes	VU	N/A	137	237	234	ND5: EU534024	[69]	
Acroporidae	*Acropora microphthalma*	Yes	LC	N/A	759	653	653	CTR: EU918203; CYB: FJ391986; ND5: EU534026	[69]	
Acroporidae	*Acropora millepora*	Yes	NT	N/A	477	656	656	CTR: EU918207; CYB: AF099653; ND5: EU534029	[69]	
Acroporidae	*Acropora minuta*	Yes	DD	N/A	622	762	762		[69]	
Acroporidae	*Acropora mirabilis*	Yes	DD	N/A	622	762	762		[69]	
Acroporidae	*Acropora monticulosa*	Yes	NT	N/A	438	588	588	CTR: EF206487	[69]	
Acroporidae	*Acropora multiacuta*	Yes	VU	N/A	195	446	438	CTR: EF206546	[69]	
Acroporidae	*Acropora muricata*	Yes	NT	N/A	402	511	508	12S: AF177042; CYB: AF099651	[69]	Type species of *Acropora* [69]
Acroporidae	*Acropora nana*	Yes	NT	N/A	393	473	467	ND5: EU534031	[69]	
Acroporidae	*Acropora nasuta*	Yes	NT	N/A	466	650	650	AT6: AB033200; CTR: EU918229; CYB: AB033185; ND5: EU534032	[69]	
Acroporidae	*Acropora natalensis*	Yes	DD	N/A	598	741	741		[69]	Junior synonym of *A. solitaryensis* [69]
Acroporidae	*Acropora navini*	Yes	DD	N/A	622	762	762		[69]	
Acroporidae	*Acropora ocellata*	Yes	DD	N/A	552	670	670		[69]	Senior synonym of *A. lutkeni* [69]
Acroporidae	*Acropora orbicularis*	Yes	DD	N/A	599	742	742		[69]	Junior synonym of *A. clathrata* [69]
Acroporidae	*Acropora pagoensis*	Yes	DD	N/A	622	762	762		[69]	
Acroporidae	*Acropora palmata*	Yes	CR	10	8	21	150	12S: EF597092; COI: AB441246; CTR: AF507217; CYB: AB441331; ND5: EU533962	[69]	
Acroporidae	*Acropora palmerae*	Yes	VU	N/A	188	423	415		[69]	
Acroporidae	*Acropora paniculata*	Yes	VU	N/A	229	518	518		[69]	
Acroporidae	*Acropora papillare*	Yes	VU	N/A	32	27	20	CTR: EU918211	[69]	
Acroporidae	*Acropora paragemmifera*	Yes	DD	N/A	622	762	762		[69]	

Family	Species	Reef	Red List	EoE rank	EDGE rank (IUCN100)	EDGE rank (Isaac)	EDGE rank (Pessimistic)	Molecular sources	Morphological sources	Remarks
Acroporidae	*Acropora parahemprichii*	Yes	DD	N/A	622	762	762		[69]	
Acroporidae	*Acropora parapharaonis*	Yes	DD	N/A	622	762	762		[69]	
Acroporidae	*Acropora parilis*	Yes	DD	N/A	622	762	762		[69]	Junior synonym of *A. horrida* [69]
Acroporidae	*Acropora pectinatus*	Yes	DD	N/A	622	762	762		[69]	
Acroporidae	*Acropora pharaonis*	Yes	VU	N/A	259	570	570		[69]	
Acroporidae	*Acropora pichoni*	Yes	NT	N/A	322	325	323	CTR: EU918206; ND5: EU534033	[69]	Sister species to *A. elegans* [69]
Acroporidae	*Acropora pinguis*	Yes	DD	N/A	553	682	682		[69]	Junior synonym of *A. robusta* [69]
Acroporidae	*Acropora plana*	Yes	DD	N/A	622	762	762		[69]	Junior synonym of *A. tenuis* [69]
Acroporidae	*Acropora plantaginea*	Yes	DD	N/A	622	762	762		[69]	
Acroporidae	*Acropora plumosa*	Yes	VU	N/A	259	570	570		[69]	
Acroporidae	*Acropora polystoma*	Yes	VU	N/A	87	138	128	ND5: EU533964	[69]	
Acroporidae	*Acropora prostrata*	Yes	DD	N/A	622	762	762		[69]	Junior synonym of *A. millepora* [69]
Acroporidae	*Acropora proximalis*	Yes	DD	N/A	622	762	762		[69]	
Acroporidae	*Acropora pruinosa*	Yes	DD	N/A	622	762	762		[69]	
Acroporidae	*Acropora pulchra*	Yes	LC	N/A	760	654	654	CTR: EU918230; ND5: EU533967	[69]	
Acroporidae	*Acropora rambleri*	Yes	DD	N/A	596	740	740		[69]	Junior synonym of *A. speciosa* [69]
Acroporidae	*Acropora retusa*	Yes	VU	N/A	147	280	278	CTR: EF206535	[69]	
Acroporidae	*Acropora ridzwani*	Yes	DD	N/A	622	762	762		[69]	
Acroporidae	*Acropora robusta*	Yes	LC	N/A	789	718	718	CTR: FJ899064	[69]	
Acroporidae	*Acropora rongelapensis*	Yes	DD	N/A	422	447	439	CTR: EU918210	[69]	
Acroporidae	*Acropora rosaria*	Yes	DD	N/A	622	762	762		[69]	
Acroporidae	*Acropora roseni*	Yes	EN	N/A	69	360	511		[69]	
Acroporidae	*Acropora rudis*	Yes	EN	N/A	69	360	511		[69]	
Acroporidae	*Acropora rufus*	Yes	DD	N/A	622	762	762		[69]	
Acroporidae	*Acropora russelli*	Yes	VU	N/A	153	292	291		[69]	
Acroporidae	*Acropora samoensis*	Yes	LC	N/A	762	658	658	CTR: AY364095; CYB: FJ391994	[69]	
Acroporidae	*Acropora sarmentosa*	Yes	LC	N/A	797	737	737	CTR: AY026455; ND5: EU534034	[69]	
Acroporidae	*Acropora scherzeriana*	Yes	DD	N/A	567	706	706		[69]	Junior synonym of *A. gemmifera* [69]
Acroporidae	*Acropora schmitti*	Yes	DD	N/A	512	636	636		[69]	Junior synonym of *A. digitifera* [69]
Acroporidae	*Acropora secale*	Yes	NT	N/A	479	660	660		[69]	
Acroporidae	*Acropora sekiseiensis*	Yes	DD	N/A	622	762	762		[69]	Junior synonym of *A. horrida* [69]
Acroporidae	*Acropora selago*	Yes	NT	N/A	477	656	656	CTR: AB361179; ND5: EU534035	[69]	
Acroporidae	*Acropora seriata*	Yes	DD	N/A	622	762	762		[69]	
Acroporidae	*Acropora simplex*	Yes	VU	N/A	194	442	434		[69]	
Acroporidae	*Acropora solitaryensis*	Yes	VU	N/A	233	525	525	ND5: EU534039	[69]	
Acroporidae	*Acropora spathulata*	Yes	LC	N/A	798	738	738	CTR: EU918209; ND5: EU534040	[69]	
Acroporidae	*Acropora speciosa*	Yes	VU	N/A	37	35	29	CTR: EU918245	[69]	
Acroporidae	*Acropora spicifera*	Yes	VU	N/A	175	398	390	CTR: AY083881; ND5: EU534041	[69]	
Acroporidae	*Acropora squarrosa*	Yes	LC	N/A	767	685	685		[69]	Sister species to *A. loripes* [69]
Acroporidae	*Acropora stoddarti*	Yes	DD	N/A	603	753	753		[69]	Junior synonym of *A. divaricata* [69]
Acroporidae	*Acropora striata*	Yes	VU	N/A	259	570	570		[69]	
Acroporidae	*Acropora subglabra*	Yes	LC	N/A	834	833	833	ND5: EU534042	[69]	
Acroporidae	*Acropora subulata*	Yes	LC	N/A	763	659	659		[69]	
Acroporidae	*Acropora suharsonoi*	Yes	EN	N/A	68	359	510		[69]	
Acroporidae	*Acropora sukarnoi*	Yes	DD	N/A	471	587	587		[69]	
Acroporidae	*Acropora tanegashimensis*	Yes	DD	N/A	564	686	686		[69]	Sister species to *A. hyacinthus* [69]
Acroporidae	*Acropora tenella*	Yes	VU	N/A	97	167	159	CTR: EU918240	[69]	
Acroporidae	*Acropora tenuis*	Yes	NT	N/A	297	250	247	12S: AF338425; 16S: AF338425; AT6: AF338425; COI: AF338425; CTR: AF338425; CYB: AF338425; ND5: AF338425	[69]	
Acroporidae	*Acropora teres*	Yes	DD	N/A	622	762	762		[69]	
Acroporidae	*Acropora tizardi*	Yes	DD	N/A	603	753	753		[69]	Junior synonym of *A. cerealis* [69]
Acroporidae	*Acropora torihalimeda*	Yes	DD	N/A	467	582	582		[69]	
Acroporidae	*Acropora torresiana*	Yes	DD	N/A	622	762	762		[69]	
Acroporidae	*Acropora tortuosa*	Yes	LC	N/A	545	324	322	CTR: EU918238	[69]	
Acroporidae	*Acropora tumida*	Yes	DD	N/A	603	753	753		[69]	Junior synonym of *A. valida* [69]
Acroporidae	*Acropora turaki*	Yes	VU	N/A	256	567	567		[69]	
Acroporidae	*Acropora tutuilensis*	Yes	DD	N/A	553	682	682		[69]	Junior synonym of *A. abrotanoides* [69]
Acroporidae	*Acropora valenciennesi*	Yes	LC	N/A	835	834	834		[69]	
Acroporidae	*Acropora valida*	Yes	LC	N/A	747	638	638	CTR: EU918235; CYB: AF099658; ND5: EU534047	[69]	

Family	Species	Reef	Red List	EoE rank	EDGE rank (IUCN100)	EDGE rank (Isaac)	EDGE rank (Pessimistic)	Molecular sources	Morphological sources	Remarks
Acroporidae	*Acropora variabilis*	Yes	DD	N/A	603	753	753		[69]	Junior synonym of *A. valida* [69]
Acroporidae	*Acropora variolosa*	Yes	LC	N/A	835	834	834		[69]	Sister species to *A. rudis* [69]
Acroporidae	*Acropora vaughani*	Yes	VU	N/A	83	125	115	CTR: EU918224	[69]	
Acroporidae	*Acropora vermiculata*	Yes	DD	N/A	575	722	722		[69]	Junior synonym of *A. sarmentosa* [69]
Acroporidae	*Acropora verweyi*	Yes	VU	N/A	259	570	570		[69]	
Acroporidae	*Acropora walindii*	Yes	VU	N/A	75	90	84	CTR: EU918234	[69]	Sister species to *A. tenella* [69]
Acroporidae	*Acropora wallaceae*	Yes	DD	N/A	551	668	668		[69]	Junior synonym of *A. samoensis* [69]
Acroporidae	*Acropora willisae*	Yes	VU	N/A	256	567	567		[69]	
Acroporidae	*Acropora yongei*	Yes	LC	N/A	818	815	815	ND5: EU534048	[69]	
Acroporidae	*Anacropora forbesi*	Yes	LC	N/A	461	196	188	COI: AB441251; CYB: AB441336	[69]	
Acroporidae	*Anacropora matthai*	Yes	VU	N/A	59	72	67	12S: AY903295; 16S: AY903295; AT6: AY903295; COI: AY903295; CTR: AY903295; CYB: AY903295; ND5: AY903295	[69]	
Acroporidae	*Anacropora pillai*	Yes	DD	N/A	447	488	482		[69]	
Acroporidae	*Anacropora puertogalerae*	Yes	VU	N/A	138	243	240		[69]	
Acroporidae	*Anacropora reticulata*	Yes	VU	N/A	138	243	240		[69]	
Acroporidae	*Anacropora spinosa*	Yes	EN	N/A	18	98	198		[69]	
Acroporidae	*Anacropora spumosa*	Yes	DD	N/A	447	488	482		[69]	
Acroporidae	*Astreopora acroporina*	Yes	DD	N/A	541	663	663		[69]	New species [47]
Acroporidae	*Astreopora cenderawasih*	Yes	DD	N/A	541	663	663		[69]	New species [47]
Acroporidae	*Astreopora cucullata*	Yes	VU	N/A	176	399	391		[69]	
Acroporidae	*Astreopora eliptica*	Yes	DD	N/A	541	663	663		[69]	
Acroporidae	*Astreopora expansa*	Yes	NT	N/A	450	613	613		[69]	
Acroporidae	*Astreopora gracilis*	Yes	LC	N/A	799	743	743		[69]	
Acroporidae	*Astreopora incrustans*	Yes	VU	N/A	176	399	391		[69]	
Acroporidae	*Astreopora listeri*	Yes	LC	N/A	799	743	743		[69]	
Acroporidae	*Astreopora macrostoma*	Yes	NT	N/A	450	613	613		[69]	
Acroporidae	*Astreopora montiporina*	Yes	DD	N/A	541	663	663		[69]	New species [47]
Acroporidae	*Astreopora moretonensis*	Yes	VU	N/A	176	399	391		[69]	
Acroporidae	*Astreopora myriophthalma*	Yes	LC	N/A	446	164	156	12S: AF177046; AT6: AB033186; COI: AB441253; CYB: AB441338	[69]	
Acroporidae	*Astreopora ocellata*	Yes	LC	N/A	799	743	743		[69]	
Acroporidae	*Astreopora randalli*	Yes	LC	N/A	799	743	743		[69]	
Acroporidae	*Astreopora scabra*	Yes	LC	N/A	799	743	743		[69]	
Acroporidae	*Astreopora suggesta*	Yes	LC	N/A	799	743	743		[69]	
Acroporidae	*Enigmopora darveliensis*	Yes	DD	N/A	682	806	806			Closest to *Acropora* (prior to elevation of *Isopora*) [77]
Acroporidae	*Isopora brueggemanni*	Yes	VU	N/A	93	151	141	12S: AF333048; AT6: AB033193; COI: AB441247; CYB: AB441332; ND5: EU534004	[69]	
Acroporidae	*Isopora crateriformis*	Yes	VU	N/A	169	369	361		[69]	
Acroporidae	*Isopora cuneata*	Yes	VU	N/A	26	16	11	12S: AF333049; CTR: AY026429; ND5: EU534006	[69]	
Acroporidae	*Isopora cylindrica*	Yes	DD	N/A	514	639	639		[69]	
Acroporidae	*Isopora elizabethensis*	Yes	DD	N/A	514	639	639		[69]	
Acroporidae	*Isopora meridiana*	Yes	DD	N/A	514	639	639		[69]	Junior synonym of *Acropora brueggemanni* [69]
Acroporidae	*Isopora palifera*	Yes	NT	N/A	309	294	293	12S: AF177044; 16S: AF265593; AT6: AB033194; COI: AB441248; CYB: AB441333; ND5: EU534010	[69]	
Acroporidae	*Isopora togianensis*	Yes	EN	N/A	20	107	214	12S: AF333050; COI: AB441249; CYB: AB441334; ND5: EU534008	[69]	Morphological coding updated [94]
Acroporidae	*Montipora aequituberculata*	Yes	LC	N/A	726	610	610	12S: AF333045; AT6: AB033187; CTR: AY313548; CYB: AB033172	[69]	
Acroporidae	*Montipora altasepta*	Yes	VU	N/A	58	70	65	AT6: AB033190; CTR: AY313572; CYB: AB033175	[69]	
Acroporidae	*Montipora angulata*	Yes	VU	N/A	74	89	83	CTR: AY313563	[69]	
Acroporidae	*Montipora aspergillus*	Yes	DD	N/A	587	730	730		[69]	
Acroporidae	*Montipora australiensis*	Yes	VU	N/A	204	491	488		[69]	

Family	Species	Reef	Red List	EoE rank	EDGE rank (IUCN100)	EDGE rank (Isaac)	EDGE rank (Pessimistic)	Molecular sources	Morphological sources	Remarks
Acroporidae	*Montipora cactus*	Yes	VU	N/A	90	145	135	12S: AY903296; 16S: AY903296; AT6: AY903296; COI: AY903296; CTR: AY903296; CYB: AY903296; ND5: AY903296	[69]	
Acroporidae	*Montipora calcarea*	Yes	VU	N/A	204	491	488		[69]	
Acroporidae	*Montipora caliculata*	Yes	VU	N/A	204	491	488		[69]	
Acroporidae	*Montipora capitata*	Yes	NT	N/A	338	367	359	16S: HQ246709; AT6: HQ246686; COI: HQ246613; CTR: HQ246520; CYB: HQ246516	[69]	
Acroporidae	*Montipora capricornis*	Yes	VU	N/A	76	94	88	CTR: AY313583	[69]	
Acroporidae	*Montipora cebuensis*	Yes	VU	N/A	204	491	488		[69]	
Acroporidae	*Montipora circumvallata*	Yes	LC	N/A	276	13	9	16S: AF550368	[69]	
Acroporidae	*Montipora cocosensis*	Yes	VU	N/A	204	491	488		[69]	
Acroporidae	*Montipora confusa*	Yes	NT	N/A	300	265	263	CTR: AY313551	[69]	
Acroporidae	*Montipora corbettensis*	Yes	VU	N/A	204	491	488		[69]	
Acroporidae	*Montipora crassituberculata*	Yes	VU	N/A	204	491	488		[69]	
Acroporidae	*Montipora cryptus*	Yes	NT	N/A	489	671	671		[69]	
Acroporidae	*Montipora danae*	Yes	LC	N/A	609	435	427	CTR: AY313549	[69]	
Acroporidae	*Montipora delicatula*	Yes	VU	N/A	118	209	203	CTR: AY313566	[69]	
Acroporidae	*Montipora digitata*	Yes	LC	N/A	768	687	687	12S: AF177045; 16S: L75993; AT6: AB033188; CTR: AY313579; CYB: AB033173	[69]	
Acroporidae	*Montipora dilatata*	Yes	EN	N/A	25	156	282	16S: HQ246702; AT6: HQ246678; COI: HQ246605; CTR: HQ246554; CYB: HQ246508	[69]	
Acroporidae	*Montipora echinata*	Yes	DD	N/A	587	730	730		[69]	
Acroporidae	*Montipora efflorescens*	Yes	NT	N/A	253	136	126	AT6: AB033189; CYB: AB033174	[69]	
Acroporidae	*Montipora effusa*	Yes	NT	N/A	489	671	671		[69]	
Acroporidae	*Montipora flabellata*	Yes	VU	N/A	155	299	298	16S: HQ246698; AT6: HQ246674; COI: HQ246601; CTR: HQ246561; CYB: HQ246504	[69]	
Acroporidae	*Montipora florida*	Yes	VU	N/A	81	105	98	CTR: AY313562	[69]	
Acroporidae	*Montipora floweri*	Yes	LC	N/A	810	807	807		[69]	
Acroporidae	*Montipora foliosa*	Yes	NT	N/A	239	133	122	CYB: FJ392003	[69]	
Acroporidae	*Montipora foveolata*	Yes	NT	N/A	489	671	671		[69]	
Acroporidae	*Montipora friabilis*	Yes	VU	N/A	204	491	488		[69]	
Acroporidae	*Montipora gaimardi*	Yes	VU	N/A	72	87	81	CTR: AY313565	[69]	
Acroporidae	*Montipora grisea*	Yes	LC	N/A	810	807	807		[69]	
Acroporidae	*Montipora hemispherica*	Yes	DD	N/A	587	730	730		[69]	
Acroporidae	*Montipora hirsuta*	Yes	NT	N/A	489	671	671		[69]	
Acroporidae	*Montipora hispida*	Yes	LC	N/A	663	454	446	CTR: AY313553; CYB: FJ392005	[69]	
Acroporidae	*Montipora hodgsoni*	Yes	VU	N/A	204	491	488		[69]	
Acroporidae	*Montipora hoffmeisteri*	Yes	LC	N/A	770	689	689	CTR: AY313580	[69]	
Acroporidae	*Montipora incrassata*	Yes	NT	N/A	324	330	328	16S: HQ246710; AT6: HQ246687; COI: HQ246614; CTR: HQ246593; CYB: HQ246517	[69]	*Montipora* cf. *incrassata* in GenBank
Acroporidae	*Montipora informis*	Yes	LC	N/A	810	807	807		[69]	
Acroporidae	*Montipora kellyi*	Yes	DD	N/A	587	730	730		[69]	
Acroporidae	*Montipora lobulata*	Yes	VU	N/A	204	491	488		[69]	
Acroporidae	*Montipora mactanensis*	Yes	VU	N/A	204	491	488		[69]	
Acroporidae	*Montipora malampaya*	Yes	VU	N/A	204	491	488		[69]	
Acroporidae	*Montipora meandrina*	Yes	VU	N/A	204	491	488		[69]	
Acroporidae	*Montipora millepora*	Yes	LC	N/A	810	807	807		[69]	
Acroporidae	*Montipora mollis*	Yes	LC	N/A	785	704	704	CTR: AY313552	[69]	
Acroporidae	*Montipora monasteriata*	Yes	LC	N/A	810	807	807		[69]	
Acroporidae	*Montipora niugini*	Yes	NT	N/A	489	671	671		[69]	
Acroporidae	*Montipora nodosa*	Yes	NT	N/A	489	671	671		[69]	
Acroporidae	*Montipora orientalis*	Yes	VU	N/A	204	491	488		[69]	
Acroporidae	*Montipora pachytuberculata*	Yes	DD	N/A	587	730	730		[69]	
Acroporidae	*Montipora palawanensis*	Yes	NT	N/A	489	671	671		[69]	
Acroporidae	*Montipora patula*	Yes	VU	N/A	117	208	202	16S: HQ246691; AT6: HQ246667; COI: HQ246595; CTR: HQ246573; CYB: HQ246497	[69]	

Family	Species	Reef	Red List	EoE rank	EDGE rank (IUCN100)	EDGE rank (Isaac)	EDGE rank (Pessimistic)	Molecular sources	Morphological sources	Remarks
Acroporidae	*Montipora peltiformis*	Yes	NT	N/A	363	420	412	CTR: AY313550	[69]	
Acroporidae	*Montipora porites*	Yes	NT	N/A	489	671	671		[69]	
Acroporidae	*Montipora samarensis*	Yes	VU	N/A	204	491	488		[69]	
Acroporidae	*Montipora saudii*	Yes	NT	N/A	489	671	671		[69]	
Acroporidae	*Montipora setosa*	Yes	EN	N/A	52	311	461		[69]	
Acroporidae	*Montipora spongiosa*	Yes	LC	N/A	810	807	807		[69]	
Acroporidae	*Montipora spongodes*	Yes	LC	N/A	769	688	688	CTR: AY313547	[69]	
Acroporidae	*Montipora spumosa*	Yes	LC	N/A	810	807	807		[69]	
Acroporidae	*Montipora stellata*	Yes	LC	N/A	726	610	610	CTR: AY313587	[69]	
Acroporidae	*Montipora stilosa*	Yes	VU	N/A	204	491	488		[69]	
Acroporidae	*Montipora taiwanensis*	Yes	DD	N/A	587	730	730		[69]	
Acroporidae	*Montipora tuberculosa*	Yes	LC	N/A	810	807	807		[69]	
Acroporidae	*Montipora turgescens*	Yes	LC	N/A	765	662	662	16S: HQ246704; AT6: HQ246681; COI: HQ246608; CTR: HQ246564; CYB: HQ246511	[69]	*Montipora* cf. *turgescens* in GenBank
Acroporidae	*Montipora turtlensis*	Yes	VU	N/A	156	308	307	CTR: AY313574	[69]	
Acroporidae	*Montipora undata*	Yes	NT	N/A	286	221	216	CTR: AY313569	[69]	
Acroporidae	*Montipora vaughani*	Yes	DD	N/A	587	730	730		[69]	
Acroporidae	*Montipora venosa*	Yes	NT	N/A	489	671	671		[69]	
Acroporidae	*Montipora verrilli*	Yes	DD	N/A	436	472	465	16S: HQ246695; AT6: HQ246671; COI: HQ246598; CTR: HQ246582; CYB: HQ246501	[69]	
Acroporidae	*Montipora verrucosa*	Yes	LC	N/A	719	601	601	12S: EF597090; CTR: AY313584	[69]	
Acroporidae	*Montipora verruculosus*	Yes	VU	N/A	204	491	488		[69]	
Acroporidae	*Montipora vietnamensis*	Yes	VU	N/A	204	491	488		[69]	
Agariciidae	*Agaricia agaricites*	Yes	LC	N/A	617	443	435	12S: EF597079; COI: AY451366	[68]	
Agariciidae	*Agaricia fragilis*	Yes	DD	N/A	313	222	217	12S: EF597077	[68]	
Agariciidae	*Agaricia grahamae*	Yes	LC	N/A	684	526	526	12S: EF597078		
Agariciidae	*Agaricia humilis*	Yes	LC	N/A	540	318	316	12S: DQ643831; 16S: DQ643831; AT6: DQ643831; COI: DQ643831; CYB: DQ643831; ND5: DQ643831		
Agariciidae	*Agaricia lamarcki*	Yes	VU	N/A	51	61	56	12S: EF597076; COI: AY451369		
Agariciidae	*Agaricia tenuifolia*	Yes	NT	N/A	323	327	325	12S: EF597081; COI: AY451370		
Agariciidae	*Agaricia undata*	Yes	DD	N/A	397	393	385	12S: EF597075		
Agariciidae	*Coeloseris mayeri*	Yes	LC	ED	674	477	471			Closest to *Pavona* [90]
Agariciidae	*Gardineroseris planulata*	Yes	LC	N/A	556	338	336	12S: EF597084; COI: AB441218; CYB: AB441303		
Agariciidae	*Helioseris cucullata*	Yes	LC	N/A	370	71	66	COI: AB441220; CYB: AB441305	[68]	
Agariciidae	*Leptoseris amitoriensis*	Yes	NT	N/A	353	403	395			*Leptoseris* monophyly assumed; closest to *L. papyracea* [91]
Agariciidae	*Leptoseris cailleti*	Yes	LC	N/A	735	621	621			*Leptoseris* monophyly assumed; closest to *L. papyracea* [92]
Agariciidae	*Leptoseris explanata*	Yes	LC	N/A	735	621	621			*Leptoseris* monophyly assumed
Agariciidae	*Leptoseris foliosa*	Yes	LC	N/A	735	621	621			*Leptoseris* monophyly assumed; closest to *L. mycetoseroides* [90]
Agariciidae	*Leptoseris gardineri*	Yes	LC	N/A	735	621	621			*Leptoseris* monophyly assumed; closest to *L. papyracea* [90]
Agariciidae	*Leptoseris hawaiiensis*	Yes	LC	N/A	735	621	621			*Leptoseris* monophyly assumed; closest to *L. scabra* [90]
Agariciidae	*Leptoseris incrustans*	Yes	VU	N/A	123	223	219	16S: L76012		
Agariciidae	*Leptoseris mycetoseroides*	Yes	LC	N/A	735	621	621			*Leptoseris* monophyly assumed
Agariciidae	*Leptoseris papyracea*	Yes	LC	N/A	735	621	621			*Leptoseris* monophyly assumed
Agariciidae	*Leptoseris scabra*	Yes	LC	N/A	735	621	621			*Leptoseris* monophyly assumed
Agariciidae	*Leptoseris solida*	Yes	LC	N/A	735	621	621			*Leptoseris* monophyly assumed; closest to *L. scabra* [92]
Agariciidae	*Leptoseris striata*	Yes	NT	N/A	353	403	395			*Leptoseris* monophyly assumed; closest to *L. scabra* [93]
Agariciidae	*Leptoseris tubulifera*	Yes	LC	N/A	735	621	621			*Leptoseris* monophyly assumed
Agariciidae	*Leptoseris yabei*	Yes	VU	N/A	129	229	225			*Leptoseris* monophyly assumed; closest to *L. mycetoseroides* [90]
Agariciidae	*Pachyseris foliosa*	Yes	LC	N/A	686	536	536			*Pachyseris* monophyly assumed; closest to *P. involuta* [91]
Agariciidae	*Pachyseris gemmae*	Yes	NT	N/A	278	197	190			*Pachyseris* monophyly assumed; closest to *P. rugosa* & *P. speciosa* [92]
Agariciidae	*Pachyseris involuta*	Yes	VU	N/A	98	168	160			*Pachyseris* monophyly assumed
Agariciidae	*Pachyseris rugosa*	Yes	VU	N/A	57	69	64			*Pachyseris* monophyly assumed
Agariciidae	*Pachyseris speciosa*	Yes	LC	N/A	380	83	78	COI: AB441222; CYB: AB441307		
Agariciidae	*Pavona bipartita*	Yes	VU	N/A	124	224	220		[64]	

Family	Species	Reef	Red List	EoE rank	EDGE rank (IUCN100)	EDGE rank (Isaac)	EDGE rank (Pessimistic)	Molecular sources	Morphological sources	Remarks
Agariciidae	*Pavona cactus*	Yes	VU	N/A	17	10	6	16S: AF550370; COI: AB441217; CYB: AB441302	[64]	
Agariciidae	*Pavona chiriquiensis*	Yes	LC	N/A	730	616	616		[64]	
Agariciidae	*Pavona clavus*	Yes	LC	N/A	570	368	360	12S: DQ643836; 16S: DQ643836; AT6: DQ643836; COI: DQ643836; CYB: DQ643836; ND5: DQ643836	[64]	
Agariciidae	*Pavona danai*	Yes	VU	N/A	124	224	220		[64]	
Agariciidae	*Pavona decussata*	Yes	VU	N/A	124	224	220		[64]	
Agariciidae	*Pavona diffluens*	Yes	VU	N/A	124	224	220		[64]	
Agariciidae	*Pavona duerdeni*	Yes	LC	N/A	730	616	616		[64]	
Agariciidae	*Pavona explanulata*	Yes	LC	N/A	730	616	616		[64]	
Agariciidae	*Pavona frondifera*	Yes	LC	N/A	517	284	283	12S: AF333055	[64]	
Agariciidae	*Pavona gigantea*	Yes	LC	N/A	730	616	616		[64]	
Agariciidae	*Pavona maldivensis*	Yes	LC	N/A	730	616	616		[64]	
Agariciidae	*Pavona minuta*	Yes	NT	N/A	352	402	394		[64]	
Agariciidae	*Pavona varians*	Yes	LC	N/A	669	464	456	12S: EF597083; 16S: L76016	[64]	
Agariciidae	*Pavona venosa*	Yes	VU	N/A	124	224	220		[64]	
Agariciidae	*Pavona xarifae*	Yes	DD	N/A	439	480	474		[64]	
Anthemiphyllidae	*Anthemiphyllia dentata*	No	N/A	N/A	N/A	N/A	N/A	COI: HM018603	[64]	
Anthemiphyllidae	*Anthemiphyllia frustum*	No	N/A	N/A	N/A	N/A	N/A		[64]	
Anthemiphyllidae	*Anthemiphyllia macrolobata*	No	N/A	N/A	N/A	N/A	N/A		[64]	
Anthemiphyllidae	*Anthemiphyllia multidentata*	No	N/A	N/A	N/A	N/A	N/A		[64]	
Anthemiphyllidae	*Anthemiphyllia pacifica*	No	N/A	N/A	N/A	N/A	N/A		[64]	
Anthemiphyllidae	*Anthemiphyllia patera*	No	N/A	N/A	N/A	N/A	N/A	COI: HM018604	[64]	
Anthemiphyllidae	*Anthemiphyllia spinifera*	No	N/A	N/A	N/A	N/A	N/A	16S: AF265596	[64]	
Astrocoeniidae	*Madracis asanoi*	Yes	DD	N/A	252	81	76	COI: HM018656		
Astrocoeniidae	*Madracis asperula*	Yes	DD	N/A	273	113	105			Closest to *M. asanoi* [92]
Astrocoeniidae	*Madracis auretenra*	Yes	LC	N/A	469	207	201	12S: EU400212; 16S: EU400212; AT6: EU400212; COI: EU400212; CYB: EU400212		Probably *M. mirabilis* in GenBank [84]
Astrocoeniidae	*Madracis carmabi*	Yes	DD	N/A	292	166	158	12S: EF596980		
Astrocoeniidae	*Madracis decactis*	Yes	LC	N/A	482	251	248	12S: EF596982	[68]	
Astrocoeniidae	*Madracis formosa*	Yes	LC	N/A	463	200	193	12S: EF596981		
Astrocoeniidae	*Madracis kirbyi*	Yes	LC	N/A	483	252	249			Closest to *M. decactis* [92]
Astrocoeniidae	*Madracis pharensis*	Yes	LC	N/A	462	198	191	12S: EF596983		
Astrocoeniidae	*Madracis senaria*	Yes	LC	N/A	464	201	194	12S: EF596979		
Astrocoeniidae	*Palauastrea ramosa*	Yes	NT	N/A	275	175	166			Closest to *Madracis* [97]
Astrocoeniidae	*Stephanocoenia intersepta*	Yes	LC	ED	321	33	27	12S: EF597072; COI: AB441228; CYB: AB441313	[68]	Senior synonym of *S. michelinii*
Astrocoeniidae	*Stylocoeniella armata*	Yes	LC	N/A	503	264	262			*Stylocoeniella* monophyly assumed; closest to *S. guentheri* [92]
Astrocoeniidae	*Stylocoeniella cocosensis*	Yes	VU	N/A	54	66	61			*Stylocoeniella* monophyly assumed
Astrocoeniidae	*Stylocoeniella guentheri*	Yes	LC	N/A	389	92	86	COI: AB441225; CYB: AB441310		
Astrocoeniidae	*Stylocoeniella muscosus*	Yes	DD	N/A	328	256	254			*Stylocoeniella* monophyly assumed
Caryophylliidae	*Caryophyllia abrupta*	No	N/A	N/A	N/A	N/A	N/A		[64]	
Caryophylliidae	*Caryophyllia abyssorum*	No	N/A	N/A	N/A	N/A	N/A		[64]	
Caryophylliidae	*Caryophyllia alaskensis*	No	N/A	N/A	N/A	N/A	N/A		[64]	
Caryophylliidae	*Caryophyllia alberti*	No	N/A	N/A	N/A	N/A	N/A		[64]	
Caryophylliidae	*Caryophyllia ambrosia*	No	N/A	N/A	N/A	N/A	N/A	16S: AF550362	[64,68]	
Caryophylliidae	*Caryophyllia antarctica*	No	N/A	N/A	N/A	N/A	N/A		[64]	
Caryophylliidae	*Caryophyllia antillarum*	No	N/A	N/A	N/A	N/A	N/A		[64]	
Caryophylliidae	*Caryophyllia arnoldi*	No	N/A	N/A	N/A	N/A	N/A		[64]	
Caryophylliidae	*Caryophyllia aspera*	No	N/A	N/A	N/A	N/A	N/A		[64]	
Caryophylliidae	*Caryophyllia atlantica*	No	N/A	N/A	N/A	N/A	N/A	16S: FJ788113; COI: HM018613	[64]	
Caryophylliidae	*Caryophyllia balanacea*	No	N/A	N/A	N/A	N/A	N/A		[64]	
Caryophylliidae	*Caryophyllia barbadensis*	No	N/A	N/A	N/A	N/A	N/A		[64]	
Caryophylliidae	*Caryophyllia berteriana*	No	N/A	N/A	N/A	N/A	N/A		[64]	
Caryophylliidae	*Caryophyllia calveri*	No	N/A	N/A	N/A	N/A	N/A		[64]	
Caryophylliidae	*Caryophyllia cincticulatus*	No	N/A	N/A	N/A	N/A	N/A		[64]	
Caryophylliidae	*Caryophyllia concreta*	No	N/A	N/A	N/A	N/A	N/A		[64]	

Family	Species	Reef	Red List	EoE rank	EDGE rank (IUCN100)	EDGE rank (Isaac)	EDGE rank (Pessimistic)	Molecular sources	Morphological sources	Remarks
Caryophylliidae	*Caryophyllia cornulum*	No	N/A	N/A	N/A	N/A	N/A		[64]	
Caryophylliidae	*Caryophyllia corona*	No	N/A	N/A	N/A	N/A	N/A		[64]	
Caryophylliidae	*Caryophyllia corrugata*	No	N/A	N/A	N/A	N/A	N/A		[64]	
Caryophylliidae	*Caryophyllia crosnieri*	No	N/A	N/A	N/A	N/A	N/A		[64]	
Caryophylliidae	*Caryophyllia crypta*	No	N/A	N/A	N/A	N/A	N/A		[64]	
Caryophylliidae	*Caryophyllia cyathus*	No	N/A	N/A	N/A	N/A	N/A		[64]	
Caryophylliidae	*Caryophyllia decamera*	No	N/A	N/A	N/A	N/A	N/A		[64]	
Caryophylliidae	*Caryophyllia dentata*	No	N/A	N/A	N/A	N/A	N/A		[64]	
Caryophylliidae	*Caryophyllia diomedeae*	No	N/A	N/A	N/A	N/A	N/A	16S: FJ788115; COI: HM018614	[64]	
Caryophylliidae	*Caryophyllia eltaninae*	No	N/A	N/A	N/A	N/A	N/A		[64]	
Caryophylliidae	*Caryophyllia ephyala*	No	N/A	N/A	N/A	N/A	N/A		[64]	
Caryophylliidae	*Caryophyllia foresti*	No	N/A	N/A	N/A	N/A	N/A		[64]	
Caryophylliidae	*Caryophyllia grandis*	No	N/A	N/A	N/A	N/A	N/A	16S: FJ788117	[64]	
Caryophylliidae	*Caryophyllia grayi*	No	N/A	N/A	N/A	N/A	N/A	16S: FJ788119; COI: HM018615	[64]	
Caryophylliidae	*Caryophyllia hawaiiensis*	No	N/A	N/A	N/A	N/A	N/A		[64]	
Caryophylliidae	*Caryophyllia horologium*	No	N/A	N/A	N/A	N/A	N/A		[64]	
Caryophylliidae	*Caryophyllia huinayensis*	No	N/A	N/A	N/A	N/A	N/A		[64]	
Caryophylliidae	*Caryophyllia inornata*	No	N/A	N/A	N/A	N/A	N/A	12S: EF597042; 16S: AF265599	[64]	
Caryophylliidae	*Caryophyllia japonica*	No	N/A	N/A	N/A	N/A	N/A		[64]	
Caryophylliidae	*Caryophyllia jogashimaensis*	No	N/A	N/A	N/A	N/A	N/A		[64]	
Caryophylliidae	*Caryophyllia karubarica*	No	N/A	N/A	N/A	N/A	N/A		[64]	
Caryophylliidae	*Caryophyllia laevigata*	No	N/A	N/A	N/A	N/A	N/A		[64]	
Caryophylliidae	*Caryophyllia lamellifera*	No	N/A	N/A	N/A	N/A	N/A	16S: FJ788120; COI: HM018616	[64]	
Caryophylliidae	*Caryophyllia mabahithi*	No	N/A	N/A	N/A	N/A	N/A		[64]	
Caryophylliidae	*Caryophyllia marmorea*	No	N/A	N/A	N/A	N/A	N/A		[64]	
Caryophylliidae	*Caryophyllia oblonga*	No	N/A	N/A	N/A	N/A	N/A		[64]	
Caryophylliidae	*Caryophyllia octonaria*	No	N/A	N/A	N/A	N/A	N/A		[64]	
Caryophylliidae	*Caryophyllia octopali*	No	N/A	N/A	N/A	N/A	N/A		[64]	
Caryophylliidae	*Caryophyllia paradoxus*	No	N/A	N/A	N/A	N/A	N/A		[64]	
Caryophylliidae	*Caryophyllia paucipalata*	No	N/A	N/A	N/A	N/A	N/A		[64]	
Caryophylliidae	*Caryophyllia perculta*	No	N/A	N/A	N/A	N/A	N/A		[64]	
Caryophylliidae	*Caryophyllia planilamellata*	No	N/A	N/A	N/A	N/A	N/A	16S: FJ788121	[64]	
Caryophylliidae	*Caryophyllia polygona*	No	N/A	N/A	N/A	N/A	N/A		[64]	
Caryophylliidae	*Caryophyllia profunda*	No	N/A	N/A	N/A	N/A	N/A		[64]	
Caryophylliidae	*Caryophyllia protei*	No	N/A	N/A	N/A	N/A	N/A		[64]	
Caryophylliidae	*Caryophyllia quadragenaria*	No	N/A	N/A	N/A	N/A	N/A		[64]	
Caryophylliidae	*Caryophyllia quangdongensis*	No	N/A	N/A	N/A	N/A	N/A		[64]	
Caryophylliidae	*Caryophyllia ralphae*	No	N/A	N/A	N/A	N/A	N/A	COI: HM018617	[64]	
Caryophylliidae	*Caryophyllia rugosa*	No	N/A	N/A	N/A	N/A	N/A	16S: FJ788123; COI: HM018618	[64]	
Caryophylliidae	*Caryophyllia sarsiae*	No	N/A	N/A	N/A	N/A	N/A		[64]	
Caryophylliidae	*Caryophyllia scobinosa*	No	N/A	N/A	N/A	N/A	N/A	16S: FJ788124	[64]	
Caryophylliidae	*Caryophyllia secta*	No	N/A	N/A	N/A	N/A	N/A		[64]	
Caryophylliidae	*Caryophyllia seguenzae*	No	N/A	N/A	N/A	N/A	N/A		[64]	
Caryophylliidae	*Caryophyllia sewelli*	No	N/A	N/A	N/A	N/A	N/A		[64]	
Caryophylliidae	*Caryophyllia smithii*	No	N/A	N/A	N/A	N/A	N/A		[64]	
Caryophylliidae	*Caryophyllia solida*	No	DD	N/A	N/A	N/A	N/A		[64]	
Caryophylliidae	*Caryophyllia spinicarens*	No	N/A	N/A	N/A	N/A	N/A		[64]	
Caryophylliidae	*Caryophyllia spinigera*	No	N/A	N/A	N/A	N/A	N/A		[64]	
Caryophylliidae	*Caryophyllia squiresi*	No	N/A	N/A	N/A	N/A	N/A		[64]	
Caryophylliidae	*Caryophyllia stellula*	No	N/A	N/A	N/A	N/A	N/A		[64]	
Caryophylliidae	*Caryophyllia tangaroae*	No	N/A	N/A	N/A	N/A	N/A		[64]	
Caryophylliidae	*Caryophyllia transversalis*	No	N/A	N/A	N/A	N/A	N/A	16S: FJ788125	[64]	
Caryophylliidae	*Caryophyllia unicristata*	No	N/A	N/A	N/A	N/A	N/A	16S: FJ788127	[64]	
Caryophylliidae	*Caryophyllia valdiviae*	No	N/A	N/A	N/A	N/A	N/A		[64]	
Caryophylliidae	*Caryophyllia versicolorata*	No	N/A	N/A	N/A	N/A	N/A		[64]	

Family	Species	Reef	Red List	EoE rank	EDGE rank (IUCN100)	EDGE rank (Isaac)	EDGE rank (Pessimistic)	Molecular sources	Morphological sources	Remarks
Caryophylliidae	*Caryophyllia zanzibarensis*	No	N/A	N/A	N/A	N/A	N/A		[64]	
Caryophylliidae	*Caryophyllia zopyros*	No	N/A	N/A	N/A	N/A	N/A		[64]	
Caryophylliidae	*Ceratotrochus magnaghii*	No	N/A	N/A	N/A	N/A	N/A	16S: AF265597		
Caryophylliidae	*Conotrochus funicolumna*	No	N/A	N/A	N/A	N/A	N/A	COI: HM018621		
Caryophylliidae	*Crispatotrochus cornu*	No	N/A	N/A	N/A	N/A	N/A		[64]	
Caryophylliidae	*Crispatotrochus curvatus*	No	N/A	N/A	N/A	N/A	N/A		[64]	
Caryophylliidae	*Crispatotrochus foxi*	No	N/A	N/A	N/A	N/A	N/A		[64]	
Caryophylliidae	*Crispatotrochus galapagensis*	No	DD	N/A	N/A	N/A	N/A		[64]	
Caryophylliidae	*Crispatotrochus gregarius*	No	N/A	N/A	N/A	N/A	N/A		[64]	
Caryophylliidae	*Crispatotrochus inornatus*	No	N/A	N/A	N/A	N/A	N/A		[64]	
Caryophylliidae	*Crispatotrochus irregularis*	No	N/A	N/A	N/A	N/A	N/A		[64]	
Caryophylliidae	*Crispatotrochus niinoi*	No	N/A	N/A	N/A	N/A	N/A		[64]	
Caryophylliidae	*Crispatotrochus rubescens*	No	N/A	N/A	N/A	N/A	N/A		[64]	
Caryophylliidae	*Crispatotrochus rugosus*	No	N/A	N/A	N/A	N/A	N/A	12S: EF597041; 16S: AF265600	[64]	
Caryophylliidae	*Crispatotrochus septumdentatus*	No	N/A	N/A	N/A	N/A	N/A		[64]	
Caryophylliidae	*Crispatotrochus squiresi*	No	N/A	N/A	N/A	N/A	N/A		[64]	
Caryophylliidae	*Crispatotrochus woodsi*	No	N/A	N/A	N/A	N/A	N/A		[64]	
Caryophylliidae	*Dactylotrochus cervicornis*	No	N/A	N/A	N/A	N/A	N/A	COI: HM018624		
Caryophylliidae	*Dasmosmilia lymani*	No	N/A	N/A	N/A	N/A	N/A	16S: FJ788130; COI: HM018625	[68]	*Dasmosmilia* cf. *lymani* in GenBank
Caryophylliidae	*Dasmosmilia variegata*	No	N/A	N/A	N/A	N/A	N/A		[68]	*Dasmosmilia* monophyly assumed
Caryophylliidae	*Deltocyathus calcar*	No	N/A	N/A	N/A	N/A	N/A		[68]	*Deltocyathus* monophyly assumed
Caryophylliidae	*Deltocyathus eccentricus*	No	N/A	N/A	N/A	N/A	N/A		[68]	*Deltocyathus* monophyly assumed
Caryophylliidae	*Deltocyathus inusitatus*	No	N/A	N/A	N/A	N/A	N/A	COI: HM018626		
Caryophylliidae	*Deltocyathus italicus*	No	N/A	N/A	N/A	N/A	N/A		[68]	
Caryophylliidae	*Deltocyathus magnificus*	No	N/A	N/A	N/A	N/A	N/A	COI: HM018627		
Caryophylliidae	*Deltocyathus ornatus*	No	N/A	N/A	N/A	N/A	N/A	COI: HM018628		
Caryophylliidae	*Deltocyathus rotulus*	No	N/A	N/A	N/A	N/A	N/A	COI: HM018629		
Caryophylliidae	*Deltocyathus sarsi*	No	N/A	N/A	N/A	N/A	N/A	COI: HM018630		
Caryophylliidae	*Deltocyathus suluensis*	No	N/A	N/A	N/A	N/A	N/A	COI: HM018631		
Caryophylliidae	*Desmophyllum dianthus*	No	N/A	N/A	N/A	N/A	N/A	12S: GQ868667; 16S: GQ868690		
Caryophylliidae	*Heterocyathus aequicostatus*	Yes	LC	N/A	790	719	719			*Heterocyathus* monophyly assumed; Caryophylliidae clade (M. V. Kitahara, pers comm)
Caryophylliidae	*Heterocyathus alternatus*	Yes	LC	N/A	790	719	719			*Heterocyathus* monophyly assumed; Caryophylliidae clade (M. V. Kitahara, pers comm)
Caryophylliidae	*Heterocyathus sulcatus*	Yes	LC	N/A	790	719	719			*Heterocyathus* monophyly assumed; Caryophylliidae clade (M. V. Kitahara, pers comm)
Caryophylliidae	*Hoplangia durotrix*	No	N/A	N/A	N/A	N/A	N/A	12S: EF597064		
Caryophylliidae	*Lophelia pertusa*	No	N/A	N/A	N/A	N/A	N/A	12S: FR821799; 16S: FR821799; AT6: FR821799; COI: FR821799; CYB: FR821799		
Caryophylliidae	*Paracyathus andersoni*	No	N/A	N/A	N/A	N/A	N/A		[64]	
Caryophylliidae	*Paracyathus arcuatus*	No	N/A	N/A	N/A	N/A	N/A		[64]	
Caryophylliidae	*Paracyathus cavatus*	No	N/A	N/A	N/A	N/A	N/A		[64]	
Caryophylliidae	*Paracyathus conceptus*	No	N/A	N/A	N/A	N/A	N/A		[64]	
Caryophylliidae	*Paracyathus coronatus*	No	N/A	N/A	N/A	N/A	N/A		[64]	
Caryophylliidae	*Paracyathus darwinensis*	No	N/A	N/A	N/A	N/A	N/A		[64]	
Caryophylliidae	*Paracyathus ebonensis*	No	N/A	N/A	N/A	N/A	N/A		[64]	
Caryophylliidae	*Paracyathus fulvus*	No	N/A	N/A	N/A	N/A	N/A		[64]	
Caryophylliidae	*Paracyathus humilis*	No	N/A	N/A	N/A	N/A	N/A		[64]	
Caryophylliidae	*Paracyathus indicus*	No	N/A	N/A	N/A	N/A	N/A		[64]	
Caryophylliidae	*Paracyathus lifuensis*	No	N/A	N/A	N/A	N/A	N/A		[64]	
Caryophylliidae	*Paracyathus molokensis*	No	N/A	N/A	N/A	N/A	N/A		[64]	
Caryophylliidae	*Paracyathus monteryensis*	No	N/A	N/A	N/A	N/A	N/A		[64]	
Caryophylliidae	*Paracyathus parvulus*	No	N/A	N/A	N/A	N/A	N/A		[64]	
Caryophylliidae	*Paracyathus persicus*	No	N/A	N/A	N/A	N/A	N/A		[64]	

Family	Species	Reef	Red List	EoE rank	EDGE rank (IUCN100)	EDGE rank (Isaac)	EDGE rank (Pessimistic)	Molecular sources	Morphological sources	Remarks
Caryophylliidae	*Paracyathus porcellanus*	No	N/A	N/A	N/A	N/A	N/A		[64]	
Caryophylliidae	*Paracyathus profundus*	No	N/A	N/A	N/A	N/A	N/A		[64]	
Caryophylliidae	*Paracyathus pruinosus*	No	N/A	N/A	N/A	N/A	N/A		[64]	
Caryophylliidae	*Paracyathus pulchellus*	No	N/A	N/A	N/A	N/A	N/A	12S: EF597027; 16S: AF265603	[64]	
Caryophylliidae	*Paracyathus rotundatus*	No	N/A	N/A	N/A	N/A	N/A		[64]	
Caryophylliidae	*Paracyathus stearnsii*	No	N/A	N/A	N/A	N/A	N/A		[64]	
Caryophylliidae	*Paracyathus stokesii*	No	N/A	N/A	N/A	N/A	N/A		[64]	
Caryophylliidae	*Paracyathus vittatus*	No	N/A	N/A	N/A	N/A	N/A		[64]	
Caryophylliidae	*Phyllangia americana*	No	N/A	N/A	N/A	N/A	N/A	12S: EF597022; 16S: AF265605	[64,68]	
Caryophylliidae	*Phyllangia consagensis*	No	N/A	N/A	N/A	N/A	N/A		[64]	
Caryophylliidae	*Phyllangia dispersa*	No	N/A	N/A	N/A	N/A	N/A		[64]	
Caryophylliidae	*Phyllangia echinosepes*	No	N/A	N/A	N/A	N/A	N/A		[64]	
Caryophylliidae	*Phyllangia granulata*	No	N/A	N/A	N/A	N/A	N/A		[64]	
Caryophylliidae	*Phyllangia hayamaensis*	No	N/A	N/A	N/A	N/A	N/A		[64]	
Caryophylliidae	*Phyllangia papuensis*	No	N/A	N/A	N/A	N/A	N/A	COI: HM018660	[64]	
Caryophylliidae	*Phyllangia pequegnatae*	No	N/A	N/A	N/A	N/A	N/A		[64]	
Caryophylliidae	*Polycyathus andamanensis*	No	N/A	N/A	N/A	N/A	N/A		[64]	
Caryophylliidae	*Polycyathus atlanticus*	No	N/A	N/A	N/A	N/A	N/A		[64]	
Caryophylliidae	*Polycyathus difficilis*	No	N/A	N/A	N/A	N/A	N/A		[64]	
Caryophylliidae	*Polycyathus fulvus*	No	N/A	N/A	N/A	N/A	N/A		[64]	
Caryophylliidae	*Polycyathus furanaensis*	No	N/A	N/A	N/A	N/A	N/A		[64]	
Caryophylliidae	*Polycyathus fuscomarginatus*	No	N/A	N/A	N/A	N/A	N/A		[64]	
Caryophylliidae	*Polycyathus hodgsoni*	No	N/A	N/A	N/A	N/A	N/A		[64]	
Caryophylliidae	*Polycyathus hondaensis*	No	N/A	N/A	N/A	N/A	N/A		[64]	
Caryophylliidae	*Polycyathus isabela*	No	VU	N/A	N/A	N/A	N/A		[64]	
Caryophylliidae	*Polycyathus marigondoni*	No	N/A	N/A	N/A	N/A	N/A		[64]	
Caryophylliidae	*Polycyathus mayae*	No	N/A	N/A	N/A	N/A	N/A		[64]	
Caryophylliidae	*Polycyathus muellerae*	No	N/A	N/A	N/A	N/A	N/A	12S: EF597026; 16S: AF265606	[64]	
Caryophylliidae	*Polycyathus norfolkensis*	No	N/A	N/A	N/A	N/A	N/A		[64]	
Caryophylliidae	*Polycyathus octuplus*	No	N/A	N/A	N/A	N/A	N/A		[64]	
Caryophylliidae	*Polycyathus palifera*	No	N/A	N/A	N/A	N/A	N/A		[64]	
Caryophylliidae	*Polycyathus persicus*	No	N/A	N/A	N/A	N/A	N/A		[64]	
Caryophylliidae	*Polycyathus senegalensis*	No	N/A	N/A	N/A	N/A	N/A		[64]	
Caryophylliidae	*Polycyathus verrilli*	No	N/A	N/A	N/A	N/A	N/A		[64]	
Caryophylliidae	*Premocyathus cornuformis*	No	N/A	N/A	N/A	N/A	N/A		[68]	Revised from *Caryophyllia cornuformis*
Caryophylliidae	*Rhizosmilia maculata*	No	N/A	N/A	N/A	N/A	N/A	12S: EF597023; 16S: AF265602	[68]	
Caryophylliidae	*Rhizosmilia robusta*	No	N/A	N/A	N/A	N/A	N/A	COI: HM018664		
Caryophylliidae	*Solenosmilia variabilis*	No	N/A	N/A	N/A	N/A	N/A	16S: HM015348		
Caryophylliidae	*Stephanocyathus platypus*	No	N/A	N/A	N/A	N/A	N/A	16S: HM015352		
Caryophylliidae	*Stephanocyathus spiniger*	No	N/A	N/A	N/A	N/A	N/A	16S: HM015359; COI: HM018665		
Caryophylliidae	*Stephanocyathus weberianus*	No	N/A	N/A	N/A	N/A	N/A	16S: AF265594		
Caryophylliidae	*Tethocyathus virgatus*	No	N/A	N/A	N/A	N/A	N/A	16S: FJ788131		
Caryophylliidae	*Thalamophyllia gasti*	No	N/A	N/A	N/A	N/A	N/A	12S: EF597086; 16S: AF265590	[64]	
Caryophylliidae	*Thalamophyllia gombergi*	No	N/A	N/A	N/A	N/A	N/A		[64]	
Caryophylliidae	*Thalamophyllia riisei*	No	N/A	N/A	N/A	N/A	N/A	12S: EF597087	[64]	
Caryophylliidae	*Thalamophyllia tenuescens*	No	N/A	N/A	N/A	N/A	N/A		[64]	
Caryophylliidae	*Trochocyathus efateensis*	No	N/A	N/A	N/A	N/A	N/A	16S: FJ788132; COI: HM018667		
Caryophylliidae	*Trochocyathus rhombocolumna*	No	N/A	N/A	N/A	N/A	N/A	COI: HM018668		
Caryophylliidae	*Vaughanella concinna*	No	N/A	N/A	N/A	N/A	N/A		[64]	
Caryophylliidae	*Vaughanella margaritata*	No	N/A	N/A	N/A	N/A	N/A	16S: AF265595	[64]	*Vaughanella* sp. in GenBank; terminal branch shared with conspecifics
Caryophylliidae	*Vaughanella multipalifera*	No	N/A	N/A	N/A	N/A	N/A		[64]	
Caryophylliidae	*Vaughanella oreophila*	No	N/A	N/A	N/A	N/A	N/A		[64]	
Dendrophylliidae	*Astroides calycularis*	No	N/A	N/A	N/A	N/A	N/A		[63]	
Dendrophylliidae	*Balanophyllia bairdiana*	No	N/A	N/A	N/A	N/A	N/A		[63,64]	
Dendrophylliidae	*Balanophyllia bayeri*	No	N/A	N/A	N/A	N/A	N/A		[63,64]	

Family	Species	Reef	Red List	EoE rank	EDGE rank (IUCN100)	EDGE rank (Isaac)	EDGE rank (Pessimistic)	Molecular sources	Morphological sources	Remarks
Dendrophylliidae	*Balanophyllia bonaespei*	No	N/A	N/A	N/A	N/A	N/A		[63,64]	
Dendrophylliidae	*Balanophyllia capensis*	No	N/A	N/A	N/A	N/A	N/A		[63,64]	
Dendrophylliidae	*Balanophyllia caribbeana*	No	N/A	N/A	N/A	N/A	N/A		[63,64]	*Balanophyllia (Eupsammia)*
Dendrophylliidae	*Balanophyllia carinata*	No	N/A	N/A	N/A	N/A	N/A		[63,64]	*Balanophyllia (Eupsammia)*
Dendrophylliidae	*Balanophyllia cedrosensis*	No	N/A	N/A	N/A	N/A	N/A		[63,64]	
Dendrophylliidae	*Balanophyllia cellulosa*	No	N/A	N/A	N/A	N/A	N/A		[63,64]	
Dendrophylliidae	*Balanophyllia chnous*	No	N/A	N/A	N/A	N/A	N/A		[63,64]	
Dendrophylliidae	*Balanophyllia corniculans*	No	N/A	N/A	N/A	N/A	N/A		[63,64]	
Dendrophylliidae	*Balanophyllia cornu*	No	N/A	N/A	N/A	N/A	N/A	COI: HM018605	[63,64]	
Dendrophylliidae	*Balanophyllia crassiseptum*	No	N/A	N/A	N/A	N/A	N/A		[63,64]	
Dendrophylliidae	*Balanophyllia crassitheca*	No	N/A	N/A	N/A	N/A	N/A		[63,64]	
Dendrophylliidae	*Balanophyllia cumingii*	No	N/A	N/A	N/A	N/A	N/A		[63,64]	
Dendrophylliidae	*Balanophyllia cyathoides*	No	N/A	N/A	N/A	N/A	N/A		[63,64]	
Dendrophylliidae	*Balanophyllia dentata*	No	N/A	N/A	N/A	N/A	N/A		[63,64]	
Dendrophylliidae	*Balanophyllia desmophyllioides*	No	N/A	N/A	N/A	N/A	N/A	COI: HM018607	[63,64]	
Dendrophylliidae	*Balanophyllia diademata*	No	N/A	N/A	N/A	N/A	N/A		[63,64]	
Dendrophylliidae	*Balanophyllia diffusa*	No	N/A	N/A	N/A	N/A	N/A		[63,64]	
Dendrophylliidae	*Balanophyllia dilatata*	No	N/A	N/A	N/A	N/A	N/A		[63,64]	
Dendrophylliidae	*Balanophyllia dineta*	No	N/A	N/A	N/A	N/A	N/A		[63,64]	
Dendrophylliidae	*Balanophyllia diomedeae*	No	N/A	N/A	N/A	N/A	N/A		[63,64]	
Dendrophylliidae	*Balanophyllia dubia*	No	N/A	N/A	N/A	N/A	N/A		[63,64]	
Dendrophylliidae	*Balanophyllia elegans*	No	N/A	N/A	N/A	N/A	N/A	COI: DQ445805	[63,64]	
Dendrophylliidae	*Balanophyllia europaea*	Yes	DD	N/A	756	836	836		[63,64,68]	
Dendrophylliidae	*Balanophyllia floridana*	No	N/A	N/A	N/A	N/A	N/A		[63,64]	
Dendrophylliidae	*Balanophyllia galapagensis*	No	N/A	N/A	N/A	N/A	N/A		[63,64]	
Dendrophylliidae	*Balanophyllia gemma*	No	N/A	N/A	N/A	N/A	N/A		[63,64]	
Dendrophylliidae	*Balanophyllia gemmifera*	No	N/A	N/A	N/A	N/A	N/A		[63,64]	
Dendrophylliidae	*Balanophyllia generatrix*	No	N/A	N/A	N/A	N/A	N/A		[63,64]	
Dendrophylliidae	*Balanophyllia gigas*	No	N/A	N/A	N/A	N/A	N/A		[63,64]	
Dendrophylliidae	*Balanophyllia hadros*	No	N/A	N/A	N/A	N/A	N/A		[63,64]	
Dendrophylliidae	*Balanophyllia helenae*	No	N/A	N/A	N/A	N/A	N/A		[63,64]	
Dendrophylliidae	*Balanophyllia imperialis*	No	N/A	N/A	N/A	N/A	N/A		[63,64]	*Balanophyllia (Eupsammia)*
Dendrophylliidae	*Balanophyllia iwayamaensis*	No	N/A	N/A	N/A	N/A	N/A		[63,64]	
Dendrophylliidae	*Balanophyllia japonica*	No	N/A	N/A	N/A	N/A	N/A		[63,64]	
Dendrophylliidae	*Balanophyllia kalakauai*	No	N/A	N/A	N/A	N/A	N/A		[63,64]	
Dendrophylliidae	*Balanophyllia laysanensis*	No	N/A	N/A	N/A	N/A	N/A		[63,64]	
Dendrophylliidae	*Balanophyllia malouinensis*	No	N/A	N/A	N/A	N/A	N/A		[63,64]	
Dendrophylliidae	*Balanophyllia merguiensis*	No	N/A	N/A	N/A	N/A	N/A		[63,64]	
Dendrophylliidae	*Balanophyllia palifera*	No	N/A	N/A	N/A	N/A	N/A		[63,64]	
Dendrophylliidae	*Balanophyllia parallela*	No	N/A	N/A	N/A	N/A	N/A		[63,64]	
Dendrophylliidae	*Balanophyllia parvula*	No	N/A	N/A	N/A	N/A	N/A		[63,64]	
Dendrophylliidae	*Balanophyllia pittieri*	No	N/A	N/A	N/A	N/A	N/A		[63,64]	*Balanophyllia (Eupsammia)*
Dendrophylliidae	*Balanophyllia profundicella*	No	N/A	N/A	N/A	N/A	N/A		[63,64]	
Dendrophylliidae	*Balanophyllia rediviva*	No	N/A	N/A	N/A	N/A	N/A		[63,64]	
Dendrophylliidae	*Balanophyllia regalis*	No	N/A	N/A	N/A	N/A	N/A		[63,64]	*Balanophyllia (Eupsammia)*
Dendrophylliidae	*Balanophyllia regia*	No	N/A	N/A	N/A	N/A	N/A	12S: EF597047; 16S: AF265587	[63,64]	
Dendrophylliidae	*Balanophyllia scabra*	No	N/A	N/A	N/A	N/A	N/A		[63,64]	
Dendrophylliidae	*Balanophyllia scabrosa*	No	N/A	N/A	N/A	N/A	N/A		[63,64]	
Dendrophylliidae	*Balanophyllia serrata*	No	N/A	N/A	N/A	N/A	N/A		[63,64]	
Dendrophylliidae	*Balanophyllia spongiosa*	No	N/A	N/A	N/A	N/A	N/A		[63,64]	
Dendrophylliidae	*Balanophyllia stimpsonii*	No	N/A	N/A	N/A	N/A	N/A		[63,64]	*Balanophyllia (Eupsammia)*
Dendrophylliidae	*Balanophyllia striata*	No	N/A	N/A	N/A	N/A	N/A		[63,64]	

Family	Species	Reef	Red List	EoE rank	EDGE rank (IUCN100)	EDGE rank (Isaac)	EDGE rank (Pessimistic)	Molecular sources	Morphological sources	Remarks
Dendrophylliidae	*Balanophyllia taprobanae*	No	N/A	N/A	N/A	N/A	N/A		[63,64]	
Dendrophylliidae	*Balanophyllia tenuis*	No	N/A	N/A	N/A	N/A	N/A		[63,64]	
Dendrophylliidae	*Balanophyllia thalassae*	No	N/A	N/A	N/A	N/A	N/A		[63,64]	
Dendrophylliidae	*Balanophyllia vanderhorsti*	No	N/A	N/A	N/A	N/A	N/A		[63,64]	
Dendrophylliidae	*Balanophyllia wellsi*	No	N/A	N/A	N/A	N/A	N/A		[63,64]	
Dendrophylliidae	*Balanophyllia yongei*	No	N/A	N/A	N/A	N/A	N/A		[63,64]	
Dendrophylliidae	*Bathypsammia falloscoialis*	No	N/A	N/A	N/A	N/A	N/A		[63]	
Dendrophylliidae	*Bathypsammia tintinnabulum*	No	N/A	N/A	N/A	N/A	N/A		[63]	
Dendrophylliidae	*Cladopsammia echinata*	No	N/A	N/A	N/A	N/A	N/A		[63]	
Dendrophylliidae	*Cladopsammia eguchii*	No	N/A	N/A	N/A	N/A	N/A		[63]	
Dendrophylliidae	*Cladopsammia gracilis*	No	N/A	N/A	N/A	N/A	N/A	12S: EF597049; 16S: AF265588	[63]	
Dendrophylliidae	*Cladopsammia manuelensis*	No	N/A	N/A	N/A	N/A	N/A		[63]	
Dendrophylliidae	*Cladopsammia rolandi*	No	N/A	N/A	N/A	N/A	N/A		[63]	
Dendrophylliidae	*Cladopsammia willeyi*	No	N/A	N/A	N/A	N/A	N/A		[63]	
Dendrophylliidae	*Dendrophyllia aculeata*	No	N/A	N/A	N/A	N/A	N/A		[63]	
Dendrophylliidae	*Dendrophyllia alcocki*	No	N/A	N/A	N/A	N/A	N/A		[63]	
Dendrophylliidae	*Dendrophyllia alternata*	No	N/A	N/A	N/A	N/A	N/A	16S: AF550366	[63]	
Dendrophylliidae	*Dendrophyllia arbuscula*	No	N/A	N/A	N/A	N/A	N/A		[63]	
Dendrophylliidae	*Dendrophyllia boschmai*	No	N/A	N/A	N/A	N/A	N/A		[63]	
Dendrophylliidae	*Dendrophyllia californica*	No	N/A	N/A	N/A	N/A	N/A		[63]	
Dendrophylliidae	*Dendrophyllia carleenae*	No	N/A	N/A	N/A	N/A	N/A		[63]	
Dendrophylliidae	*Dendrophyllia cecilliana*	No	N/A	N/A	N/A	N/A	N/A		[63]	
Dendrophylliidae	*Dendrophyllia cladonia*	No	N/A	N/A	N/A	N/A	N/A		[63]	
Dendrophylliidae	*Dendrophyllia cornigera*	No	N/A	N/A	N/A	N/A	N/A		[63]	
Dendrophylliidae	*Dendrophyllia cribrosa*	No	N/A	N/A	N/A	N/A	N/A		[63]	
Dendrophylliidae	*Dendrophyllia dilatata*	No	N/A	N/A	N/A	N/A	N/A		[63]	
Dendrophylliidae	*Dendrophyllia florulenta*	No	N/A	N/A	N/A	N/A	N/A		[63]	
Dendrophylliidae	*Dendrophyllia fotojiku*	No	N/A	N/A	N/A	N/A	N/A		[63]	
Dendrophylliidae	*Dendrophyllia granosa*	No	N/A	N/A	N/A	N/A	N/A		[63]	
Dendrophylliidae	*Dendrophyllia ijimai*	No	N/A	N/A	N/A	N/A	N/A		[63]	
Dendrophylliidae	*Dendrophyllia incisa*	No	N/A	N/A	N/A	N/A	N/A		[63]	
Dendrophylliidae	*Dendrophyllia indica*	No	N/A	N/A	N/A	N/A	N/A		[63]	
Dendrophylliidae	*Dendrophyllia johnsoni*	No	DD	N/A	N/A	N/A	N/A		[63]	
Dendrophylliidae	*Dendrophyllia laboreli*	No	N/A	N/A	N/A	N/A	N/A		[63]	
Dendrophylliidae	*Dendrophyllia minima*	No	N/A	N/A	N/A	N/A	N/A		[63]	
Dendrophylliidae	*Dendrophyllia minuscula*	No	N/A	N/A	N/A	N/A	N/A		[63]	
Dendrophylliidae	*Dendrophyllia oldroydae*	No	N/A	N/A	N/A	N/A	N/A		[63]	
Dendrophylliidae	*Dendrophyllia paragracilis*	No	N/A	N/A	N/A	N/A	N/A		[63]	
Dendrophylliidae	*Dendrophyllia radians*	No	N/A	N/A	N/A	N/A	N/A		[63]	
Dendrophylliidae	*Dendrophyllia ramea*	No	N/A	N/A	N/A	N/A	N/A		[63]	
Dendrophylliidae	*Dendrophyllia robusta*	No	N/A	N/A	N/A	N/A	N/A		[63]	
Dendrophylliidae	*Dendrophyllia suprarbuscula*	No	N/A	N/A	N/A	N/A	N/A		[63]	
Dendrophylliidae	*Dendrophyllia velata*	No	N/A	N/A	N/A	N/A	N/A		[63]	
Dendrophylliidae	*Dichopsammia granulosa*	No	N/A	N/A	N/A	N/A	N/A		[63]	
Dendrophylliidae	*Duncanopsammia axifuga*	Yes	NT	N/A	445	603	603		[63]	
Dendrophylliidae	*Eguchipsammia cornucopia*	No	N/A	N/A	N/A	N/A	N/A		[63]	
Dendrophylliidae	*Eguchipsammia fistula*	No	N/A	N/A	N/A	N/A	N/A		[63]	
Dendrophylliidae	*Eguchipsammia gaditana*	No	N/A	N/A	N/A	N/A	N/A		[63]	
Dendrophylliidae	*Eguchipsammia japonica*	No	N/A	N/A	N/A	N/A	N/A		[63]	

Family	Species	Reef	Red List	EoE rank	EDGE rank (IUCN100)	EDGE rank (Isaac)	EDGE rank (Pessimistic)	Molecular sources	Morphological sources	Remarks
Dendrophylliidae	*Eguchipsammia serpentina*	No	N/A	N/A	N/A	N/A	N/A		[63]	
Dendrophylliidae	*Eguchipsammia strigosa*	No	N/A	N/A	N/A	N/A	N/A		[63]	
Dendrophylliidae	*Eguchipsammia wellsi*	No	N/A	N/A	N/A	N/A	N/A		[63]	
Dendrophylliidae	*Enallopsammia profunda*	No	N/A	N/A	N/A	N/A	N/A		[63,64]	
Dendrophylliidae	*Enallopsammia pusilla*	No	N/A	N/A	N/A	N/A	N/A		[63,64]	
Dendrophylliidae	*Enallopsammia rostrata*	No	N/A	N/A	N/A	N/A	N/A	16S: U40294; COI: HM018632	[63,64]	
Dendrophylliidae	*Endopachys bulbosa*	No	N/A	N/A	N/A	N/A	N/A		[63]	
Dendrophylliidae	*Endopachys grayi*	No	N/A	N/A	N/A	N/A	N/A		[63]	
Dendrophylliidae	*Endopsammia philippensis*	No	N/A	N/A	N/A	N/A	N/A		[63]	
Dendrophylliidae	*Endopsammia pourtalesi*	No	DD	N/A	N/A	N/A	N/A		[63]	
Dendrophylliidae	*Endopsammia regularis*	No	N/A	N/A	N/A	N/A	N/A		[63]	
Dendrophylliidae	*Heteropsammia cochleata*	Yes	LC	N/A	837	837	837	12S: EF597050	[63]	*Heteropsammia* sp. in GenBank; terminal branch shared with conspecifics
Dendrophylliidae	*Heteropsammia eupsammides*	Yes	NT	N/A	670	832	832		[63]	
Dendrophylliidae	*Heteropsammia moretonensis*	No	N/A	N/A	N/A	N/A	N/A		[63]	
Dendrophylliidae	*Leptopsammia britannica*	No	N/A	N/A	N/A	N/A	N/A		[63,64]	
Dendrophylliidae	*Leptopsammia chevalieri*	No	N/A	N/A	N/A	N/A	N/A		[63,64]	
Dendrophylliidae	*Leptopsammia columna*	No	N/A	N/A	N/A	N/A	N/A		[63,64]	
Dendrophylliidae	*Leptopsammia crassa*	No	N/A	N/A	N/A	N/A	N/A		[63,64]	
Dendrophylliidae	*Leptopsammia formosa*	No	N/A	N/A	N/A	N/A	N/A		[63,64]	
Dendrophylliidae	*Leptopsammia poculum*	No	N/A	N/A	N/A	N/A	N/A		[63,64]	
Dendrophylliidae	*Leptopsammia pruvoti*	No	N/A	N/A	N/A	N/A	N/A	12S: EF597068; 16S: AF265579	[63,64]	
Dendrophylliidae	*Leptopsammia queenslandiae*	No	N/A	N/A	N/A	N/A	N/A		[63,64]	
Dendrophylliidae	*Leptopsammia stokesiana*	No	N/A	N/A	N/A	N/A	N/A		[63,64]	
Dendrophylliidae	*Leptopsammia trinitatis*	No	N/A	N/A	N/A	N/A	N/A		[63,64]	
Dendrophylliidae	*Notophyllia etheridgi*	No	N/A	N/A	N/A	N/A	N/A		[63]	
Dendrophylliidae	*Notophyllia hecki*	No	N/A	N/A	N/A	N/A	N/A		[63]	
Dendrophylliidae	*Notophyllia piscacauda*	No	N/A	N/A	N/A	N/A	N/A		[63]	
Dendrophylliidae	*Notophyllia recta*	No	N/A	N/A	N/A	N/A	N/A		[63]	
Dendrophylliidae	*Pourtalopsammia togata*	No	N/A	N/A	N/A	N/A	N/A		[63]	
Dendrophylliidae	*Rhizopsammia annae*	No	N/A	N/A	N/A	N/A	N/A		[63,64]	
Dendrophylliidae	*Rhizopsammia bermudensis*	No	N/A	N/A	N/A	N/A	N/A		[63,64]	
Dendrophylliidae	*Rhizopsammia compacta*	No	N/A	N/A	N/A	N/A	N/A		[63,64]	
Dendrophylliidae	*Rhizopsammia goesi*	No	N/A	N/A	N/A	N/A	N/A		[63,64]	
Dendrophylliidae	*Rhizopsammia minuta*	No	N/A	N/A	N/A	N/A	N/A		[63,64]	
Dendrophylliidae	*Rhizopsammia nuda*	No	N/A	N/A	N/A	N/A	N/A		[63,64]	
Dendrophylliidae	*Rhizopsammia pulchra*	No	N/A	N/A	N/A	N/A	N/A		[63,64]	
Dendrophylliidae	*Rhizopsammia verrilli*	No	N/A	N/A	N/A	N/A	N/A		[63,64]	
Dendrophylliidae	*Rhizopsammia wellingtoni*	No	CR	N/A	N/A	N/A	N/A		[63,64]	
Dendrophylliidae	*Rhizopsammia wettsteini*	No	N/A	N/A	N/A	N/A	N/A		[63,64]	
Dendrophylliidae	*Thecopsammia elongata*	No	N/A	N/A	N/A	N/A	N/A		[63]	
Dendrophylliidae	*Thecopsammia socialis*	No	N/A	N/A	N/A	N/A	N/A		[63]	
Dendrophylliidae	*Trochopsammia infundibulum*	No	N/A	N/A	N/A	N/A	N/A		[63]	
Dendrophylliidae	*Tubastraea coccinea*	No	N/A	N/A	N/A	N/A	N/A	12S: EF597045; 16S: L76022; COI: DQ445806	[63,64]	
Dendrophylliidae	*Tubastraea diaphana*	No	N/A	N/A	N/A	N/A	N/A		[63,64]	
Dendrophylliidae	*Tubastraea faulkneri*	No	N/A	N/A	N/A	N/A	N/A		[63,64]	
Dendrophylliidae	*Tubastraea floreana*	No	CR	N/A	N/A	N/A	N/A		[63,64]	
Dendrophylliidae	*Tubastraea micranthus*	No	N/A	N/A	N/A	N/A	N/A		[63,64]	
Dendrophylliidae	*Tubastraea tagusensis*	No	N/A	N/A	N/A	N/A	N/A		[63,64]	
Dendrophylliidae	*Turbinaria bifrons*	Yes	VU	N/A	179	410	402		[63,64]	
Dendrophylliidae	*Turbinaria conspicua*	Yes	LC	N/A	805	750	750		[63,64]	
Dendrophylliidae	*Turbinaria crater*	Yes	DD	N/A	547	667	667		[63,64]	Previously excluded [11]
Dendrophylliidae	*Turbinaria frondens*	Yes	LC	N/A	805	750	750		[63,64]	
Dendrophylliidae	*Turbinaria heronensis*	Yes	VU	N/A	179	410	402		[63,64]	
Dendrophylliidae	*Turbinaria irregularis*	Yes	LC	N/A	805	750	750		[63,64]	

Family	Species	Reef	Red List	EoE rank	EDGE rank (IUCN100)	EDGE rank (Isaac)	EDGE rank (Pessimistic)	Molecular sources	Morphological sources	Remarks
Dendrophylliidae	*Turbinaria mesenterina*	Yes	VU	N/A	179	410	402		[63,64]	
Dendrophylliidae	*Turbinaria patula*	Yes	VU	N/A	179	410	402		[63,64]	
Dendrophylliidae	*Turbinaria peltata*	Yes	VU	N/A	34	30	24	12S: EF597044; COI: AB441240; CYB: AB441325	[63,64]	
Dendrophylliidae	*Turbinaria radicalis*	Yes	NT	N/A	454	634	634		[63,64]	
Dendrophylliidae	*Turbinaria reniformis*	Yes	VU	N/A	179	410	402		[63,64]	
Dendrophylliidae	*Turbinaria stellulata*	Yes	VU	N/A	179	410	402		[63,64]	
Euphylliidae	*Catalaphyllia jardinei*	Yes	VU	6	53	65	60	12S: EF596997; 16S: L76000	[64]	
Euphylliidae	*Euphyllia ancora*	Yes	VU	N/A	41	43	38	12S: JF825139; 16S: JF825139; AT6: JF825139; COI: JF825139; CYB: JF825139; ND5: JF825139		
Euphylliidae	*Euphyllia cristata*	Yes	VU	N/A	62	78	73			Closest to *E. glabrescens* [74]
Euphylliidae	*Euphyllia divisa*	Yes	NT	N/A	202	106	99	COI: AB441203; CYB: AB441288		
Euphylliidae	*Euphyllia glabrescens*	Yes	NT	N/A	196	85	80	COI: AB441206; CYB: AB441291		
Euphylliidae	*Euphyllia paraancora*	Yes	VU	N/A	43	45	40			Closest to *E. ancora* & *E. divisa* [83]
Euphylliidae	*Euphyllia paradivisa*	Yes	VU	N/A	62	78	73			Closest to *E. glabrescens* [91]
Euphylliidae	*Euphyllia paraglabrescens*	Yes	VU	N/A	62	78	73			Closest to *E. glabrescens* [83]
Euphylliidae	*Euphyllia yaeyamaensis*	Yes	NT	N/A	255	141	131			Closest to *E. divisa* [92]
Euphylliidae	*Nemenzophyllia turbida*	Yes	VU	N/A	29	20	14			Closest to *Plerogyra* [90]
Euphylliidae	*Physogyra lichtensteini*	Yes	VU	8	27	18	12	12S: EF597030; COI: AB289562; CYB: AB289564	[68]	
Euphylliidae	*Plerogyra cauliformis*	Yes	DD	N/A	270	108	100			*Plerogyra* monophyly assumed; closest to *P. simplex* [77]
Euphylliidae	*Plerogyra diabolotus*	Yes	DD	N/A	270	108	100			*Plerogyra* monophyly assumed
Euphylliidae	*Plerogyra discus*	Yes	VU	N/A	28	19	13			*Plerogyra* monophyly assumed; closest to *P. sinuosa* [93]
Euphylliidae	*Plerogyra multilobata*	Yes	DD	N/A	270	108	100			*Plerogyra* monophyly assumed; closest to *P. diabolotus* [77]
Euphylliidae	*Plerogyra simplex*	Yes	NT	N/A	185	62	57			*Plerogyra* monophyly assumed
Euphylliidae	*Plerogyra sinuosa*	Yes	NT	N/A	185	62	57	COI: HM018663		*Plerogyra* sp. in GenBank; ancestral branch shared with conspecifics
Faviidae	*Australogyra zelli*	Yes	VU	N/A	144	267	265			Closest to *Platygyra* [88]
Faviidae	*Barabattoia amicorum*	Yes	LC	N/A	689	539	539	COI: AB441193; CYB: AB441278		
Faviidae	*Barabattoia laddi*	Yes	VU	N/A	101	172	164			Clade VII-B monophyly shown [81]; *Barabattoia* monophyly assumed
Faviidae	*Caulastraea connata*	Yes	VU	N/A	111	188	180			Closest to *C. tumida* [92]
Faviidae	*Caulastraea curvata*	Yes	VU	N/A	136	236	233			Closest to *C. furcata* [96]
Faviidae	*Caulastraea echinulata*	Yes	VU	N/A	79	99	92	COI: FJ345414		
Faviidae	*Caulastraea furcata*	Yes	LC	N/A	745	635	635	12S: EF597035; 16S: L75997; COI: AB117274; CYB: AB117355		
Faviidae	*Caulastraea tumida*	Yes	NT	N/A	306	282	280	COI: HQ203249		
Faviidae	*Cladocora arbuscula*	Yes	LC	N/A	580	388	380	COI: AB117292; CYB: AB117377	[64,68]	
Faviidae	*Cladocora caespitosa*	Yes	DD	N/A	274	117	108	12S: EF597017; 16S: AF265612	[64]	
Faviidae	*Cladocora debilis*	No	N/A	N/A	N/A	N/A	N/A		[64,68]	
Faviidae	*Cladocora pacifica*	No	N/A	N/A	N/A	N/A	N/A		[64]	
Faviidae	*Colpophyllia amaranthus*	Yes	DD	N/A	368	357	352		[61]	Previously excluded [11]
Faviidae	*Colpophyllia breviserialis*	Yes	DD	N/A	368	357	352		[61]	Previously excluded [11]
Faviidae	*Colpophyllia natans*	Yes	LC	N/A	666	457	449	12S: DQ643833; 16S: DQ643833; AT6: DQ643833; COI: DQ643833; CYB: DQ643833	[61]	
Faviidae	*Cyphastrea agassizi*	Yes	VU	N/A	100	171	163			*Cyphastrea* monophyly shown [81]
Faviidae	*Cyphastrea chalcidicum*	Yes	LC	N/A	420	137	127	COI: AB117259; CYB: AB117336		
Faviidae	*Cyphastrea decadia*	Yes	LC	N/A	687	537	537			*Cyphastrea* monophyly shown [81]; closest to *C. japonica* [85]
Faviidae	*Cyphastrea hexasepta*	Yes	VU	N/A	42	44	39			*Cyphastrea* monophyly shown [81]; closest to *C. microphthalma* [93]
Faviidae	*Cyphastrea japonica*	Yes	LC	N/A	687	537	537			*Cyphastrea* monophyly shown [81]
Faviidae	*Cyphastrea microphthalma*	Yes	LC	N/A	430	152	142	COI: FJ345416		
Faviidae	*Cyphastrea ocellina*	Yes	VU	N/A	96	162	154	12S: EF596996; 16S: L76132		

Family	Species	Reef	Red List	EoE rank	EDGE rank (IUCN100)	EDGE rank (Isaac)	EDGE rank (Pessimistic)	Molecular sources	Morphological sources	Remarks
Faviidae	*Cyphastrea serailia*	Yes	LC	N/A	423	142	132	COI: AB117258; CYB: AB117334		
Faviidae	*Diploastrea heliopora*	Yes	NT	ED	167	40	35	COI: AB117290; CYB: AB117375		
Faviidae	*Diploria clivosa*	Yes	LC	N/A	667	458	450	12S: EF597001; COI: AB117226; CYB: AB117304	[61]	
Faviidae	*Diploria labyrinthiformis*	Yes	LC	N/A	668	459	451	12S: EF597002; COI: AB117224; CYB: AB117302	[61]	
Faviidae	*Diploria strigosa*	Yes	LC	N/A	597	424	416	12S: EF597003; COI: AB117225; CYB: AB117303	[61]	
Faviidae	*Echinopora ashmorensis*	Yes	VU	N/A	115	203	196			*Echinopora* monophyly shown [81]; closest to *E. lamellosa* [91]
Faviidae	*Echinopora forskaliana*	Yes	NT	N/A	413	528	528			*Echinopora* monophyly shown [81]
Faviidae	*Echinopora fruticulosa*	Yes	NT	N/A	413	528	528			*Echinopora* monophyly shown [81]
Faviidae	*Echinopora gemmacea*	Yes	LC	N/A	456	192	184	COI: AB117263; CYB: AB117342		
Faviidae	*Echinopora grandicula*	Yes	DD	N/A	484	631	631			*Echinopora* monophyly shown [81]
Faviidae	*Echinopora hirsutissima*	Yes	LC	N/A	787	707	707			*Echinopora* monophyly shown [81]
Faviidae	*Echinopora horrida*	Yes	NT	N/A	165	36	30	COI: HQ203253		
Faviidae	*Echinopora irregularis*	Yes	DD	N/A	484	631	631			*Echinopora* monophyly shown [81]; closest to *E. hirsutissima* [93]
Faviidae	*Echinopora lamellosa*	Yes	LC	N/A	584	397	389	16S: L76003; COI: FJ345419		
Faviidae	*Echinopora mammiformis*	Yes	NT	N/A	193	77	72	COI: HQ203254		
Faviidae	*Echinopora pacificus*	Yes	NT	N/A	191	76	71	COI: AB117262; CYB: AB117341		
Faviidae	*Echinopora robusta*	Yes	VU	N/A	166	334	332			*Echinopora* monophyly shown [81]; closest to *E. forskaliana* [93]
Faviidae	*Echinopora taylorae*	Yes	NT	N/A	413	528	528			*Echinopora* monophyly shown [81]
Faviidae	*Echinopora tiranensis*	Yes	DD	N/A	484	631	631			*Echinopora* monophyly shown [81]
Faviidae	*Erythrastrea flabellata*	Yes	NT	N/A	373	432	424			Closest to *Caulastraea* [86]
Faviidae	*Favia albidus*	Yes	NT	N/A	361	419	411			Clade VII-B monophyly shown [81]; closest to *F. matthaii* [93]
Faviidae	*Favia danae*	Yes	LC	N/A	713	594	594	COI: EU371663		
Faviidae	*Favia favus*	Yes	LC	N/A	619	448	440	12S: AF177048; COI: AB117267; CYB: AB117346		
Faviidae	*Favia fragum*	Yes	LC	N/A	712	589	589	12S: EF597005; 16S: U40295; COI: AB117222; CYB: AB117301	[61]	
Faviidae	*Favia gravida*	Yes	DD	N/A	421	444	436		[61]	Previously excluded [11]
Faviidae	*Favia helianthoides*	Yes	NT	N/A	342	378	370			Clade VII-B monophyly shown [81]; closest to *F. laxa* [92]
Faviidae	*Favia lacuna*	Yes	NT	N/A	409	520	520			Clade VII-B monophyly shown [81]
Faviidae	*Favia laxa*	Yes	NT	N/A	341	377	369	COI: EU371707		*Favia* cf. *laxa* in GenBank
Faviidae	*Favia leptophylla*	Yes	DD	N/A	441	484	478	COI: AB117229; CYB: AB117307	[61,68]	
Faviidae	*Favia lizardensis*	Yes	NT	N/A	359	417	409	COI: HM018633		
Faviidae	*Favia maritima*	Yes	NT	N/A	254	140	130	COI: HQ203258		*Favia* cf. *maritima* in GenBank
Faviidae	*Favia marshae*	Yes	NT	N/A	345	384	376			Clade VII-F monophyly shown [81]; closest to *F. rotundata* [93]
Faviidae	*Favia matthaii*	Yes	NT	N/A	360	418	410	COI: HQ203259		
Faviidae	*Favia maxima*	Yes	NT	N/A	284	219	213	COI: HQ203260		
Faviidae	*Favia pallida*	Yes	LC	N/A	713	594	594	COI: AB117266; CYB: AB117345		
Faviidae	*Favia rosaria*	Yes	VU	N/A	151	289	288	COI: HQ203262		
Faviidae	*Favia rotumana*	Yes	LC	N/A	452	184	176	COI: FJ345427		
Faviidae	*Favia rotundata*	Yes	NT	N/A	302	269	267	COI: HQ203263		Clade VII-F monophyly shown [81]
Faviidae	*Favia speciosa*	Yes	LC	N/A	683	519	519	COI: AB441194; CYB: AB441279		
Faviidae	*Favia stelligera*	Yes	NT	N/A	310	296	295	COI: AB117264; CYB: AB117343		
Faviidae	*Favia truncatus*	Yes	LC	N/A	761	655	655	COI: HQ203266		
Faviidae	*Favia veroni*	Yes	NT	N/A	334	362	354			Clade VII-B monophyly shown [81]; closest to *F. maxima* [85]
Faviidae	*Favia vietnamensis*	Yes	NT	N/A	409	520	520			Clade VII-B monophyly shown [81]
Faviidae	*Favites abdita*	Yes	NT	N/A	268	160	152	COI: HQ203267	[68]	
Faviidae	*Favites acuticollis*	Yes	NT	N/A	390	469	462			Clade VII-F monophyly shown [81]
Faviidae	*Favites bestae*	Yes	NT	N/A	390	469	462			Clade VII-F monophyly shown [81]
Faviidae	*Favites chinensis*	Yes	NT	N/A	347	387	379	COI: AB117269; CYB: AB117349		
Faviidae	*Favites complanata*	Yes	NT	N/A	294	245	242	COI: EU371689		

Family	Species	Reef	Red List	EoE rank	EDGE rank (IUCN100)	EDGE rank (Isaac)	EDGE rank (Pessimistic)	Molecular sources	Morphological sources	Remarks
Faviidae	*Favites flexuosa*	Yes	NT	N/A	289	239	236	COI: HQ203269		
Faviidae	*Favites halicora*	Yes	NT	N/A	290	240	237	COI: AB117268; CYB: AB117348		
Faviidae	*Favites micropentagona*	Yes	NT	N/A	277	194	186			Closest to *F. pentagona* [93]
Faviidae	*Favites paraflexuosa*	Yes	NT	N/A	226	123	113	COI: EU371694		
Faviidae	*Favites pentagona*	Yes	LC	N/A	574	373	365	COI: HQ203271		
Faviidae	*Favites russelli*	Yes	NT	N/A	223	118	109	COI: HQ203272		
Faviidae	*Favites spinosa*	Yes	VU	N/A	152	291	290			Clade VII-F monophyly shown [81]
Faviidae	*Favites stylifera*	Yes	NT	N/A	235	127	117	COI: HQ203273	[64]	Clade VII-G monophyly shown [81]
Faviidae	*Favites vasta*	Yes	NT	N/A	390	469	462			Clade VII-F monophyly shown [81]
Faviidae	*Goniastrea aspera*	Yes	LC	N/A	709	580	580	COI: AB117271; CYB: AB117351		
Faviidae	*Goniastrea australensis*	Yes	LC	N/A	500	261	259	COI: HQ203274		
Faviidae	*Goniastrea columella*	Yes	NT	N/A	349	390	382			Closest to *G. pectinata* [92]
Faviidae	*Goniastrea deformis*	Yes	VU	N/A	11	3	2	COI: AB441195; CYB: AB441280		
Faviidae	*Goniastrea edwardsi*	Yes	LC	N/A	722	605	605	COI: EU371697		
Faviidae	*Goniastrea favulus*	Yes	NT	N/A	348	389	381	COI: EU371698		
Faviidae	*Goniastrea minuta*	Yes	NT	N/A	311	297	296			Closest to *G. retiformis* [93]
Faviidae	*Goniastrea palauensis*	Yes	NT	N/A	240	135	124	COI: EU371699		Clade VII-B monophyly shown [81]
Faviidae	*Goniastrea pectinata*	Yes	LC	N/A	721	604	604	COI: AB117270; CYB: AB117350		
Faviidae	*Goniastrea peresi*	Yes	NT	N/A	371	430	422			Closest to *G. aspera* [92]
Faviidae	*Goniastrea ramosa*	Yes	VU	N/A	91	146	136			Closest to *G. retiformis* [93]
Faviidae	*Goniastrea retiformis*	Yes	LC	N/A	673	476	470	12S: EF597033; COI: HQ203275		
Faviidae	*Goniastrea thecata*	Yes	NT	N/A	371	430	422			Closest to *G. aspera* [93]
Faviidae	*Leptastrea aequalis*	Yes	VU	N/A	71	84	79			*Leptastrea* + Fungiidae monophyly shown [82]
Faviidae	*Leptastrea bewickensis*	Yes	NT	N/A	281	213	207			*Leptastrea* + Fungiidae monophyly shown [82]
Faviidae	*Leptastrea bottae*	Yes	NT	N/A	281	213	207			*Leptastrea* + Fungiidae monophyly shown [82]
Faviidae	*Leptastrea inaequalis*	Yes	NT	N/A	281	213	207			*Leptastrea* + Fungiidae monophyly shown [82]
Faviidae	*Leptastrea pruinosa*	Yes	LC	N/A	502	263	261	COI: AB441196; CYB: AB441281		
Faviidae	*Leptastrea purpurea*	Yes	LC	N/A	532	301	300	COI: EU371702		
Faviidae	*Leptastrea transversa*	Yes	LC	N/A	531	300	299	COI: HM018655		
Faviidae	*Leptoria irregularis*	Yes	VU	N/A	89	144	134	COI: AB117272; CYB: AB117353		
Faviidae	*Leptoria phrygia*	Yes	NT	N/A	279	205	199	16S: L76011; COI: AB117273; CYB: AB117354		
Faviidae	*Manicina areolata*	Yes	LC	ED	665	456	448	12S: EF597012; COI: AB117227; CYB: AB117305	[61]	
Faviidae	*Montastraea annularis*	Yes	EN	N/A	2	7	21	12S: AP008973; 16S: AP008973; AT6: AP008973; COI: AP008973; CYB: AP008973		
Faviidae	*Montastraea annuligera*	Yes	NT	N/A	340	376	368	COI: JN248781		Clade VII-B monophyly shown [81]
Faviidae	*Montastraea cavernosa*	Yes	LC	ED	395	104	97	12S: EF597006; COI: AB117288; CYB: AB117373	[68]	
Faviidae	*Montastraea colemani*	Yes	NT	N/A	288	238	235	COI: HQ203284		Clade VII-F monophyly shown [81]
Faviidae	*Montastraea curta*	Yes	LC	N/A	538	312	310	COI: AB117278; CYB: AB117359		
Faviidae	*Montastraea faveolata*	Yes	EN	N/A	2	7	21	12S: AP008977; 16S: AP008977; AT6: AP008977; COI: AP008977; CYB: AP008977		
Faviidae	*Montastraea franksi*	Yes	VU	N/A	36	34	28	12S: AP008976; 16S: AP008976; AT6: AP008976; COI: AP008976; CYB: AP008976		
Faviidae	*Montastraea magnistellata*	Yes	NT	N/A	326	333	331	COI: AB117279; CYB: AB117360		Clade VII-F monophyly shown [81]
Faviidae	*Montastraea multipunctata*	Yes	VU	N/A	38	37	32	COI: HQ203289		
Faviidae	*Montastraea salebrosa*	Yes	VU	N/A	92	149	139	COI: HQ203290		
Faviidae	*Montastraea serageldini*	Yes	VU	N/A	49	59	54			Closest to *M. curta* [93]
Faviidae	*Montastraea valenciennesi*	Yes	NT	N/A	307	283	281	12S: AF333061; COI: AB117280; CYB: AB117361		Clade VII-F monophyly shown [81]
Faviidae	*Moseleya latistellata*	Yes	VU	12	30	24	17	COI: HQ203293		

Family	Species	Reef	Red List	EoE rank	EDGE rank (IUCN100)	EDGE rank (Isaac)	EDGE rank (Pessimistic)	Molecular sources	Morphological sources	Remarks
Faviidae	*Oulastrea crispata*	Yes	LC	ED	224	2	1	12S: AF333062; COI: AB441197; CYB: AB441282		
Faviidae	*Oulophyllia bennettae*	Yes	NT	N/A	350	391	383	COI: AB117277; CYB: AB117358		
Faviidae	*Oulophyllia crispa*	Yes	NT	N/A	351	392	384	COI: AB117276; CYB: AB117357		
Faviidae	*Oulophyllia levis*	Yes	LC	N/A	725	609	609			Closest to *O. crispa* [92]
Faviidae	*Parasimplastrea sheppardi*	Yes	EN	5	10	42	125			Closest to *Leptastrea* [93]
Faviidae	*Platygyra acuta*	Yes	NT	N/A	319	316	314	COI: JN248782	[64]	
Faviidae	*Platygyra carnosus*	Yes	NT	N/A	375	437	429		[64]	
Faviidae	*Platygyra contorta*	Yes	LC	N/A	681	509	506	COI: JN248783	[64]	
Faviidae	*Platygyra crosslandi*	Yes	NT	N/A	375	437	429		[64]	
Faviidae	*Platygyra daedalea*	Yes	LC	N/A	527	295	294	COI: AB117281; CYB: AB117362	[64,68]	
Faviidae	*Platygyra lamellina*	Yes	NT	N/A	317	314	312	COI: HQ203302; CYB: AB117363	[64]	
Faviidae	*Platygyra pini*	Yes	LC	N/A	513	281	279	COI: HQ203303	[64]	
Faviidae	*Platygyra ryukyuensis*	Yes	NT	N/A	319	316	314	COI: HQ203304	[64]	
Faviidae	*Platygyra sinensis*	Yes	LC	N/A	755	649	649	12S: AF177047; COI: HQ203305	[64]	
Faviidae	*Platygyra verweyi*	Yes	NT	N/A	317	314	312	COI: EU371722	[64]	*Platygyra* cf. *verweyi* in GenBank
Faviidae	*Platygyra yaeyamaensis*	Yes	VU	N/A	143	266	264		[64]	
Faviidae	*Plesiastrea devantieri*	Yes	NT	ED	404	512	509	COI: FR837987		
Faviidae	*Plesiastrea versipora*	Yes	LC	ED	418	129	119	COI: AB289561; CYB: AB289566		
Faviidae	*Solenastrea bournoni*	Yes	LC	N/A	487	254	252	COI: AB117291; CYB: AB117376		
Faviidae	*Solenastrea hyades*	Yes	LC	N/A	429	150	140	COI: FJ966870		
Flabellidae	*Flabellum alabastrum*	No	N/A	N/A	N/A	N/A	N/A		[64]	
Flabellidae	*Flabellum angulare*	No	N/A	N/A	N/A	N/A	N/A	16S: AF550363	[64]	
Flabellidae	*Flabellum angustum*	No	N/A	N/A	N/A	N/A	N/A		[64]	
Flabellidae	*Flabellum aotearoa*	No	N/A	N/A	N/A	N/A	N/A		[64]	
Flabellidae	*Flabellum apertum*	No	N/A	N/A	N/A	N/A	N/A	COI: HM018635	[64]	
Flabellidae	*Flabellum arcuatile*	No	N/A	N/A	N/A	N/A	N/A	COI: HM018636	[64]	
Flabellidae	*Flabellum areum*	No	N/A	N/A	N/A	N/A	N/A		[64]	
Flabellidae	*Flabellum atlanticum*	No	N/A	N/A	N/A	N/A	N/A		[64]	
Flabellidae	*Flabellum australe*	No	N/A	N/A	N/A	N/A	N/A		[64]	
Flabellidae	*Flabellum campanulatum*	No	N/A	N/A	N/A	N/A	N/A		[64]	
Flabellidae	*Flabellum chunii*	No	N/A	N/A	N/A	N/A	N/A		[64]	
Flabellidae	*Flabellum conuis*	No	N/A	N/A	N/A	N/A	N/A		[64]	
Flabellidae	*Flabellum curvatum*	No	N/A	N/A	N/A	N/A	N/A		[64]	
Flabellidae	*Flabellum daphnense*	No	DD	N/A	N/A	N/A	N/A		[64]	
Flabellidae	*Flabellum deludens*	No	N/A	N/A	N/A	N/A	N/A	16S: AB510170; COI: HM018638	[64]	
Flabellidae	*Flabellum flexuosum*	No	N/A	N/A	N/A	N/A	N/A		[64]	
Flabellidae	*Flabellum floridanum*	No	N/A	N/A	N/A	N/A	N/A		[64]	
Flabellidae	*Flabellum folkesoni*	No	N/A	N/A	N/A	N/A	N/A	COI: HM018639	[64]	
Flabellidae	*Flabellum gardineri*	No	N/A	N/A	N/A	N/A	N/A		[64]	
Flabellidae	*Flabellum hoffmeisteri*	No	N/A	N/A	N/A	N/A	N/A		[64]	
Flabellidae	*Flabellum impensum*	No	N/A	N/A	N/A	N/A	N/A	16S: AF265582	[64]	
Flabellidae	*Flabellum japonicum*	No	N/A	N/A	N/A	N/A	N/A	16S: AB510178	[64]	
Flabellidae	*Flabellum knoxi*	No	N/A	N/A	N/A	N/A	N/A		[64]	
Flabellidae	*Flabellum lamellulosum*	No	N/A	N/A	N/A	N/A	N/A	COI: HM018640	[64]	
Flabellidae	*Flabellum lowekeyesi*	No	N/A	N/A	N/A	N/A	N/A	COI: HM018641	[64]	
Flabellidae	*Flabellum macandrewi*	No	N/A	N/A	N/A	N/A	N/A		[64]	
Flabellidae	*Flabellum magnificum*	No	N/A	N/A	N/A	N/A	N/A	16S: AB510167; COI: HM018637	[64]	*Flabellum* cf. *magnificum* in GenBank
Flabellidae	*Flabellum marcus*	No	N/A	N/A	N/A	N/A	N/A		[64]	
Flabellidae	*Flabellum marenzelleri*	No	N/A	N/A	N/A	N/A	N/A		[64]	
Flabellidae	*Flabellum messum*	No	N/A	N/A	N/A	N/A	N/A		[64]	
Flabellidae	*Flabellum moseleyi*	No	N/A	N/A	N/A	N/A	N/A		[64]	
Flabellidae	*Flabellum ongulense*	No	N/A	N/A	N/A	N/A	N/A		[64]	
Flabellidae	*Flabellum patens*	No	N/A	N/A	N/A	N/A	N/A		[64]	
Flabellidae	*Flabellum pavoninum*	No	N/A	N/A	N/A	N/A	N/A	16S: AB510168	[64]	
Flabellidae	*Flabellum politum*	No	N/A	N/A	N/A	N/A	N/A		[64]	
Flabellidae	*Flabellum sexcostatum*	No	N/A	N/A	N/A	N/A	N/A		[64]	
Flabellidae	*Flabellum sibogae*	No	N/A	N/A	N/A	N/A	N/A		[64]	
Flabellidae	*Flabellum thouarsii*	No	N/A	N/A	N/A	N/A	N/A		[64]	
Flabellidae	*Flabellum transversale*	No	N/A	N/A	N/A	N/A	N/A		[64]	
Flabellidae	*Flabellum tuthilli*	No	N/A	N/A	N/A	N/A	N/A	COI: HM018643	[64]	
Flabellidae	*Flabellum vaughani*	No	N/A	N/A	N/A	N/A	N/A	COI: HM018644	[64]	
Flabellidae	*Javania exserta*	No	N/A	N/A	N/A	N/A	N/A	COI: HM018651		
Flabellidae	*Javania fusca*	No	N/A	N/A	N/A	N/A	N/A	COI: HM018652		
Flabellidae	*Javania insignis*	No	N/A	N/A	N/A	N/A	N/A	16S: AB510174		
Flabellidae	*Javania lamprotichum*	No	N/A	N/A	N/A	N/A	N/A	COI: HM018653		
Flabellidae	*Monomyces pygmaea*	No	N/A	N/A	N/A	N/A	N/A	16S: AF265583	[64]	
Flabellidae	*Monomyces rubrum*	No	N/A	N/A	N/A	N/A	N/A		[64]	
Flabellidae	*Placotrochides scaphula*	No	N/A	N/A	N/A	N/A	N/A	COI: HM018661		

Family	Species	Reef	Red List	EoE rank	EDGE rank (IUCN100)	EDGE rank (Isaac)	EDGE rank (Pessimistic)	Molecular sources	Morphological sources	Remarks
Flabellidae	*Placotrochus laevis*	No	N/A	N/A	N/A	N/A	N/A	16S: AF265589	[64]	
Flabellidae	*Rhizotrochus typus*	No	N/A	N/A	N/A	N/A	N/A	16S: AB510175		
Flabellidae	*Truncatoflabellum australiensis*	No	N/A	N/A	N/A	N/A	N/A	COI: HM018670		
Flabellidae	*Truncatoflabellum candeanum*	No	N/A	N/A	N/A	N/A	N/A	COI: HM018671		
Flabellidae	*Truncatoflabellum macroeschara*	No	N/A	N/A	N/A	N/A	N/A	COI: HM018672		
Flabellidae	*Truncatoflabellum spheniscus*	No	N/A	N/A	N/A	N/A	N/A	16S: AB510172		
Fungiacyathidae	*Fungiacyathus fragilis*	No	N/A	N/A	N/A	N/A	N/A	COI: HM018645		
Fungiacyathidae	*Fungiacyathus marenzelleri*	No	N/A	N/A	N/A	N/A	N/A	12S: EF597074; 16S: L76004		
Fungiacyathidae	*Fungiacyathus pusillus*	No	N/A	N/A	N/A	N/A	N/A	COI: HM018646		
Fungiacyathidae	*Fungiacyathus stephanus*	No	N/A	N/A	N/A	N/A	N/A	12S: JF825138; 16S: JF825138; AT6: JF825138; COI: JF825138; CYB: JF825138; ND5: JF825138		
Fungiacyathidae	*Fungiacyathus turbinolioides*	No	N/A	N/A	N/A	N/A	N/A	COI: HM018648		
Fungiidae	*Cantharellus doederleini*	Yes	LC	N/A	748	642	642		[65]	
Fungiidae	*Cantharellus jebbi*	Yes	LC	N/A	748	642	642			*Cantharellus* monophyly assumed
Fungiidae	*Cantharellus noumeae*	Yes	EN	N/A	22	114	218		[65]	
Fungiidae	*Ctenactis albitentaculata*	Yes	NT	N/A	269	161	153	COI: EU149869	[65]	
Fungiidae	*Ctenactis crassa*	Yes	LC	N/A	548	328	326	COI: EU149859	[65]	
Fungiidae	*Ctenactis echinata*	Yes	LC	N/A	546	326	324	COI: EU149879	[65]	
Fungiidae	*Cycloseris costulata*	Yes	LC	N/A	510	276	274	COI: EU149870	[65,68]	Revised from *Fungia costulata* [79]
Fungiidae	*Cycloseris curvata*	Yes	VU	N/A	141	257	255		[65]	Revised from *Fungia curvata* [79]
Fungiidae	*Cycloseris cyclolites*	Yes	LC	N/A	506	271	269	COI: EU202719	[65]	Revised from *Fungia cyclolites* [79]
Fungiidae	*Cycloseris distorta*	Yes	LC	N/A	748	642	642		[65]	Revised from *Fungia distorta* [79]
Fungiidae	*Cycloseris fragilis*	Yes	LC	N/A	507	272	270	16S: L75998; COI: EU149860	[65]	Revised from *Fungia fragilis* [79]
Fungiidae	*Cycloseris hexagonalis*	Yes	LC	N/A	748	642	642		[65]	Revised from *Fungia hexagonalis* [79]
Fungiidae	*Cycloseris mokai*	Yes	LC	N/A	504	268	266	COI: EU149877	[65,68]	Revised from *Lithophyllon mokai* [79]
Fungiidae	*Cycloseris sinensis*	Yes	LC	N/A	505	270	268	COI: EU149900	[65,68]	Revised from *Fungia sinensis* [79]
Fungiidae	*Cycloseris somervillei*	Yes	LC	N/A	748	642	642		[65]	Revised from *Fungia somervillei* [79]
Fungiidae	*Cycloseris tenuis*	Yes	LC	N/A	511	277	275	COI: EU149871	[65]	Revised from *Fungia tenuis* [79]
Fungiidae	*Cycloseris vaughani*	Yes	LC	N/A	508	273	271	16S: L75999; COI: EU149861	[65]	Revised from *Fungia vaughani* [79]
Fungiidae	*Danafungia horrida*	Yes	LC	N/A	693	556	556		[65]	Revised from *Fungia horrida* [79]
Fungiidae	*Danafungia scruposa*	Yes	LC	N/A	398	111	103	COI: EU149872	[65]	Revised from *Fungia scruposa* [79]
Fungiidae	*Fungia fungites*	Yes	NT	N/A	140	23	16	COI: EU149892	[64,65,68]	
Fungiidae	*Halomitra clavator*	Yes	VU	N/A	21	12	8	COI: EU149904	[65]	
Fungiidae	*Halomitra pileus*	Yes	LC	N/A	379	82	77	COI: EU149865	[65,68]	
Fungiidae	*Heliofungia actiniformis*	Yes	VU	3	16	9	5	12S: EF596995; COI: EU149885	[65]	
Fungiidae	*Heliofungia fralinae*	Yes	LC	N/A	394	103	96	COI: EU149901	[65]	Revised from *Fungia fralinae* [79]
Fungiidae	*Herpolitha limax*	Yes	LC	N/A	460	195	187	COI: AB441223; CYB: AB441308	[65,68]	
Fungiidae	*Lithophyllon concinna*	Yes	LC	N/A	427	147	137	COI: EU149893	[65,68]	Revised from *Fungia concinna* [79]
Fungiidae	*Lithophyllon puishani*	Yes	DD	N/A	301	190	182			Revised from *Fungia puishani*; closest to *L. scabra* [93]
Fungiidae	*Lithophyllon ranjithi*	Yes	EN	N/A	7	17	41			*Lithophyllon* monophyly shown [79]
Fungiidae	*Lithophyllon repanda*	Yes	LC	N/A	428	148	138	COI: EU149883	[65,68]	Revised from *Fungia repanda* [79]
Fungiidae	*Lithophyllon scabra*	Yes	LC	N/A	455	187	179	COI: EU149874	[65]	Revised from *Fungia scabra* [79]
Fungiidae	*Lithophyllon spinifer*	Yes	LC	N/A	419	130	120	COI: EU149864	[65]	Revised from *Fungia spinifer* [79]
Fungiidae	*Lithophyllon undulatum*	Yes	NT	N/A	172	48	44	COI: EU149867	[65]	
Fungiidae	*Lobactis scutaria*	Yes	LC	N/A	298	22	15	12S: DQ320497; 16S: L76005; COI: AB441224; CYB: AB441309	[65]	Revised from *Fungia scutaria* [79]
Fungiidae	*Pleuractis granulosa*	Yes	LC	N/A	472	212	206	COI: EU149884	[65,66]	Revised from *Fungia granulosa* [79]
Fungiidae	*Pleuractis gravis*	Yes	DD	N/A	305	199	192	COI: EU149910	[65,66]	Previously excluded [11]; revised from *Fungia gravis* [79]

Family	Species	Reef	Red List	EoE rank	EDGE rank (IUCN100)	EDGE rank (Isaac)	EDGE rank (Pessimistic)	Molecular sources	Morphological sources	Remarks
Fungiidae	*Pleuractis moluccensis*	Yes	LC	N/A	501	262	260	COI: EU149909	[65,66]	Revised from *Fungia moluccensis* [79]
Fungiidae	*Pleuractis paumotensis*	Yes	LC	N/A	620	450	442	COI: EU149911	[65,66]	Revised from *Fungia paumotensis* [79]
Fungiidae	*Pleuractis seychellensis*	Yes	VU	N/A	84	126	116		[66]	Revised from *Fungia seychellensis* [79]
Fungiidae	*Pleuractis taiwanensis*	Yes	VU	N/A	56	68	63		[66]	Revised from *Fungia taiwanensis* [79]
Fungiidae	*Podabacia crustacea*	Yes	LC	N/A	583	396	388	COI: EU149878	[65]	
Fungiidae	*Podabacia kunzmanni*	Yes	DD	N/A	366	346	344	COI: EU149908		New species [46]
Fungiidae	*Podabacia motuporensis*	Yes	NT	N/A	238	132	121	COI: EU149868		
Fungiidae	*Podabacia sinai*	Yes	DD	N/A	366	346	344	COI: EU149888		
Fungiidae	*Polyphyllia novaehiberniae*	Yes	NT	N/A	312	303	302		[65]	
Fungiidae	*Polyphyllia talpina*	Yes	LC	N/A	675	478	472	COI: EU149915	[65,68]	
Fungiidae	*Sandalolitha dentata*	Yes	LC	N/A	581	394	386	COI: EU149914	[65]	
Fungiidae	*Sandalolitha robusta*	Yes	LC	N/A	582	395	387	COI: EU149917	[65]	
Fungiidae	*Zoopilus echinatus*	Yes	LC	N/A	621	451	443	12S: EF596990; 16S: L76024; COI: EU149916	[64,65,68]	
Gardineriidae	*Gardineria hawaiiensis*	No	N/A	N/A	N/A	N/A	N/A	12S: GQ868660; 16S: GQ868701; COI: GQ868678		
Gardineriidae	*Gardineria paradoxa*	No	N/A	N/A	N/A	N/A	N/A	12S: GQ868656; 16S: GQ868700; COI: GQ868681		
Guyniidae	*Guynia annulata*	No	N/A	N/A	N/A	N/A	N/A	16S: AF265580		
Meandrinidae	*Ctenella chagius*	Yes	EN	9	1	1	10	COI: AB441208; CYB: AB441293		
Meandrinidae	*Dendrogyra cylindrus*	Yes	VU	2	50	60	55	12S: EF597024; COI: AB117299; CYB: AB117384		
Meandrinidae	*Dichocoenia stellaris*	Yes	DD	N/A	346	304	303		[64]	
Meandrinidae	*Dichocoenia stokesi*	Yes	VU	7	60	73	68	12S: EF597020; 16S: AF265607; COI: AB117298; CYB: AB117383	[64]	
Meandrinidae	*Eusmilia fastigiata*	Yes	LC	N/A	550	331	329	COI: AB117294; CYB: AB117380		
Meandrinidae	*Gyrosmilia interrupta*	Yes	LC	N/A	571	370	362			Closest to *Ctenella chagius* [92]
Meandrinidae	*Meandrina braziliensis*	Yes	DD	N/A	296	173	165	COI: AB117297; CYB: AB117382	[68]	
Meandrinidae	*Meandrina danae*	Yes	LC	N/A	576	380	372			Closest to *M. braziliensis* [87]
Meandrinidae	*Meandrina meandrites*	Yes	LC	N/A	499	260	258	12S: EF597032; COI: AB117296; CYB: AB117381		
Meandrinidae	*Montigyra kenti*	Yes	DD	N/A	329	258	256			Closest to *Gyrosmilia* [90]
Merulinidae	*Boninastrea boninensis*	Yes	DD	N/A	426	453	445			Closest to *Merulina* [90]
Merulinidae	*Hydnophora bonsai*	Yes	EN	N/A	23	121	232		[64]	
Merulinidae	*Hydnophora exesa*	Yes	NT	N/A	303	275	273	12S: AF333059; COI: AB117285; CYB: AB117370	[64]	
Merulinidae	*Hydnophora grandis*	Yes	LC	N/A	480	248	245	COI: AB117286; CYB: AB117371	[64]	
Merulinidae	*Hydnophora microconos*	Yes	NT	N/A	199	100	93	COI: HQ203277	[64]	
Merulinidae	*Hydnophora pilosa*	Yes	LC	N/A	481	249	246	COI: HQ203278	[64]	
Merulinidae	*Hydnophora rigida*	Yes	LC	N/A	729	615	615	12S: EF597000; 16S: L76009	[64]	
Merulinidae	*Merulina ampliata*	Yes	LC	N/A	680	490	484	12S: AF333058; COI: AB117283; CYB: AB117368		
Merulinidae	*Merulina scabricula*	Yes	LC	N/A	710	581	581	16S: L76014; COI: AB117284; CYB: AB117369		
Merulinidae	*Merulina scheeri*	Yes	LC	N/A	690	540	540			*Merulina* monophyly shown [81]; closest to *M. ampliata* [80]
Merulinidae	*Paraclavarina triangularis*	Yes	NT	N/A	339	375	367			Closest to *Merulina* [89]
Merulinidae	*Scapophyllia cylindrica*	Yes	LC	N/A	694	557	557	COI: AB441198; CYB: AB441283		
Micrabaciidae	*Leptopenus antarcticus*	No	N/A	N/A	N/A	N/A	N/A		[68]	
Micrabaciidae	*Letepsammia formosissima*	No	N/A	N/A	N/A	N/A	N/A	12S: GQ868663; 16S: GQ868697; COI: GQ868685		
Micrabaciidae	*Rhombopsammia niphada*	No	N/A	N/A	N/A	N/A	N/A	12S: GQ868661; 16S: GQ868693; COI: GQ868683		
Micrabaciidae	*Stephanophyllia complicata*	No	N/A	N/A	N/A	N/A	N/A	16S: GQ868689		
Mussidae	*Acanthastrea bowerbanki*	Yes	VU	N/A	130	230	226		[67]	
Mussidae	*Acanthastrea brevis*	Yes	VU	N/A	130	230	226		[67]	
Mussidae	*Acanthastrea echinata*	Yes	LC	N/A	691	554	554	COI: AB117249; CYB: AB117327	[61,67]	
Mussidae	*Acanthastrea faviaformis*	Yes	VU	N/A	130	230	226		[67]	

Family	Species	Reef	Red List	EoE rank	EDGE rank (IUCN100)	EDGE rank (Isaac)	EDGE rank (Pessimistic)	Molecular sources	Morphological sources	Remarks
Mussidae	*Acanthastrea hemprichii*	Yes	VU	N/A	130	230	226		[67]	
Mussidae	*Acanthastrea hillae*	Yes	NT	ED	171	47	43	COI: AB441199; CYB: AB441284		
Mussidae	*Acanthastrea ishigakiensis*	Yes	VU	N/A	130	230	226		[67]	
Mussidae	*Acanthastrea lordhowensis*	Yes	NT	N/A	355	405	397		[67]	
Mussidae	*Acanthastrea maxima*	Yes	NT	N/A	355	405	397		[67]	
Mussidae	*Acanthastrea regularis*	Yes	VU	N/A	130	230	226		[67]	
Mussidae	*Acanthastrea rotundoflora*	Yes	NT	N/A	327	336	334	COI: AB117251; CYB: AB117328	[61,67]	
Mussidae	*Acanthastrea subechinata*	Yes	NT	N/A	355	405	397		[67]	
Mussidae	*Australomussa rowleyensis*	Yes	NT	N/A	365	421	413		[67]	
Mussidae	*Blastomussa merleti*	Yes	LC	ED	412	128	118		[67]	
Mussidae	*Blastomussa wellsi*	Yes	NT	ED	86	5	3	COI: AB289563; CYB: AB289565	[67]	
Mussidae	*Cynarina lacrymalis*	Yes	NT	N/A	168	41	36	12S: EF597034; COI: AB117246; CYB: AB117323	[67]	
Mussidae	*Indophyllia macassarensis*	Yes	DD	N/A	314	241	238			Closest to *Cynarina lacrymalis* [73]
Mussidae	*Isophyllastrea rigida*	Yes	LC	N/A	698	561	561		[61,67]	
Mussidae	*Isophyllia sinuosa*	Yes	LC	N/A	672	475	469	COI: AB117238; CYB: AB117315	[61,67]	
Mussidae	*Lobophyllia corymbosa*	Yes	LC	N/A	600	426	418	COI: AB117241; CYB: AB117318	[64,67]	
Mussidae	*Lobophyllia dentatus*	Yes	VU	N/A	120	216	210		[64,67]	
Mussidae	*Lobophyllia diminuta*	Yes	VU	N/A	120	216	210		[64,67]	
Mussidae	*Lobophyllia flabelliformis*	Yes	VU	N/A	120	216	210		[64,67]	
Mussidae	*Lobophyllia hataii*	Yes	LC	N/A	723	607	607		[64,67]	
Mussidae	*Lobophyllia hemprichii*	Yes	LC	N/A	577	385	377	12S: EF597013; 16S: L76013; COI: AB117240; CYB: AB117317	[64,67,68]	
Mussidae	*Lobophyllia pachysepta*	Yes	NT	N/A	280	206	200	COI: AB117242; CYB: AB117319	[64,67]	
Mussidae	*Lobophyllia robusta*	Yes	LC	N/A	723	607	607		[64,67]	
Mussidae	*Lobophyllia serratus*	Yes	EN	N/A	15	86	189		[64,67]	
Mussidae	*Micromussa amakusensis*	Yes	NT	ED	198	95	89	COI: AB441200; CYB: AB441285		
Mussidae	*Micromussa diminuta*	Yes	DD	N/A	295	170	162			*Micromussa* monophyly assumed; closest to *M. minuta* [93]
Mussidae	*Micromussa minuta*	Yes	NT	N/A	227	124	114			*Micromussa* monophyly assumed
Mussidae	*Mussa angulosa*	Yes	LC	N/A	664	455	447	12S: DQ643834; 16S: DQ643834; AT6: DQ643834; COI: DQ643834; CYB: DQ643834	[61,67]	
Mussidae	*Mussismilia braziliensis*	Yes	DD	Potential	443	486	480	COI: AB117231; CYB: AB117309	[61,67,68]	
Mussidae	*Mussismilia harttii*	Yes	DD	N/A	444	487	481	COI: AB117232; CYB: AB117308	[61,67,68]	
Mussidae	*Mussismilia hispida*	Yes	DD	N/A	442	485	479	COI: AB117233; CYB: AB117310	[61,67,68]	
Mussidae	*Mycetophyllia aliciae*	Yes	LC	N/A	537	309	308	12S: EF597039; COI: AB117235; CYB: AB117312	[61,67]	
Mussidae	*Mycetophyllia danaana*	Yes	LC	N/A	695	558	558	COI: AB117234; CYB: AB117311	[61,67]	
Mussidae	*Mycetophyllia ferox*	Yes	VU	N/A	94	154	144		[61,67]	
Mussidae	*Mycetophyllia lamarckiana*	Yes	LC	N/A	458	193	185	12S: EF597040	[61,67]	
Mussidae	*Mycetophyllia reesi*	Yes	DD	N/A	358	335	333		[61,67]	
Mussidae	*Scolymia australis*	Yes	LC	N/A	671	474	468		[67]	
Mussidae	*Scolymia cubensis*	Yes	LC	N/A	696	559	559	COI: AB117236; CYB: AB117314	[61]	Atlantic *Scolymia*
Mussidae	*Scolymia lacera*	Yes	LC	N/A	697	560	560		[61]	Junior synonym of *S. cubensis* [92]; Atlantic *Scolymia*
Mussidae	*Scolymia vitiensis*	Yes	NT	N/A	265	157	146	COI: AB117247; CYB: AB117324	[67]	
Mussidae	*Scolymia wellsi*	Yes	DD	N/A	411	425	417		[61,68]	Junior synonym of *S. cubensis* [78]; Atlantic *Scolymia*
Mussidae	*Symphyllia agaricia*	Yes	LC	N/A	702	564	564	COI: AB117243; CYB: AB117320	[67]	
Mussidae	*Symphyllia erythraea*	Yes	LC	N/A	703	565	565		[67]	
Mussidae	*Symphyllia hassi*	Yes	VU	N/A	110	186	178		[67]	
Mussidae	*Symphyllia radians*	Yes	LC	N/A	700	562	562	COI: AB117245; CYB: AB117322	[67]	
Mussidae	*Symphyllia recta*	Yes	LC	N/A	701	563	563	COI: AB117244; CYB: AB117321	[67]	
Mussidae	*Symphyllia valenciennesii*	Yes	LC	N/A	578	386	378	COI: HM018666	[67]	
Mussidae	*Symphyllia wilsoni*	Yes	LC	N/A	703	565	565		[67]	
Oculinidae	*Cyathelia axillaris*	No	N/A	N/A	N/A	N/A	N/A	COI: HM018622		

Family	Species	Reef	Red List	EoE rank	EDGE rank (IUCN100)	EDGE rank (Isaac)	EDGE rank (Pessimistic)	Molecular sources	Morphological sources	Remarks
Oculinidae	*Galaxea acrhelia*	Yes	VU	N/A	78	97	91			Closest to *G. horrescens* [93]
Oculinidae	*Galaxea astreata*	Yes	VU	N/A	55	67	62	12S: AF333056		
Oculinidae	*Galaxea cryptoramosa*	Yes	VU	N/A	73	88	82			Closest to *G. astreata* [93]
Oculinidae	*Galaxea fascicularis*	Yes	NT	N/A	304	278	276	16S: L76006; COI: AB441201; CYB: AB441286; ND5: AB109376		
Oculinidae	*Galaxea horrescens*	Yes	LC	N/A	594	416	408	12S: EF597096; 16S: L75994		
Oculinidae	*Galaxea longisepta*	Yes	NT	N/A	293	242	239			Closest to *G. horrescens* [93]
Oculinidae	*Galaxea paucisepta*	Yes	NT	N/A	285	220	215			Closest to *G. astreata* [75]
Oculinidae	*Madrepora oculata*	No	N/A	N/A	N/A	N/A	N/A	16S: AF550369; COI: HM018659		
Oculinidae	*Oculina diffusa*	Yes	LC	N/A	585	408	400	COI: AB117293; CYB: AB117379	[64]	
Oculinidae	*Oculina patagonica*	Yes	LC	N/A	764	661	661	12S: EF597025; 16S: AF265601	[64,68]	
Oculinidae	*Oculina profunda*	No	N/A	N/A	N/A	N/A	N/A		[64]	
Oculinidae	*Oculina robusta*	Yes	DD	N/A	325	253	250	COI: FJ966869	[64]	
Oculinidae	*Oculina tenella*	Yes	DD	N/A	473	590	590		[64]	
Oculinidae	*Oculina valenciennesi*	Yes	DD	N/A	473	590	590		[64]	
Oculinidae	*Oculina varicosa*	Yes	VU	11	88	139	129	COI: FJ966875	[64]	
Oculinidae	*Oculina virgosa*	No	N/A	N/A	N/A	N/A	N/A		[64]	
Oculinidae	*Schizoculina africana*	Yes	DD	Potential	475	592	592			*Schizoculina* monophyly assumed; closest to *Oculina* [95]
Oculinidae	*Schizoculina fissipara*	Yes	DD	Potential	475	592	592			*Schizoculina* monophyly assumed; closest to *Oculina* [95]
Oculinidae	*Simplastrea vesicularis*	Yes	DD	Potential	381	374	366			Closest to *Galaxea* [92]
Pectiniidae	*Echinomorpha nishihirai*	Yes	NT	N/A	337	366	358			Closest to *Echinophyllia* [91]
Pectiniidae	*Echinophyllia aspera*	Yes	LC	N/A	568	364	356	COI: AB117252; CYB: AB117329		
Pectiniidae	*Echinophyllia costata*	Yes	VU	N/A	95	155	145			*Echinophyllia* monophyly shown [42]; closest to *E. pectinata* [93]
Pectiniidae	*Echinophyllia echinata*	Yes	LC	N/A	679	483	477			*Echinophyllia* monophyly shown [42]
Pectiniidae	*Echinophyllia echinoporoides*	Yes	LC	N/A	586	409	401	COI: AB117254; CYB: AB117331		
Pectiniidae	*Echinophyllia orpheensis*	Yes	LC	N/A	549	329	327	12S: AF333065; COI: AB117253; CYB: AB117330		
Pectiniidae	*Echinophyllia patula*	Yes	LC	N/A	608	434	426			*Echinophyllia* monophyly shown [42]; closest to *E. aspera* [92]
Pectiniidae	*Echinophyllia pectinata*	Yes	DD	N/A	388	381	373			*Echinophyllia* monophyly shown [42]
Pectiniidae	*Mycedium elephantotus*	Yes	LC	N/A	728	612	612	12S: AF333057; COI: AB117387; CYB: AB117366		
Pectiniidae	*Mycedium mancaoi*	Yes	LC	N/A	753	647	647			*Mycedium* monophyly shown [81]; closest to *M. elephantotus* [92]
Pectiniidae	*Mycedium robokaki*	Yes	LC	N/A	758	652	652	COI: HQ203295		
Pectiniidae	*Mycedium spina*	Yes	DD	N/A	457	552	552			*Mycedium* monophyly shown [81]
Pectiniidae	*Mycedium steeni*	Yes	VU	N/A	146	279	277			*Mycedium* monophyly shown [81]; closest to *M. robokaki* [93]
Pectiniidae	*Mycedium umbra*	Yes	LC	N/A	753	647	647			*Mycedium* monophyly shown [81]; closest to *M. elephantotus* [93]
Pectiniidae	*Oxypora convoluta*	Yes	DD	N/A	386	379	371			*Oxypora* monophyly assumed; closest to *O. lacera* [93]
Pectiniidae	*Oxypora crassispinosa*	Yes	LC	N/A	715	596	596			*Oxypora* monophyly assumed; closest to *O. glabra* [92]
Pectiniidae	*Oxypora egyptensis*	Yes	DD	N/A	425	452	444			*Oxypora* monophyly assumed; closest to *O. glabra* [92]
Pectiniidae	*Oxypora glabra*	Yes	LC	N/A	715	596	596			*Oxypora* monophyly assumed
Pectiniidae	*Oxypora lacera*	Yes	LC	N/A	539	313	311	COI: AB117255; CYB: AB117332		
Pectiniidae	*Pectinia africanus*	Yes	VU	N/A	150	288	287			*Pectinia* + *Mycedium* monophyly shown [81]; closest to *P. lactuca* [93]
Pectiniidae	*Pectinia alcicornis*	Yes	VU	N/A	148	286	285	12S: EF597037; 16S: L76017; COI: AB117385; CYB: AB117364		
Pectiniidae	*Pectinia ayleni*	Yes	NT	N/A	382	465	457	COI: HQ203299		
Pectiniidae	*Pectinia crassa*	Yes	DD	N/A	465	575	575			*Pectinia* + *Mycedium* monophyly shown [81]
Pectiniidae	*Pectinia elongata*	Yes	NT	N/A	383	466	458			*Pectinia* + *Mycedium* monophyly shown [81]
Pectiniidae	*Pectinia lactuca*	Yes	VU	N/A	149	287	286	COI: HQ203300		
Pectiniidae	*Pectinia maxima*	Yes	EN	N/A	24	131	251			*Pectinia* + *Mycedium* monophyly shown [81]; closest to *P. lactuca* & *P. ayleni* [92]
Pectiniidae	*Pectinia paeonia*	Yes	NT	N/A	308	285	284	COI: AB117386; CYB: AB117365	[68]	

Family	Species	Reef	Red List	EoE rank	EDGE rank (IUCN100)	EDGE rank (Isaac)	EDGE rank (Pessimistic)	Molecular sources	Morphological sources	Remarks
Pectiniidae	*Pectinia pygmaeus*	Yes	NT	N/A	383	466	458			*Pectinia* + *Mycedium* monophyly shown [81]; closest to *P. elongata* & *P. teres* [93]
Pectiniidae	*Pectinia teres*	Yes	NT	N/A	383	466	458			*Pectinia* + *Mycedium* monophyly shown [81]; closest to *P. elongata* [92]
Pocilloporidae	*Pocillopora ankeli*	Yes	VU	N/A	44	50	47		[69]	
Pocilloporidae	*Pocillopora capitata*	Yes	LC	N/A	558	340	338		[69]	
Pocilloporidae	*Pocillopora damicornis*	Yes	LC	N/A	518	290	289	12S: EF526302; 16S: EF526302; AT6: EF526302; COI: EF526302; CYB: EF526302	[69]	
Pocilloporidae	*Pocillopora danae*	Yes	VU	N/A	44	50	47		[69]	
Pocilloporidae	*Pocillopora effusus*	Yes	DD	N/A	315	246	243		[69]	
Pocilloporidae	*Pocillopora elegans*	Yes	VU	N/A	44	50	47		[69]	
Pocilloporidae	*Pocillopora eydouxi*	Yes	NT	N/A	200	102	95	12S: EF526303; 16S: EF526303; AT6: EF526303; COI: EF526303; CYB: EF526303	[69]	
Pocilloporidae	*Pocillopora fungiformis*	Yes	EN	N/A	6	15	37		[69]	
Pocilloporidae	*Pocillopora indiania*	Yes	VU	N/A	44	50	47		[69]	
Pocilloporidae	*Pocillopora inflata*	Yes	VU	N/A	44	50	47		[69]	
Pocilloporidae	*Pocillopora kelleheri*	Yes	LC	N/A	558	340	338		[69]	
Pocilloporidae	*Pocillopora ligulata*	Yes	LC	N/A	558	340	338		[69]	
Pocilloporidae	*Pocillopora meandrina*	Yes	LC	N/A	557	339	337	12S: EF596976; 16S: L76018	[69]	
Pocilloporidae	*Pocillopora molokensis*	Yes	DD	N/A	315	246	243		[69]	
Pocilloporidae	*Pocillopora setichelli*	Yes	LC	N/A	558	340	338		[69]	
Pocilloporidae	*Pocillopora verrucosa*	Yes	LC	N/A	424	143	133	COI: AB441230; CYB: AB441315	[69]	
Pocilloporidae	*Pocillopora woodjonesi*	Yes	LC	N/A	558	340	338		[69]	
Pocilloporidae	*Pocillopora zelli*	Yes	LC	N/A	558	340	338		[69]	
Pocilloporidae	*Seriatopora aculeata*	Yes	VU	N/A	33	29	23			*Seriatopora* monophyly shown [42]; closest to *S. stellata* [92]
Pocilloporidae	*Seriatopora caliendrum*	Yes	NT	N/A	173	49	45	12S: EF633601; 16S: EF633601; AT6: EF633601; COI: EF633601; CYB: EF633601		
Pocilloporidae	*Seriatopora dendritica*	Yes	VU	N/A	31	26	19			*Seriatopora* monophyly shown [42]; closest to *S. hystrix* [93]
Pocilloporidae	*Seriatopora guttatus*	Yes	LC	N/A	470	211	205			*Seriatopora* monophyly shown [42]; closest to *S. hystrix* [93]
Pocilloporidae	*Seriatopora hystrix*	Yes	LC	N/A	453	185	177	12S: EF633600; 16S: EF633600; AT6: EF633600; COI: EF633600; CYB: EF633600		
Pocilloporidae	*Seriatopora stellata*	Yes	NT	N/A	197	93	87			*Seriatopora* monophyly shown [42]
Pocilloporidae	*Stylophora danae*	Yes	LC	N/A	509	274	272			*Stylophora* monophyly assumed; closest to *S. pistillata* [92]
Pocilloporidae	*Stylophora kuehlmanni*	Yes	LC	N/A	534	305	304			*Stylophora* monophyly assumed; closest to *S. subseriata* [92]
Pocilloporidae	*Stylophora madagascarensis*	Yes	EN	N/A	5	14	31			*Stylophora* monophyly assumed; closest to *S. kuehlmanni* & *S. subseriata* [93]
Pocilloporidae	*Stylophora mamillata*	Yes	LC	N/A	534	305	304			*Stylophora* monophyly assumed; not close to other *Stylophora* [92]
Pocilloporidae	*Stylophora pistillata*	Yes	NT	N/A	170	46	42	12S: EU400214; 16S: EU400214; AT6: EU400214; COI: EU400214; CYB: EU400214		
Pocilloporidae	*Stylophora subseriata*	Yes	LC	N/A	534	305	304			*Stylophora* monophyly assumed
Pocilloporidae	*Stylophora wellsi*	Yes	NT	N/A	264	153	143			*Stylophora* monophyly assumed
Poritidae	*Alveopora allingi*	Yes	VU	N/A	102	176	167			*Alveopora* monophyly assumed
Poritidae	*Alveopora catalai*	Yes	NT	N/A	330	351	346			*Alveopora* monophyly assumed; closest to *A. allingi* & *A. gigas* [92]
Poritidae	*Alveopora daedalea*	Yes	VU	N/A	102	176	167	12S: EF597088; 16S: AF265592; COI: AB441245; CYB: AB441330		*Alveopora* sp. in GenBank; ancestral branch shared with conspecifics
Poritidae	*Alveopora excelsa*	Yes	EN	N/A	12	56	148			*Alveopora* monophyly assumed
Poritidae	*Alveopora fenestrata*	Yes	VU	N/A	102	176	167			*Alveopora* monophyly assumed; closest to *A. marionensis* & *A. verrilliana* [92]
Poritidae	*Alveopora gigas*	Yes	VU	N/A	102	176	167			*Alveopora* monophyly assumed; closest to *A. allingi* & *A. catalai* [92]
Poritidae	*Alveopora japonica*	Yes	VU	N/A	102	176	167			*Alveopora* monophyly assumed; closest to *A. tizardi* [92]

Family	Species	Reef	Red List	EoE rank	EDGE rank (IUCN100)	EDGE rank (Isaac)	EDGE rank (Pessimistic)	Molecular sources	Morphological sources	Remarks
Poritidae	*Alveopora marionensis*	Yes	VU	N/A	102	176	167			*Alveopora* monophyly assumed; closest to *A. fenestrata* & *A. verrilliana* [92]
Poritidae	*Alveopora minuta*	Yes	EN	N/A	12	56	148			*Alveopora* monophyly assumed; closest to *A. viridis* [93]
Poritidae	*Alveopora ocellata*	Yes	DD	N/A	403	422	414			*Alveopora* monophyly assumed
Poritidae	*Alveopora spongiosa*	Yes	NT	N/A	330	351	346			*Alveopora* monophyly assumed; closest to *A. daedalea* [92]
Poritidae	*Alveopora tizardi*	Yes	LC	N/A	692	555	555			*Alveopora* monophyly assumed
Poritidae	*Alveopora verrilliana*	Yes	VU	N/A	102	176	167			*Alveopora* monophyly assumed
Poritidae	*Alveopora viridis*	Yes	NT	N/A	330	351	346			*Alveopora* monophyly assumed
Poritidae	*Goniopora albiconus*	Yes	VU	N/A	159	319	317		[64]	
Poritidae	*Goniopora burgosi*	Yes	VU	N/A	159	319	317		[64]	
Poritidae	*Goniopora cellulosa*	Yes	VU	N/A	159	319	317		[64]	
Poritidae	*Goniopora ciliatus*	Yes	LC	N/A	771	690	690		[64]	
Poritidae	*Goniopora columna*	Yes	NT	N/A	333	354	349	12S: JF825141; 16S: JF825141; AT6: JF825141; COI: JF825141; CYB: JF825141; ND5: JF825141	[64]	
Poritidae	*Goniopora djiboutiensis*	Yes	LC	N/A	771	690	690		[64]	
Poritidae	*Goniopora eclipsensis*	Yes	LC	N/A	771	690	690		[64]	
Poritidae	*Goniopora fruticosa*	Yes	LC	N/A	771	690	690		[64]	
Poritidae	*Goniopora lobata*	Yes	NT	N/A	406	515	515		[64]	
Poritidae	*Goniopora minor*	Yes	NT	N/A	406	515	515		[64]	
Poritidae	*Goniopora norfolkensis*	Yes	LC	N/A	771	690	690		[64]	
Poritidae	*Goniopora palmensis*	Yes	LC	N/A	771	690	690		[64]	
Poritidae	*Goniopora pandoraensis*	Yes	LC	N/A	771	690	690		[64]	
Poritidae	*Goniopora pearsoni*	Yes	LC	N/A	771	690	690		[64]	
Poritidae	*Goniopora pendulus*	Yes	LC	N/A	771	690	690		[64]	
Poritidae	*Goniopora planulata*	Yes	VU	N/A	159	319	317		[64]	
Poritidae	*Goniopora polyformis*	Yes	VU	N/A	159	319	317		[64]	
Poritidae	*Goniopora savignyi*	Yes	LC	N/A	771	690	690		[64]	
Poritidae	*Goniopora somaliensis*	Yes	LC	N/A	771	690	690		[64]	
Poritidae	*Goniopora stokesi*	Yes	NT	N/A	405	513	513	12S: EF597060; 16S: L76008	[64]	
Poritidae	*Goniopora stutchburyi*	Yes	LC	N/A	771	690	690		[64]	
Poritidae	*Goniopora sultani*	Yes	LC	N/A	771	690	690		[64]	
Poritidae	*Goniopora tenella*	Yes	NT	N/A	406	515	515		[64]	
Poritidae	*Goniopora tenuidens*	Yes	LC	N/A	771	690	690		[64,68]	
Poritidae	*Machadoporites tantillus*	Yes	DD	N/A	699	817	817			Closest to *Goniopora* & *Porites* [76]
Poritidae	*Porites annae*	Yes	NT	N/A	222	115	106	COI: FJ423965	[64]	
Poritidae	*Porites aranetai*	Yes	VU	N/A	241	541	541		[64]	
Poritidae	*Porites arnaudi*	Yes	LC	N/A	820	818	818		[64]	
Poritidae	*Porites astreoides*	Yes	LC	N/A	573	372	364	12S: EF597055; COI: AB441242; CYB: AB441327	[64,68]	
Poritidae	*Porites attenuata*	Yes	VU	N/A	241	541	541		[64]	
Poritidae	*Porites australiensis*	Yes	LC	N/A	820	818	818		[64]	
Poritidae	*Porites baueri*	Yes	DD	N/A	611	757	757		[64]	
Poritidae	*Porites bernardi*	Yes	LC	N/A	820	818	818		[64]	
Poritidae	*Porites branneri*	Yes	NT	N/A	267	159	151	12S: EF597059; COI: AY451380	[64]	
Poritidae	*Porites brighami*	Yes	LC	N/A	820	818	818		[64]	
Poritidae	*Porites cocosensis*	Yes	VU	N/A	241	541	541		[64]	
Poritidae	*Porites colonensis*	Yes	DD	N/A	201	58	53	COI: FJ423972	[64]	
Poritidae	*Porites columnaris*	Yes	LC	N/A	820	818	818		[64]	
Poritidae	*Porites compressa*	Yes	LC	N/A	677	481	475	12S: EF597053; 16S: L76020; COI: FJ423970	[64]	
Poritidae	*Porites cumulatus*	Yes	VU	N/A	241	541	541		[64]	
Poritidae	*Porites cylindrica*	Yes	NT	N/A	435	585	585	COI: FJ423968	[64]	
Poritidae	*Porites decasepta*	Yes	DD	N/A	611	757	757		[64]	
Poritidae	*Porites deformis*	Yes	NT	N/A	519	710	710		[64]	
Poritidae	*Porites densa*	Yes	NT	N/A	519	710	710		[64]	
Poritidae	*Porites desilveri*	Yes	EN	N/A	65	348	485		[64]	
Poritidae	*Porites divaricata*	Yes	LC	N/A	607	433	425	12S: EF597058; COI: FJ423969	[64]	
Poritidae	*Porites duerdeni*	Yes	LC	N/A	677	481	475	COI: FJ423976	[64]	
Poritidae	*Porites echinulata*	Yes	NT	N/A	519	710	710		[64]	
Poritidae	*Porites ericacea*	Yes	DD	N/A	611	757	757		[64]	
Poritidae	*Porites eridani*	Yes	EN	N/A	65	348	485		[64]	
Poritidae	*Porites evermanni*	Yes	DD	N/A	291	165	157	COI: FJ423984	[64]	
Poritidae	*Porites excavata*	Yes	DD	N/A	611	757	757		[64]	
Poritidae	*Porites flavus*	Yes	DD	N/A	611	757	757		[64]	
Poritidae	*Porites furcata*	Yes	LC	N/A	618	445	437	COI: FJ423988	[64]	
Poritidae	*Porites harrisoni*	Yes	NT	N/A	519	710	710		[64]	
Poritidae	*Porites heronensis*	Yes	LC	N/A	820	818	818		[64]	
Poritidae	*Porites horizontalata*	Yes	VU	N/A	241	541	541		[64]	
Poritidae	*Porites latistella*	Yes	LC	N/A	820	818	818		[64]	
Poritidae	*Porites lichen*	Yes	LC	N/A	757	651	651	COI: FJ423963	[64]	

Family	Species	Reef	Red List	EoE rank	EDGE rank (IUCN100)	EDGE rank (Isaac)	EDGE rank (Pessimistic)	Molecular sources	Morphological sources	Remarks
Poritidae	*Porites lobata*	Yes	NT	N/A	158	32	26	16S: AF550372; COI: FJ423973	[64]	
Poritidae	*Porites lutea*	Yes	LC	N/A	794	727	727	COI: AB441243; CYB: AB441328	[64]	
Poritidae	*Porites mayeri*	Yes	LC	N/A	820	818	818		[64]	
Poritidae	*Porites monticulosa*	Yes	LC	N/A	820	818	818		[64]	
Poritidae	*Porites murrayensis*	Yes	NT	N/A	519	710	710		[64]	
Poritidae	*Porites myrmidonensis*	Yes	LC	N/A	820	818	818		[64]	
Poritidae	*Porites napopora*	Yes	VU	N/A	241	541	541		[64]	
Poritidae	*Porites negrosensis*	Yes	NT	N/A	519	710	710		[64]	
Poritidae	*Porites nigrescens*	Yes	VU	N/A	241	541	541		[64]	
Poritidae	*Porites nodifera*	Yes	LC	N/A	820	818	818		[64]	
Poritidae	*Porites okinawensis*	Yes	VU	N/A	142	259	257	12S: JF825142; 16S: JF825142; AT6: JF825142; COI: JF825142; CYB: JF825142; ND5: JF825142	[64]	
Poritidae	*Porites ornata*	Yes	EN	N/A	65	348	485		[64]	
Poritidae	*Porites panamensis*	Yes	LC	N/A	572	371	363	COI: FJ423990	[64]	
Poritidae	*Porites porites*	Yes	LC	N/A	533	302	301	12S: DQ643837; 16S: DQ643837; AT6: DQ643837; COI: DQ643837; CYB: DQ643837; ND5: DQ643837	[64]	
Poritidae	*Porites profundus*	Yes	LC	N/A	820	818	818		[64]	
Poritidae	*Porites pukoensis*	Yes	CR	N/A	40	174	466		[64]	
Poritidae	*Porites randalli*	Yes	DD	N/A	364	337	335	COI: FJ423966	[64]	New species [45]
Poritidae	*Porites rugosa*	Yes	VU	N/A	241	541	541		[64]	
Poritidae	*Porites rus*	Yes	LC	N/A	616	441	433	COI: FJ423979	[64]	
Poritidae	*Porites sillimaniana*	Yes	VU	N/A	241	541	541		[64]	
Poritidae	*Porites solida*	Yes	LC	N/A	795	728	728	COI: FJ423962	[64]	
Poritidae	*Porites somaliensis*	Yes	NT	N/A	519	710	710		[64]	
Poritidae	*Porites stephensoni*	Yes	NT	N/A	519	710	710		[64]	
Poritidae	*Porites studeri*	Yes	LC	N/A	820	818	818		[64]	
Poritidae	*Porites sverdrupi*	Yes	VU	N/A	241	541	541		[64]	
Poritidae	*Porites tuberculosa*	Yes	VU	N/A	241	541	541		[64]	
Poritidae	*Porites vaughani*	Yes	LC	N/A	820	818	818		[64]	
Poritidae	*Poritipora paliformis*	Yes	VU	N/A	299	681	681			Poritidae monophyly assumed (excluding *Alveopora*)
Poritidae	*Stylaraea punctata*	Yes	DD	Potential	676	805	805			Closest to *Porites* [90]
Rhizangiidae	*Astrangia poculata*	Yes	LC	N/A	720	602	602	12S: DQ643832; AT6: DQ643832; COI: DQ643832; CYB: DQ643832		*Astrangia* sp. in GenBank; terminal branch shared with conspecifics
Rhizangiidae	*Astrangia rathbuni*	No	N/A	N/A	N/A	N/A	N/A		[68]	
Siderastreidae	*Anomastraea irregularis*	Yes	VU	1	19	11	7	COI: AM494870	[67]	
Siderastreidae	*Coscinaraea columna*	Yes	LC	N/A	530	298	297	COI: AB441210; CYB: AB441295	[67]	
Siderastreidae	*Coscinaraea crassa*	Yes	NT	N/A	336	365	357		[67]	
Siderastreidae	*Coscinaraea exesa*	Yes	LC	N/A	705	576	576		[67]	
Siderastreidae	*Coscinaraea hahazimaensis*	Yes	VU	N/A	112	189	181		[67]	
Siderastreidae	*Coscinaraea marshae*	Yes	LC	N/A	705	576	576		[67]	
Siderastreidae	*Coscinaraea mcneilli*	Yes	LC	N/A	705	576	576		[67]	
Siderastreidae	*Coscinaraea monile*	Yes	LC	N/A	705	576	576		[67]	
Siderastreidae	*Coscinaraea wellsi*	Yes	LC	N/A	565	355	350	COI: AM494861		Fungiidae clade [71]
Siderastreidae	*Craterestrea levis*	Yes	LC	N/A	718	600	600			Closest to *Coscinaraea* [80]
Siderastreidae	*Horastrea indica*	Yes	VU	4	14	6	4	COI: AM494864	[67]	
Siderastreidae	*Psammocora albopicta*	Yes	DD	N/A	287	163	155	COI: FM865871	[67]	
Siderastreidae	*Psammocora contigua*	Yes	NT	N/A	145	25	18	16S: AF550371; COI: AB441209; CYB: AB441294	[67]	
Siderastreidae	*Psammocora decussata*	Yes	DD	N/A	431	460	452		[67]	*Psammocora* monophyly shown [72]
Siderastreidae	*Psammocora digitata*	Yes	NT	N/A	203	112	104	COI: AM494855	[67]	
Siderastreidae	*Psammocora explanulata*	Yes	LC	N/A	565	355	350	COI: AM494845		Fungiidae clade [71]
Siderastreidae	*Psammocora haimeana*	Yes	LC	N/A	488	255	253	COI: FM865874	[67]	
Siderastreidae	*Psammocora interstinctus*	Yes	DD	N/A	431	460	452		[67]	*Psammocora* monophyly shown [72]
Siderastreidae	*Psammocora nierstraszi*	Yes	LC	N/A	399	119	110	COI: AM494851	[67]	
Siderastreidae	*Psammocora obtusangula*	Yes	NT	N/A	343	382	374		[67]	
Siderastreidae	*Psammocora profundacella*	Yes	LC	N/A	400	120	111	COI: AM494853	[67]	
Siderastreidae	*Psammocora ramosa*	Yes	DD	N/A	431	460	452		[67]	*Psammocora* monophyly shown [72]
Siderastreidae	*Psammocora stellata*	Yes	VU	N/A	116	204	197		[67]	*Psammocora* monophyly shown [72]
Siderastreidae	*Psammocora superficialis*	Yes	LC	N/A	717	598	598		[67]	
Siderastreidae	*Psammocora vaughani*	Yes	NT	N/A	343	382	374		[67]	

Family	Species	Reef	Red List	EoE rank	EDGE rank (IUCN100)	EDGE rank (Isaac)	EDGE rank (Pessimistic)	Molecular sources	Morphological sources	Remarks
Siderastreidae	*Psammocora verrilli*	Yes	DD	N/A	431	460	452		[67]	*Psammocora* monophyly shown [72]
Siderastreidae	*Pseudosiderastrea tayami*	Yes	NT	ED	174	55	52	COI: AM494866	[67]	
Siderastreidae	*Siderastrea glynni*	Yes	CR	N/A	4	4	46		[67]	*Siderastrea* monophyly shown [82]
Siderastreidae	*Siderastrea radians*	Yes	LC	N/A	374	75	70	12S: DQ643838; 16S: DQ643838; AT6: DQ643838; COI: DQ643838; CYB: DQ643838; ND5: DQ643838	[67]	
Siderastreidae	*Siderastrea savignyana*	Yes	LC	N/A	362	64	59	COI: AB441215; CYB: AB441300	[67]	
Siderastreidae	*Siderastrea siderea*	Yes	LC	N/A	387	91	85	12S: EF597067; COI: AB441211; CYB: AB441296	[67]	
Siderastreidae	*Siderastrea stellata*	Yes	DD	N/A	187	38	33	COI: AB441213; CYB: AB441298	[67,68]	
Stenocyathidae	*Stenocyathus vermiformis*	No	N/A	N/A	N/A	N/A	N/A	COI: HM018619		
Trachyphylliidae	*Trachyphyllia geoffroyi*	Yes	NT	ED	225	122	112	COI: AB117287; CYB: AB117372		Clade VII-B monophyly shown [81]
Turbinoliidae	*Alatotrochus rubescens*	No	N/A	N/A	N/A	N/A	N/A		[62]	
Turbinoliidae	*Australocyathus vincentinus*	No	N/A	N/A	N/A	N/A	N/A		[62]	
Turbinoliidae	*Conocyathus formosus*	No	N/A	N/A	N/A	N/A	N/A		[62]	
Turbinoliidae	*Conocyathus gracilis*	No	N/A	N/A	N/A	N/A	N/A		[62]	
Turbinoliidae	*Conocyathus zelandiae*	No	N/A	N/A	N/A	N/A	N/A		[62]	
Turbinoliidae	*Cryptotrochus brevipalus*	No	N/A	N/A	N/A	N/A	N/A		[62]	
Turbinoliidae	*Cryptotrochus carolinensis*	No	N/A	N/A	N/A	N/A	N/A		[62]	
Turbinoliidae	*Cryptotrochus javanus*	No	N/A	N/A	N/A	N/A	N/A		[62]	
Turbinoliidae	*Cyathotrochus herdmani*	No	N/A	N/A	N/A	N/A	N/A		[62]	
Turbinoliidae	*Cyathotrochus nascornatus*	No	N/A	N/A	N/A	N/A	N/A		[62]	
Turbinoliidae	*Cyathotrochus pileus*	No	N/A	N/A	N/A	N/A	N/A	12S: EF597069; COI: HM018623	[62]	
Turbinoliidae	*Deltocyathoides orientalis*	No	N/A	N/A	N/A	N/A	N/A		[62]	
Turbinoliidae	*Deltocyathoides stimpsonii*	No	N/A	N/A	N/A	N/A	N/A		[62]	
Turbinoliidae	*Dunocyathus parasiticus*	No	N/A	N/A	N/A	N/A	N/A		[62]	
Turbinoliidae	*Dunocyathus wallaceae*	No	N/A	N/A	N/A	N/A	N/A		[62]	
Turbinoliidae	*Endocyathopora laticostata*	No	N/A	N/A	N/A	N/A	N/A		[62]	
Turbinoliidae	*Foveolocyathus alternans*	No	N/A	N/A	N/A	N/A	N/A		[62]	
Turbinoliidae	*Foveolocyathus kitsoni*	No	N/A	N/A	N/A	N/A	N/A		[62]	
Turbinoliidae	*Foveolocyathus parkeri*	No	N/A	N/A	N/A	N/A	N/A		[62]	
Turbinoliidae	*Foveolocyathus verconis*	No	N/A	N/A	N/A	N/A	N/A		[62]	
Turbinoliidae	*Holcotrochus crenulatus*	No	N/A	N/A	N/A	N/A	N/A		[62]	
Turbinoliidae	*Holcotrochus scriptus*	No	N/A	N/A	N/A	N/A	N/A		[62]	
Turbinoliidae	*Idiotrochus alatus*	No	N/A	N/A	N/A	N/A	N/A		[62]	
Turbinoliidae	*Idiotrochus emarciatus*	No	N/A	N/A	N/A	N/A	N/A		[62]	
Turbinoliidae	*Idiotrochus kikutii*	No	N/A	N/A	N/A	N/A	N/A		[62]	
Turbinoliidae	*Kionotrochus suteri*	No	N/A	N/A	N/A	N/A	N/A		[62]	
Turbinoliidae	*Notocyathus conicus*	No	N/A	N/A	N/A	N/A	N/A	12S: EF597061; 16S: AF265584	[62]	*Notocyathus* sp. in GenBank; ancestral branch shared with conspecifics
Turbinoliidae	*Notocyathus venustus*	No	N/A	N/A	N/A	N/A	N/A		[62]	
Turbinoliidae	*Peponocyathus dawsoni*	No	N/A	N/A	N/A	N/A	N/A		[62]	
Turbinoliidae	*Peponocyathus folliculus*	No	N/A	N/A	N/A	N/A	N/A		[62]	
Turbinoliidae	*Peponocyathus minimus*	No	N/A	N/A	N/A	N/A	N/A		[62]	
Turbinoliidae	*Platytrochus compressus*	No	N/A	N/A	N/A	N/A	N/A		[62]	
Turbinoliidae	*Platytrochus hastatus*	No	N/A	N/A	N/A	N/A	N/A		[62]	
Turbinoliidae	*Platytrochus laevigatus*	No	N/A	N/A	N/A	N/A	N/A		[62]	
Turbinoliidae	*Platytrochus parisepta*	No	N/A	N/A	N/A	N/A	N/A		[62]	
Turbinoliidae	*Pleotrochus venustus*	No	N/A	N/A	N/A	N/A	N/A		[62]	
Turbinoliidae	*Pleotrochus zibrowii*	No	N/A	N/A	N/A	N/A	N/A		[62]	
Turbinoliidae	*Pseudocyathoceras avis*	No	DD	N/A	N/A	N/A	N/A		[62]	

Family	Species	Reef	Red List	EoE rank	EDGE rank (IUCN100)	EDGE rank (Isaac)	EDGE rank (Pessimistic)	Molecular sources	Morphological sources	Remarks
Turbinoliidae	*Sphenotrochus andrewianus*	No	N/A	N/A	N/A	N/A	N/A		[62]	
Turbinoliidae	*Sphenotrochus aurantiacus*	No	N/A	N/A	N/A	N/A	N/A		[62]	
Turbinoliidae	*Sphenotrochus auritus*	No	N/A	N/A	N/A	N/A	N/A		[62]	
Turbinoliidae	*Sphenotrochus cuneolus*	No	N/A	N/A	N/A	N/A	N/A		[62]	
Turbinoliidae	*Sphenotrochus evexicostatus*	No	N/A	N/A	N/A	N/A	N/A		[62]	
Turbinoliidae	*Sphenotrochus excavatus*	No	N/A	N/A	N/A	N/A	N/A		[62]	
Turbinoliidae	*Sphenotrochus gardineri*	No	N/A	N/A	N/A	N/A	N/A		[62]	
Turbinoliidae	*Sphenotrochus gilchristi*	No	N/A	N/A	N/A	N/A	N/A		[62]	
Turbinoliidae	*Sphenotrochus hancocki*	No	N/A	N/A	N/A	N/A	N/A		[62]	
Turbinoliidae	*Sphenotrochus imbricaticostatus*	No	N/A	N/A	N/A	N/A	N/A		[62]	
Turbinoliidae	*Sphenotrochus lindstroemi*	No	N/A	N/A	N/A	N/A	N/A		[62]	
Turbinoliidae	*Sphenotrochus ralphae*	No	N/A	N/A	N/A	N/A	N/A		[62]	
Turbinoliidae	*Sphenotrochus squiresi*	No	N/A	N/A	N/A	N/A	N/A		[62]	
Turbinoliidae	*Thrypticotrochus petterdi*	No	N/A	N/A	N/A	N/A	N/A		[62]	
Turbinoliidae	*Trematotrochus corbicula*	No	N/A	N/A	N/A	N/A	N/A		[62]	
Turbinoliidae	*Trematotrochus hedleyi*	No	N/A	N/A	N/A	N/A	N/A		[62]	
Turbinoliidae	*Tropidocyathus labidus*	No	N/A	N/A	N/A	N/A	N/A	12S: EF597062; 16S: AF265585	[62]	
Turbinoliidae	*Tropidocyathus lessoni*	No	N/A	N/A	N/A	N/A	N/A	COI: HM018669	[62]	
Turbinoliidae	*Turbinolia stephensoni*	No	N/A	N/A	N/A	N/A	N/A		[62]	
Discosomatidae (outgroup)	*Discosoma*	N/A	N/A	N/A	N/A	N/A	N/A	12S: DQ643965; 16S: DQ643965; AT6: DQ643965; COI: DQ643965; CYB: DQ643965; ND5: DQ643965	[68]	
Ricordeidae (outgroup)	*Ricordea florida*	N/A	N/A	N/A	N/A	N/A	N/A	12S: DQ640648; 16S: DQ640648; AT6: DQ640648; COI: DQ640648; CYB: DQ640648; ND5: DQ640648	[68]	
